# Supplementary material for: Antagonism between viral infection and innate immunity at the single-cell level
Source: PLoS Pathog. 2023 Sep 5;19(9):e1011597. doi: 10.1371/journal.ppat.1011597 (PMC10503725; doi:10.1371/journal.ppat.1011597)
Supplement: S1 Appendix — (PDF) [file ppat.1011597.s001.pdf]

S1 Appendix

featuring the article

Antagonism between viral infection and innate immunity at the single-cell level

by Frederic Grabowski, Marek Kochańczyk, Zbigniew Korwek,
Maciej Czerkies, Wiktor Prus, and Tomasz Lipniacki

Contents

Figure A. Progression of RSV infection without IFNβ pre-stimulation 2

Figure B. Viral infection triggers an innate immune signaling cascade 3

Figure C. STAT1/2 respond quickly but IFNβ-stimulated proteins accumulate and degrade slowly 4

Figure D. IFNβ/λ-induced STAT1/2 signaling attenuates viral infection 6

Figure E. Pre-stimulation with IFNβ impedes virus spread 7

Figure F. Images from experiment and snapshots from simulation, infection at an MOI of 1 8

Figure G. Colocalization of nuclear RelA and IRF3 9

Figure H. Influence of brefeldin A on IFNβ detection 10

Figure I. Viral proteins accumulate more slowly in cells protected by ISGs (model) 11

Figure J. Lack of dependence of the signed Kolmogorov–Smirnov statistics for (pSTAT | RSV proteins) on remaining inhibition strengths (model) 12

Table A. Model equations and parameters 13

References

In Figs A, C, D, and E we juxtapose model predictions with quantified experimental results reported in the following articles, as detailed in figure legends. For the reader's convenience, we show corresponding Western blot replicates (not shown in these papers).

1. Czerkies M, Kochańczyk M, Korwek Z, Prus W & Lipniacki T (2022) Respiratory syncytial virus protects bystander cells against influenza A virus infection by triggering secretion of type I and type III Interferons. *J Virol* **96**(22): e0134122, doi:10.1128/jvi.01341-22.

2. Korwek Z, Czerkies M, Jaruszewicz-Błońska J, Prus W, Kosiuk I, Kochańczyk M & Lipniacki T (2023) Non-self RNA rewires IFNβ signaling: A mathematical model of the innate immune response. *bioRxiv* doi:10.1101/2022.01.30.478391 (preprint).

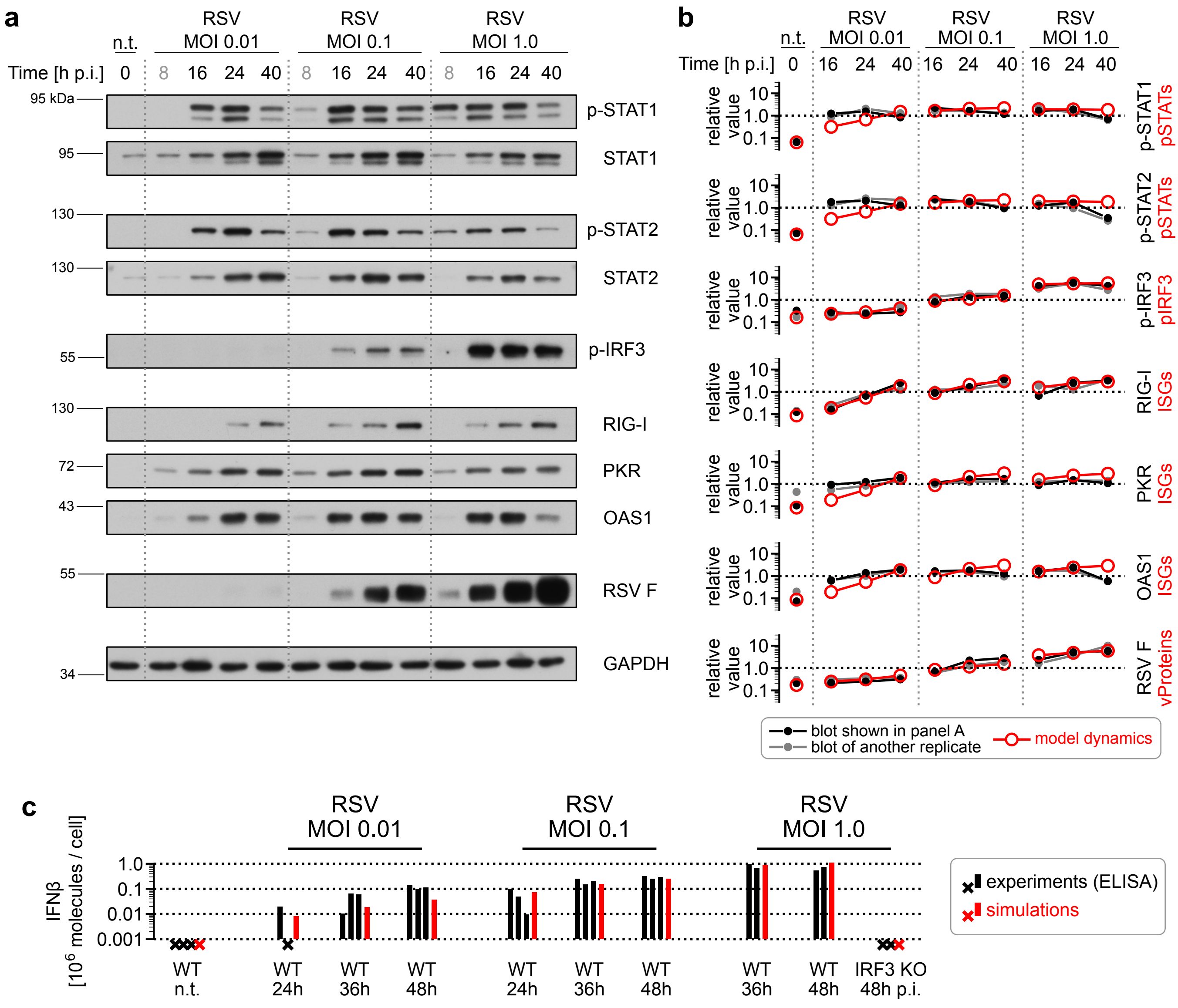

Figure A. Progression of RSV infection without IFNβ pre-stimulation.

a, b Progression of RSV infection at MOIs of 0.01, 0.1, and 1 in A549 cells. Western blot analysis (a, b) is juxtaposed with model simulations (b). Time point of 8 h p.i. lacks experimental replicates and is thus not included in panel C.

c Infected cells produce and secrete IFNβ. Results of ELISA for A549 cells infected with RSV at MOIs of 0.01, 0.1, and 1 (black) are juxtaposed with model predictions (red). Experiments with samples below the limit of detection as well as simulation results with no IFN are marked with crosses. For MOIs of 0.01 and 0.1 we used data from Czerkies *et al.* [1] (see Figure 1A therein).

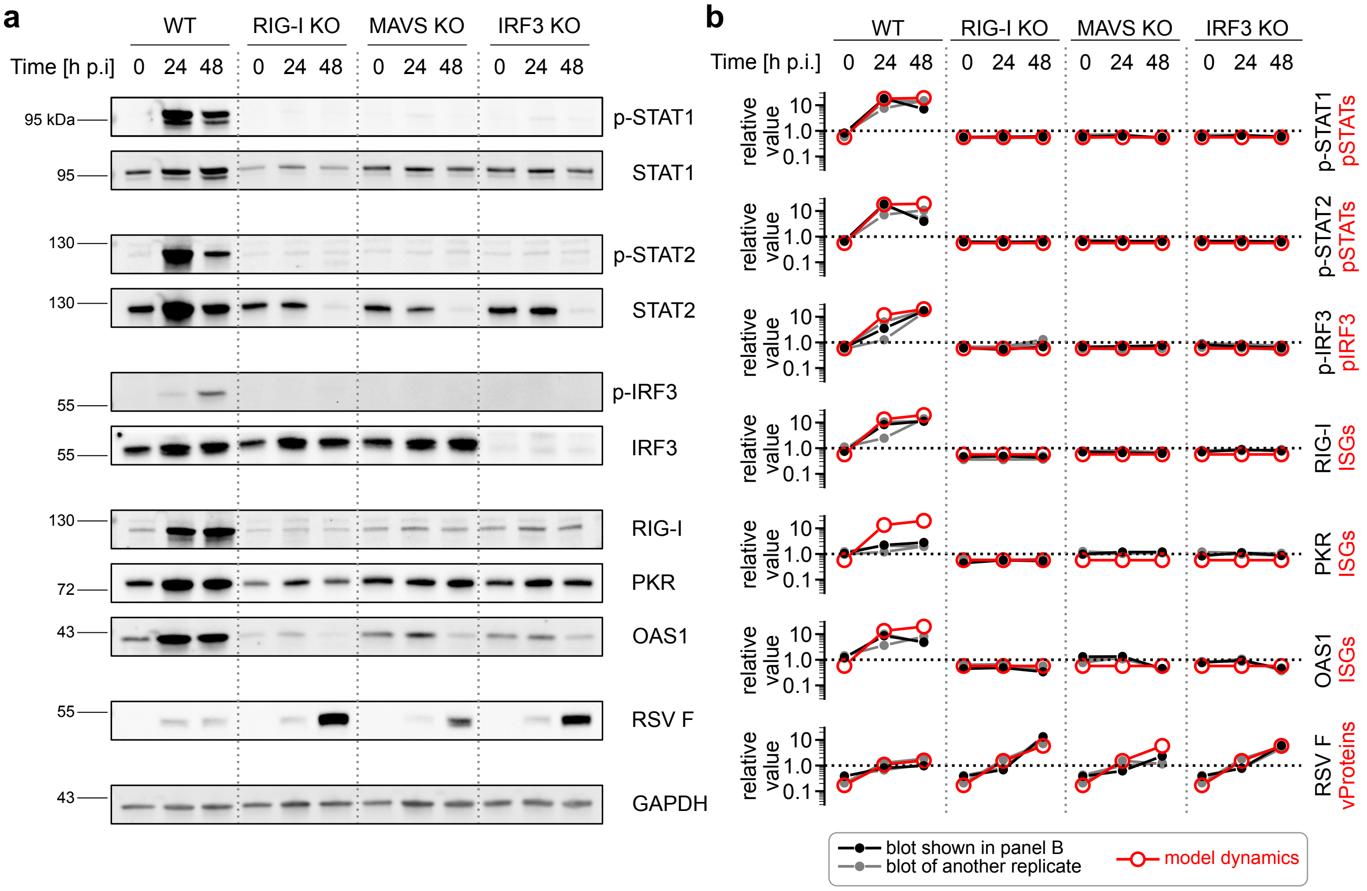

Figure B. Viral infection triggers an innate immune signaling cascade.

a, b RSV activates multiple components of the innate immune signaling. Western blot analysis of A549 cells infected with RSV at an MOI of 0.1 (a, b) is juxtaposed with model simulations (b). In panel b, black circles and connecting lines are quantifications of blots shown in panel a, gray filled circles and connecting lines are quantifications of blots from another experimental replicate, and red circles and connecting lines were obtained by sampling the average model trajectory at experimental time points. All KO cell lines were simulated by disabling activation of `pIRF3`.

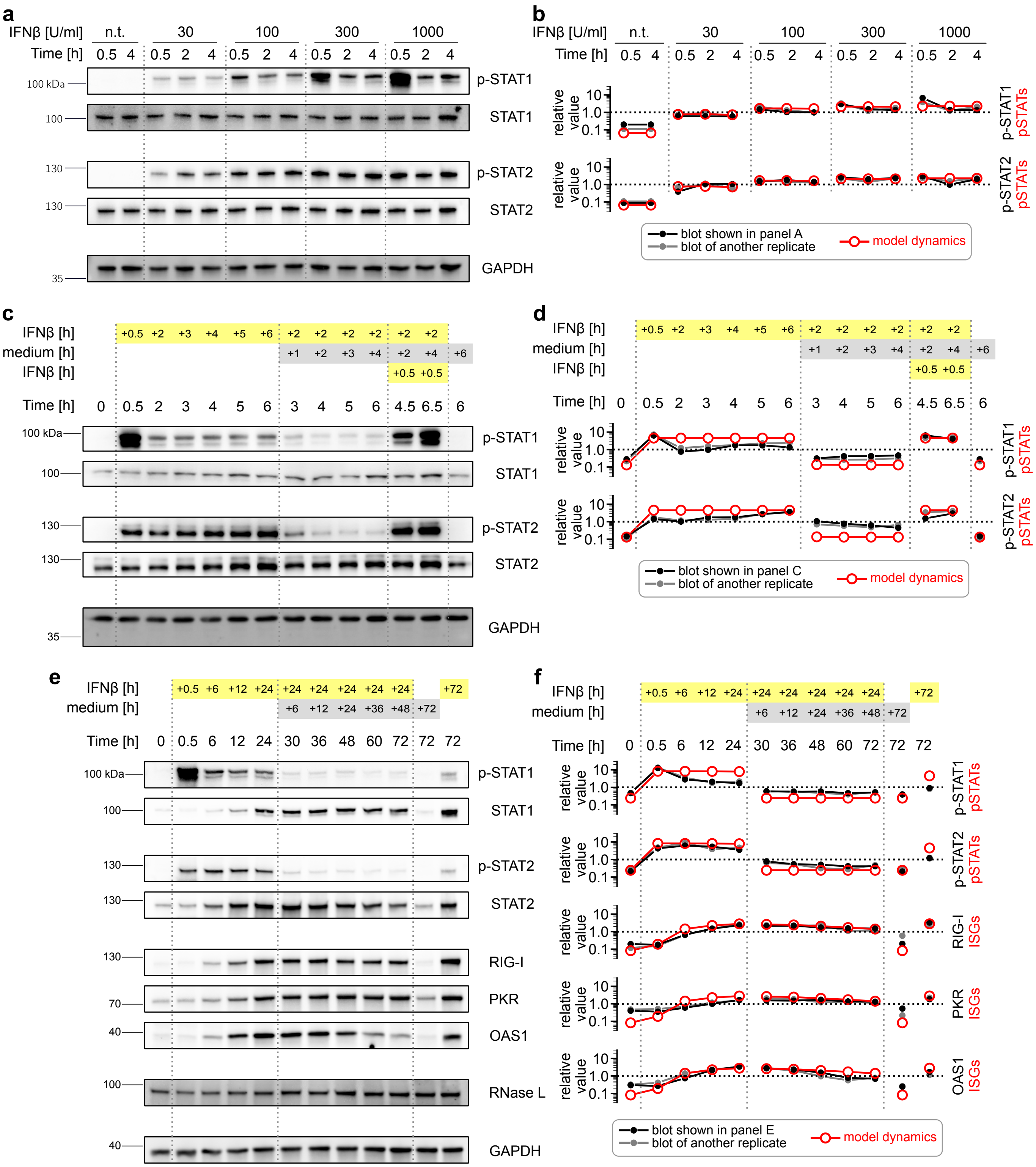

Figure C. STAT1/2 respond quickly but IFNβ-stimulated proteins accumulate and degrade slowly.

a, **b** STAT1/2 are activated quickly and their amplitude depends on IFNβ concentration up to 1000 U/ml. Western blot analysis of A549 cells (a, b) is juxtaposed with model simulations (b). Western blot data originates from Korwek *et al.* [2] (see Fig. S2C therein). The comparison between model and experiment in panel B is based on the conversion 1000 U/ml = 5.5 ng/ml for the IFNβ stock used.

▲ *(continued from the previous page)*

c, **d** IFNβ is required to sustain STAT1/2 activity. A549 cells were stimulated with IFNβ (1000 U/ml) according to the indicated protocols: for example, in the next to last column, cells were stimulated with IFNβ for 2 h, then IFNβ was washed out by medium replacement and cells were cultured in a fresh medium for subsequent 4 h, and finally IFNβ was added for 0.5 h (protocol total duration is 6.5 h). Western blot analysis (c, d) is juxtaposed with model simulations (d). Western blot data originates from Korwek *et al.* [2] (see Fig. 3A therein).

e, **f** IFNβ-stimulated proteins accumulate and degrade slowly. A549 WT cells were stimulated with IFNβ (1000 U/ml) according to the indicated protocols. Western blot analysis (e, f) is juxtaposed with model simulations (f).

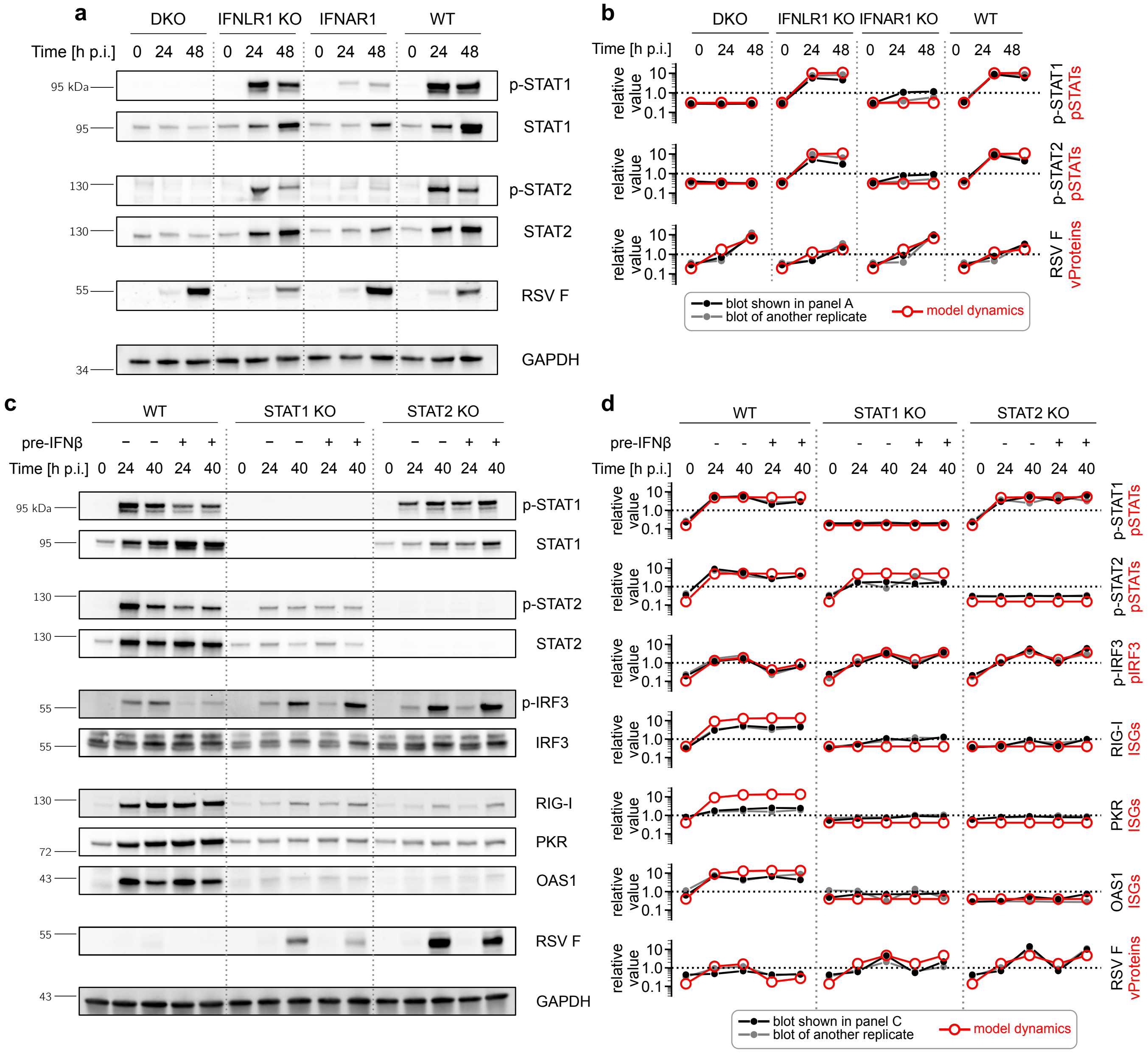

Figure D. IFNβ/λ-induced STAT1/2 signaling attenuates viral infection.

a, **b** Both IFNβ and, to a lesser degree, IFNλ activate STAT1 and STAT2. Western blot analysis of A549 cells infected with RSV at an MOI of 0.1 (a, b) is juxtaposed with model simulations (b). Western blot data originates from Czerkies *et al.* [1] (see Figure 1D therein). Both the IFNAR1 KO and IFNAR1–INFLR1 double KO cell lines were simulated by disabling the forward transition of `pSTATs`.

c, **d** Activation of STAT1/2 attenuates the spread of RSV infection. Western blot analysis of A549 cells infected with RSV at an MOI of 0.1 (c, d) is juxtaposed with model simulations (d). Both the STAT1 KO and STAT2 KO cell lines were simulated by disabling the forward transition of `pSTATs`.

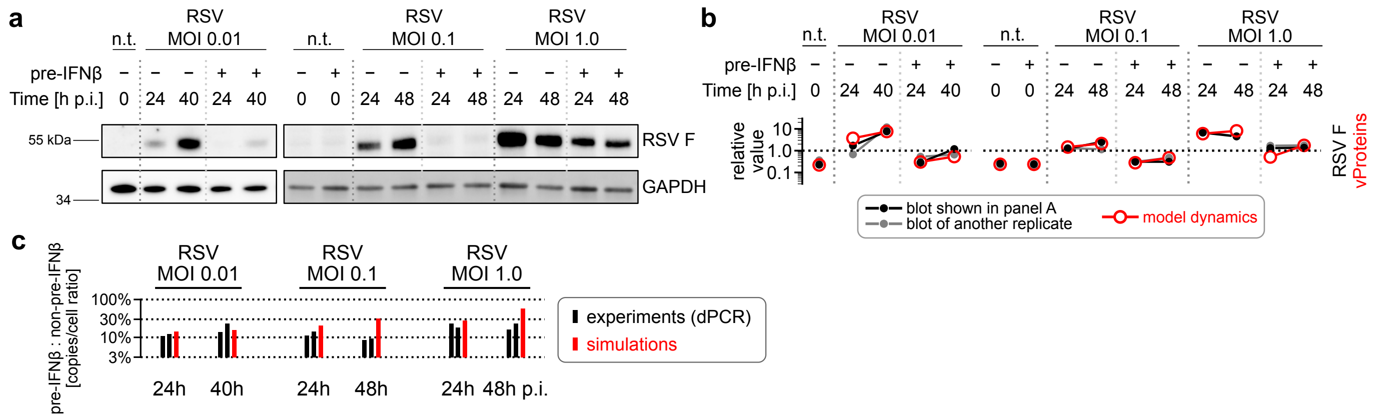

Figure E. Pre-stimulation with IFNβ impedes virus spread.

a, **b** Propagation of RSV infection and signaling without or with prior IFNβ pre-stimulation (1000 U/ml). A549 cells were infected with RSV at MOI of 0.01, 0.1, and 1. Western blot analysis (a, b) is juxtaposed with model simulations (b). For MOIs 0.1, 1 we use experimental data shown in Czerkies *et al.* [1] (as a part of Figure 2C therein).

c Viral load in RSV infection without or with prior IFNβ pre-stimulation (1000 U/ml). Digital PCR results for A549 cells infected with RSV at MOIs of 0.01, 0.1, and 1 (black) are juxtaposed with model predictions (red). For an MOI of 0.1 and 1 we use data shown in Czerkies *et al.* [1] (see Figure 2A therein).

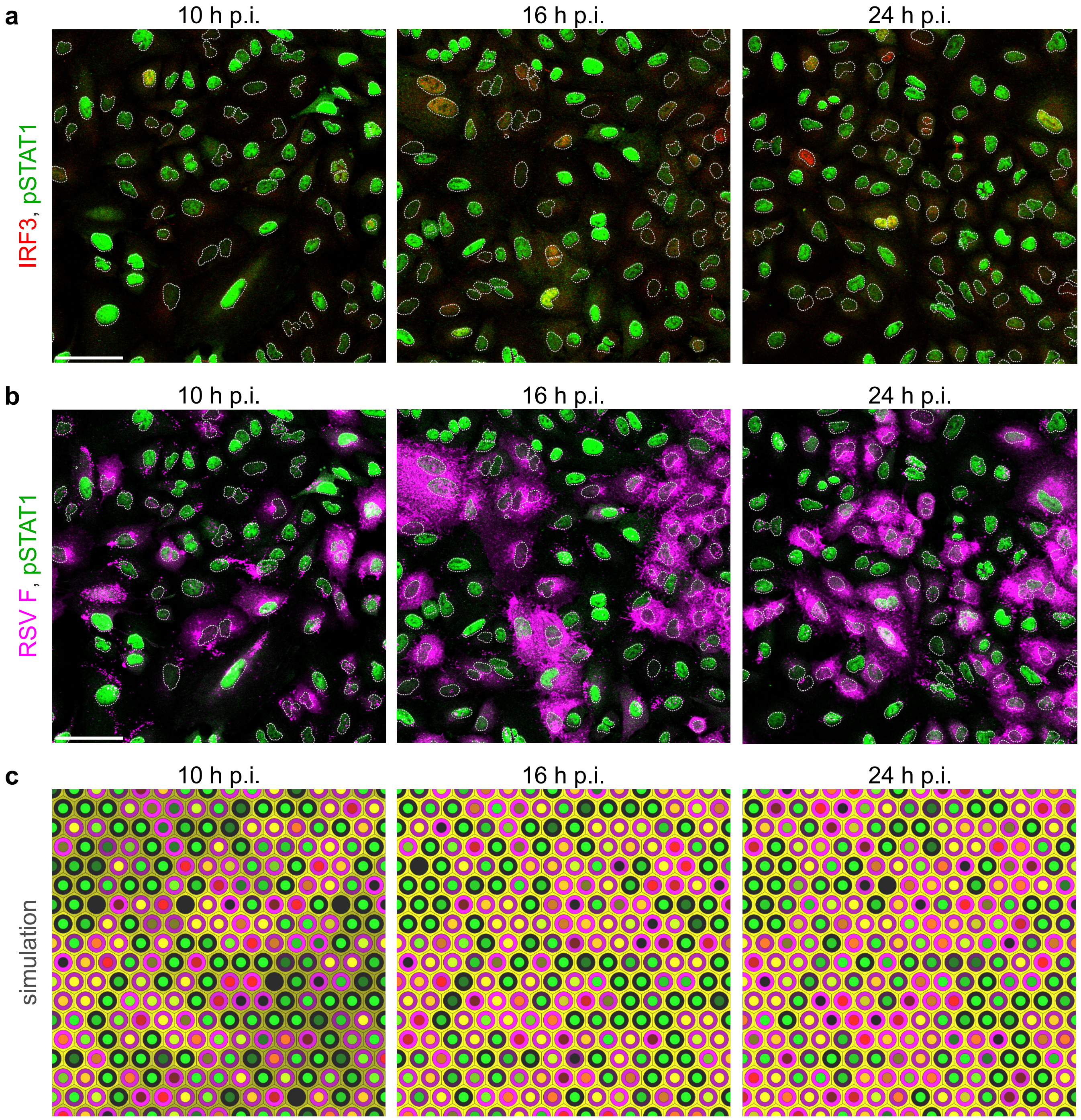

Figure F. Images from experiment and snapshots from simulation, infection at an MOI of 1.

a, **b** A549 cells 10, 16, and 24 hours post infection (p.i.) with RSV at an MOI of 1. IRF3 – red (only in panel a), RSV – magenta (only in panel b), p-STAT1 – green. White dotted lines are nuclear outlines determined based on DAPI counterstaining (channel not shown). Scale bars, 50 µm.

c Snapshots from a simulation of infection at an MOI of 1 in a compact monolayer of cells (subpanels show small fragments of a simulated 100 × 100 lattice).

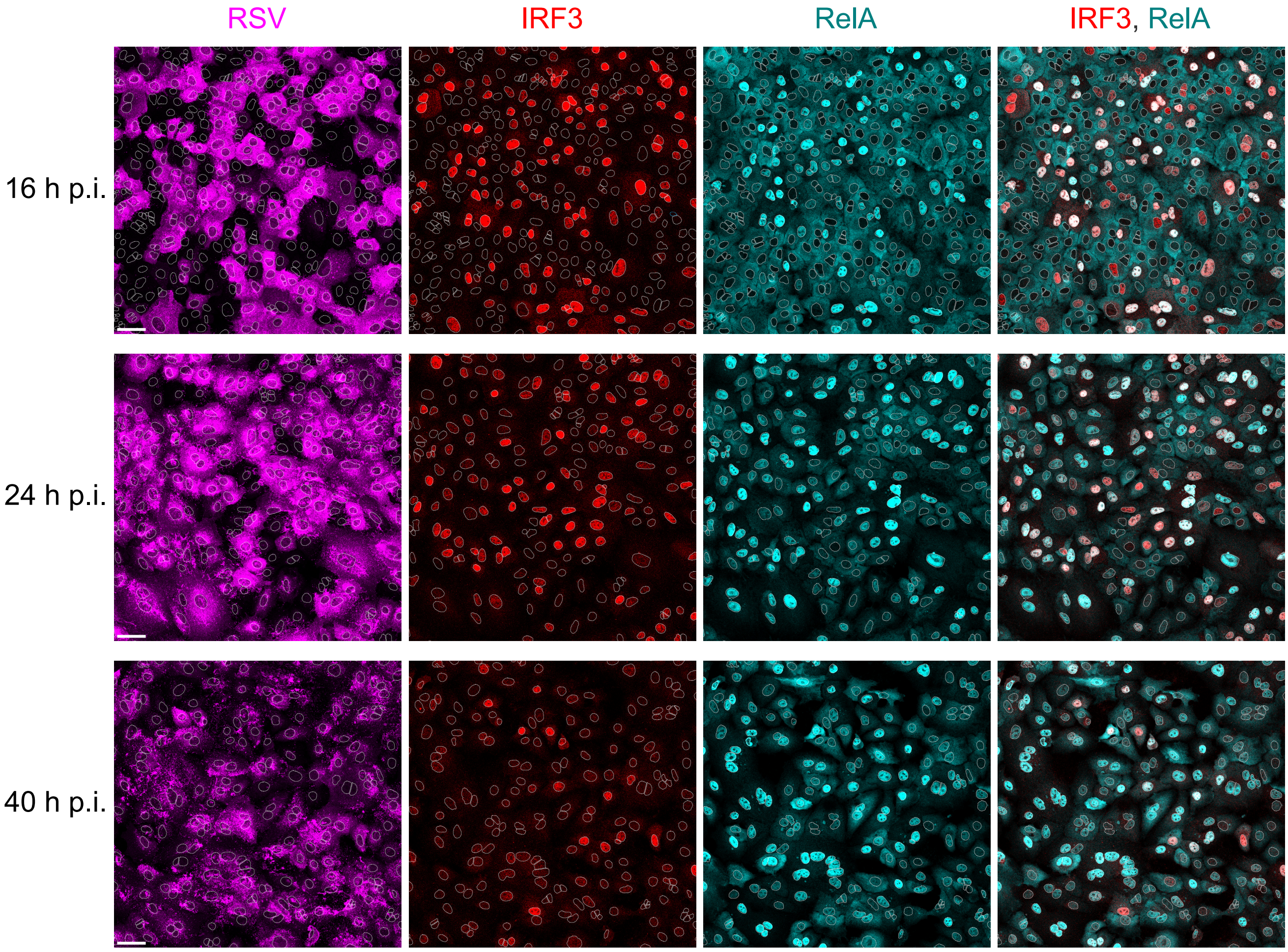

Figure G. Colocalization of nuclear RelA and IRF3.

A549 cells 10, 16, and 24 hours post infection (p.i.) with RSV at an MOI of 1. RSV – magenta, IRF3 – red, RelA (a subunit of NF-κB) – cyan. White dotted lines are nuclear outlines determined based on DAPI counterstaining (channel not shown). Scale bars, 50 μm.

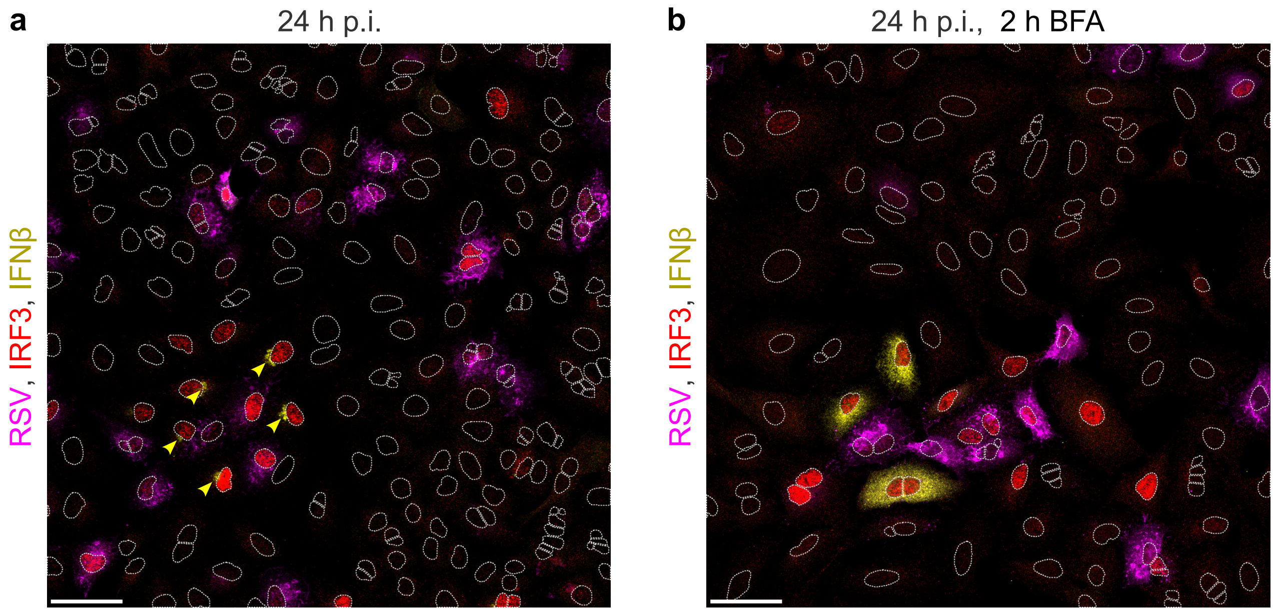

Figure H. Influence of brefeldin A on IFNβ detection.

a, **b** A549 cells 10, 16, and 24 hours post infection (p.i.) with RSV at an MOI of 0.01. IRF3 – red, RSV – magenta, IFNβ – yellow. In panel a, small yellow arrows indicate cells secreting IFNβ. In panel b, a 2-hour-long treatment with brefeldin A (BFA) was used before cell fixation. In both panels, white dotted lines are nuclear outlines determined based on DAPI counterstaining (channel not shown). Scale bars, 50 μm.

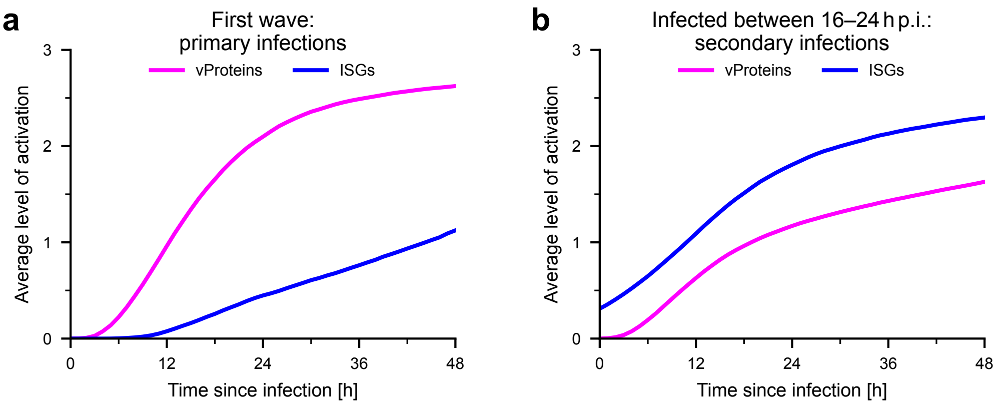

Figure I. Viral proteins accumulate more slowly in cells protected by ISGs (model).

a, **b** Average status of `vProteins` and `ISGs` as a function of time since the infection of individual cells. A simulated cell culture was infected at an MOI of 0.01. Panel a shows primarily infected cells, whereas panel b shows cells that were infected in between 16 and 24 h post infection of the simulated cell culture (secondary infections). In panel a, the time axis shows absolute time post infection; in panel b, time axes of individual cells were aligned to begin at their particular time of infection.

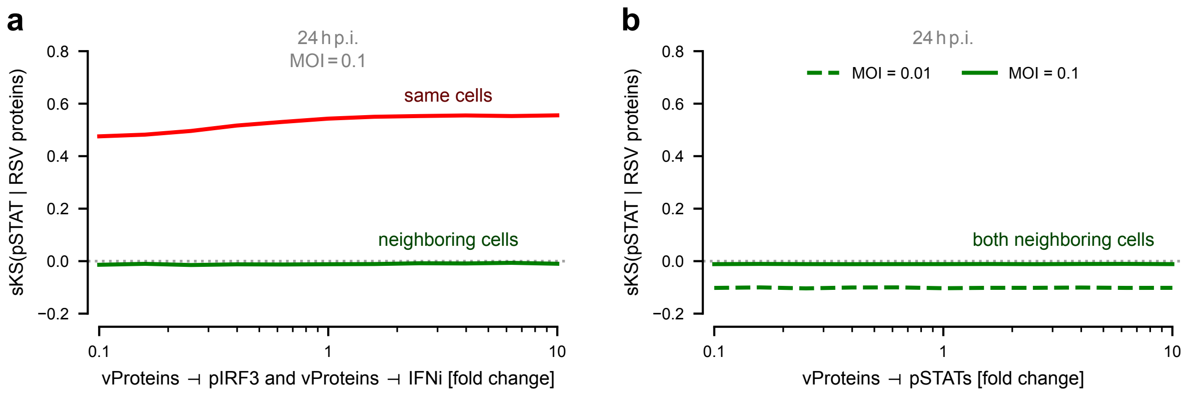

Figure J. Lack of dependence of the signed Kolmogorov–Smirnov statistics for (pSTAT | RSV proteins) on remaining inhibition strengths (model).

a ‘Same cells’ and ‘neighboring cells’ sKS statistics at 24 h p.i. and MOI = 0.1 as a function of the strengths of `vProteins ⊣ pIRF3` and `vProteins ⊣ IFNi` inhibitions (both strengths were varied simultaneously).

b ‘Neighboring cells’ sKS statistics at 24 h p.i., for two MOIs (0.01, 0.1) as a function of the strength of the `vProteins ⊣ pSTATs` inhibition.

Table A. Model equations and parameters.

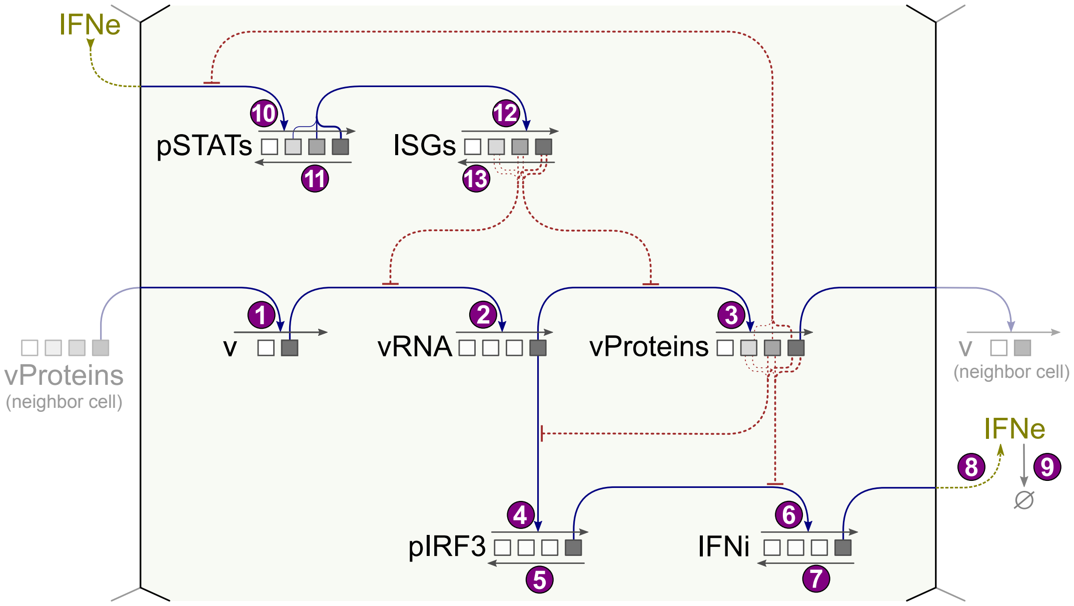

Process	Rate	Parameter values
(1) infection	[v = 0] × v_incr × (*# of neighbors with* vProteins = 3)	v_incr = 0.25 / h
(2) vRNA forward	[vRNA < 3] × v × vrna_incr / (1 + isg_inh_vrna × ISGs)	vrna_incr = 0.5 / h<br>isg_inh_vrna = 2.0
(3) vProteins forward	[vProteins < 3] × [vRNA = 3] × vprot_incr / (1 + isg_inh_vprot × ISGs)	vprot_incr = 0.167 / h<br>isg_inh_vprot = 2.0
(4) pIRF3 forward	[pIRF3 < 3] × [vRNA = 3] × pirf3_incr / (1 + vprot_inh_pirf3 × vProteins)	pirf3_incr = 0.75 / h<br>vprot_inh_pirf3 = 3.0
(5) pIRF3 backward	[pIRF3 > 0] × pirf3_decr	pirf3_decr = 0.125 / h
(6) IFNi forward	[IFNi < 3] × [pIRF3 = 3] × ifni_incr / (1 + vprot_inh_ifni × vProteins)	ifni_incr = 1.0 / h<br>vprot_inh_ifni = 2
(7) IFNi backward	[IFNi > 0] × ifni_decr	ifni_decr = 0.25 / h
(8) increase of IFNe	[IFNi = 3] × k_ifn_sec	k_ifn_sec = 5e5 / h
(9) decrease of IFNe	q_ifne	q_ifne = 1 / day
(10) pSTATs forward	[pSTATs < 3] × pstat_incr × IFNe / (mm_pstat + IFNe) / (1 + vprot_inh_pstat × vProtein	pstat_incr = 40.0 / h<br>mm_pstat = 500<br>vprot_inh_pstat = 1.5
(11) pSTATs backward	[pSTATs > 0] × pstat_decr	pstat_decr = 10.0 / h
(12) ISGs forward	[ISGs < 3] × isg_incr × (pSTATs / 3)	isg_incr = 0.3 / h
(13) ISGs backward	[ISGs > 0] × isg_decr	isg_decr = 0.033 / h

In rate expressions, [*condition*] is defined to be 1 if *condition* is true and 0 otherwise (Iverson bracket).
