# Supplementary figures and images for: Antagonism between viral infection and innate immunity at the single-cell level
Source: PLoS Pathog. 2023 Sep 5;19(9):e1011597. doi: 10.1371/journal.ppat.1011597 (PMC10503725; doi:10.1371/journal.ppat.1011597)

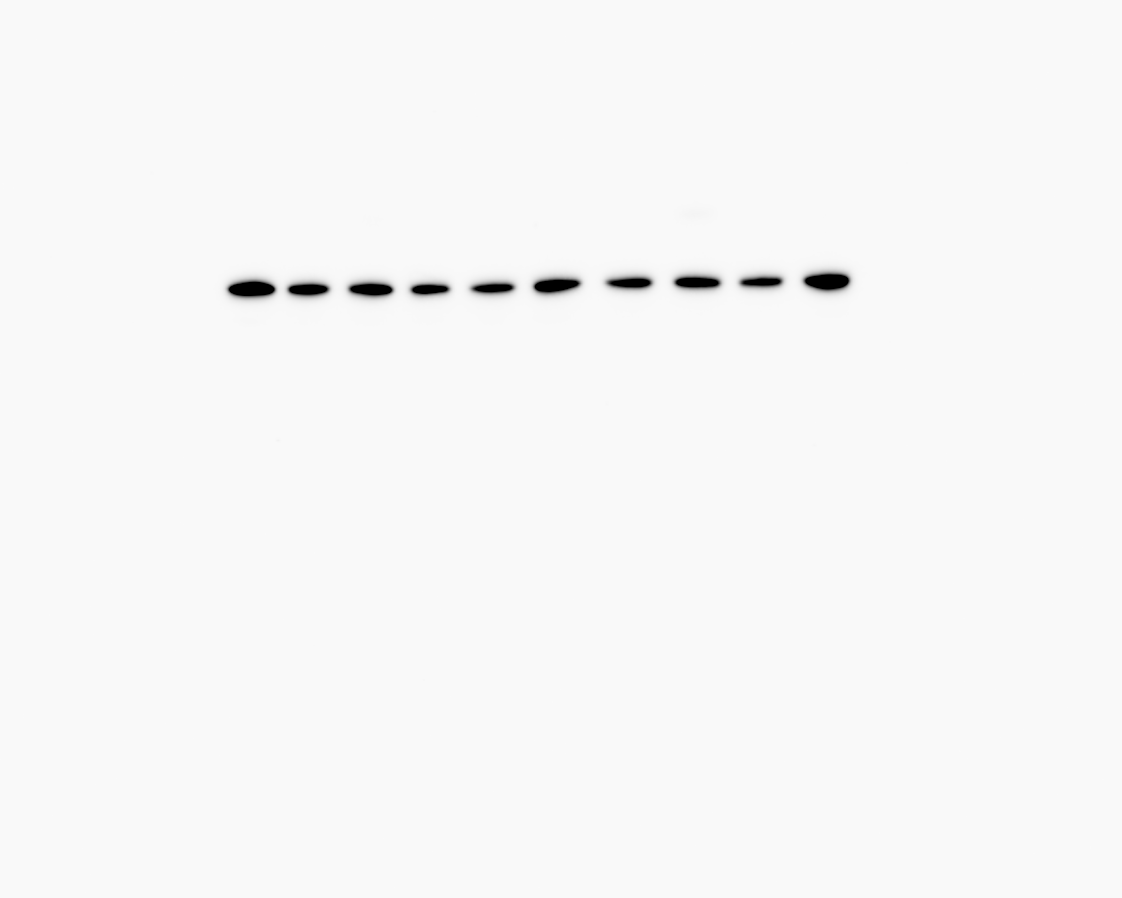

Supplement: S3 Dataset — (ZIP) [file ppat.1011597.s004.zip › S3_Dataset/Grabowski_et_al__Raw_Western_Blots/S1-Appendix--Figure-E--Panel-a--Western-blot/Raw files/GAPDH.tif]

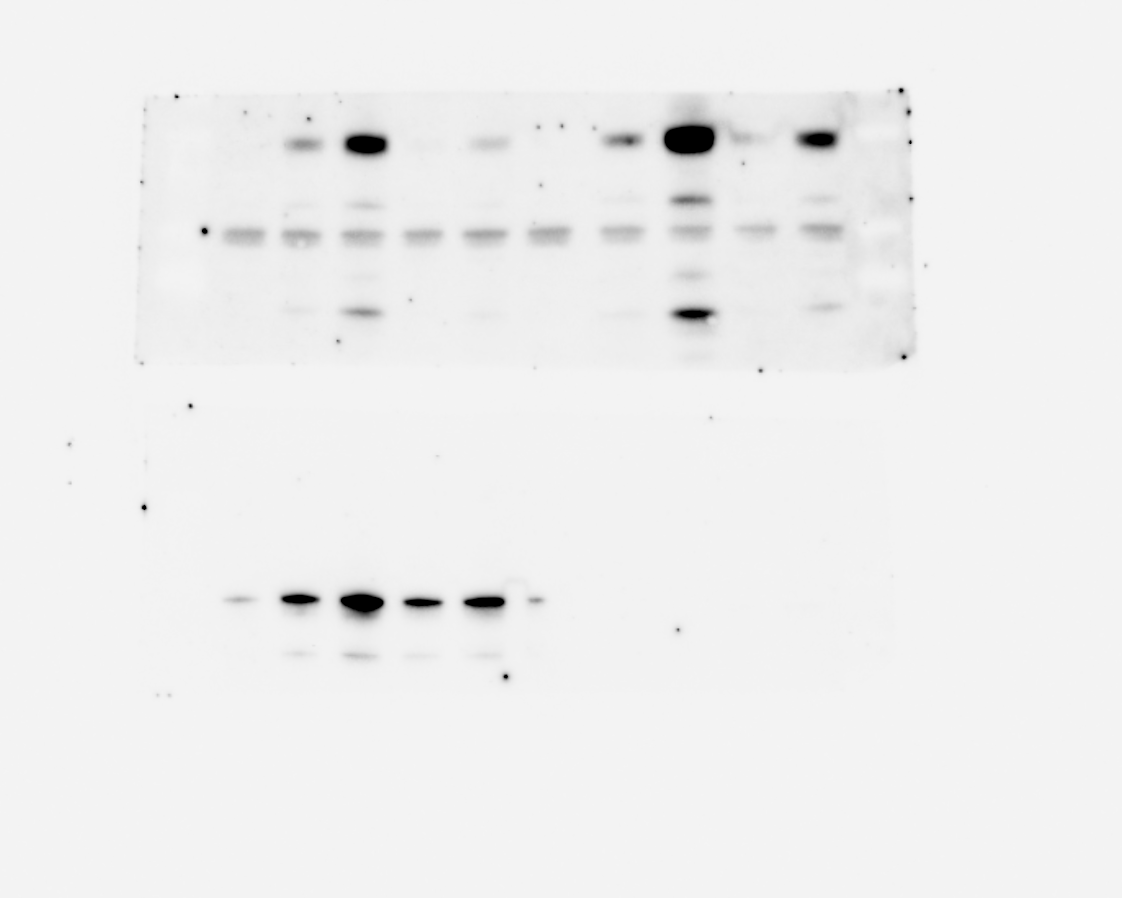

Supplement: S3 Dataset — (ZIP) [file ppat.1011597.s004.zip › S3_Dataset/Grabowski_et_al__Raw_Western_Blots/S1-Appendix--Figure-E--Panel-a--Western-blot/Raw files/RSV F.tif]

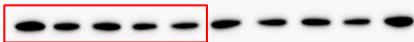

Supplement: S3 Dataset — (ZIP) [file ppat.1011597.s004.zip › S3_Dataset/Grabowski_et_al__Raw_Western_Blots/S1-Appendix--Figure-E--Panel-a--Western-blot/WB bands outlined/GAPDH.pdf]

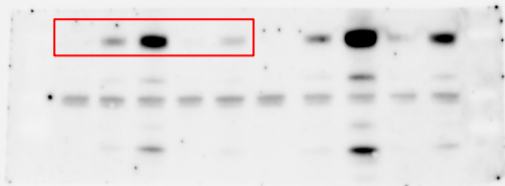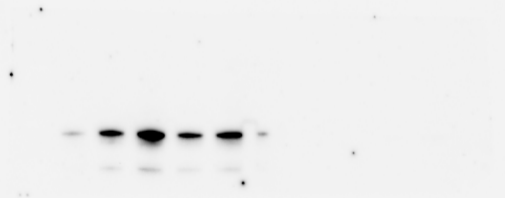

Supplement: S3 Dataset — (ZIP) [file ppat.1011597.s004.zip › S3_Dataset/Grabowski_et_al__Raw_Western_Blots/S1-Appendix--Figure-E--Panel-a--Western-blot/WB bands outlined/RSV F.pdf]

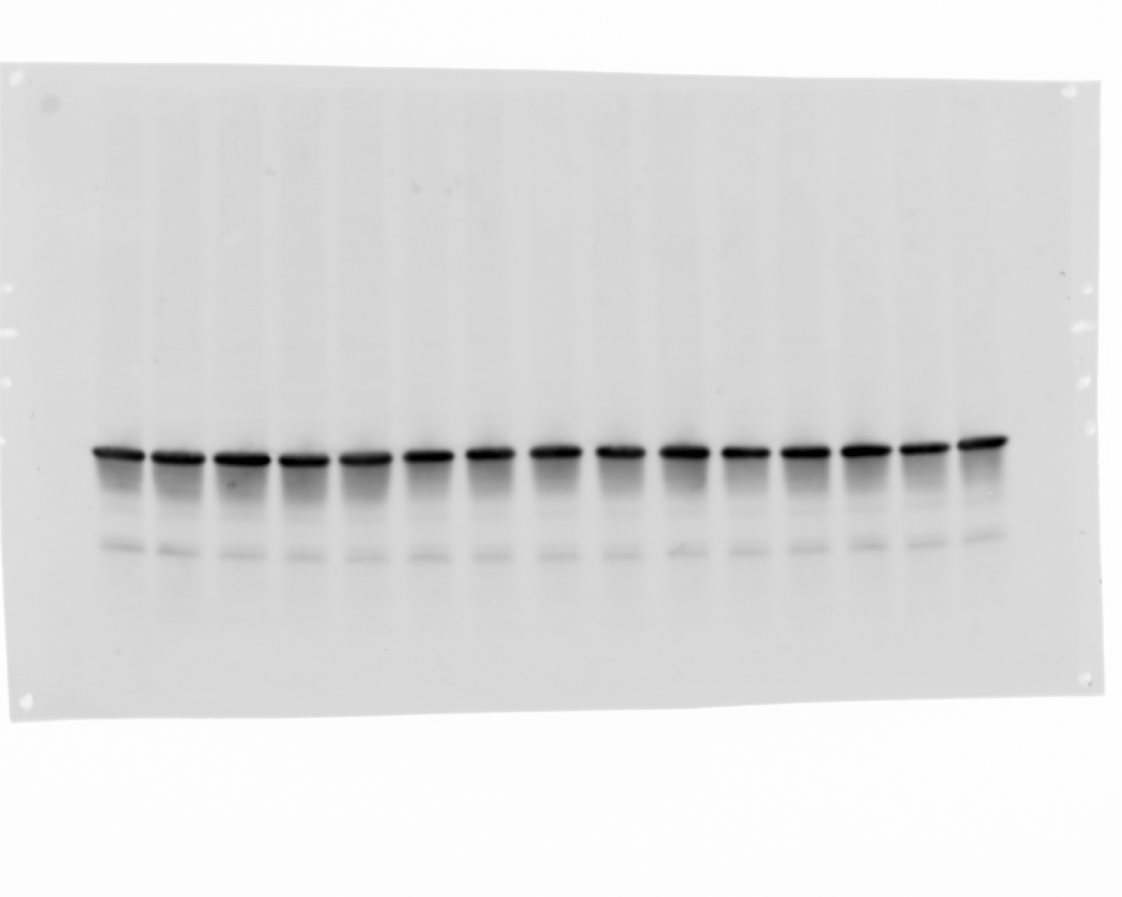

Supplement: S3 Dataset — (ZIP) [file ppat.1011597.s004.zip › S3_Dataset/Grabowski_et_al__Raw_Western_Blots/S1-Appendix--Figure-D--Panel-c--Western-blot/Raw files/GAPDH.tif]

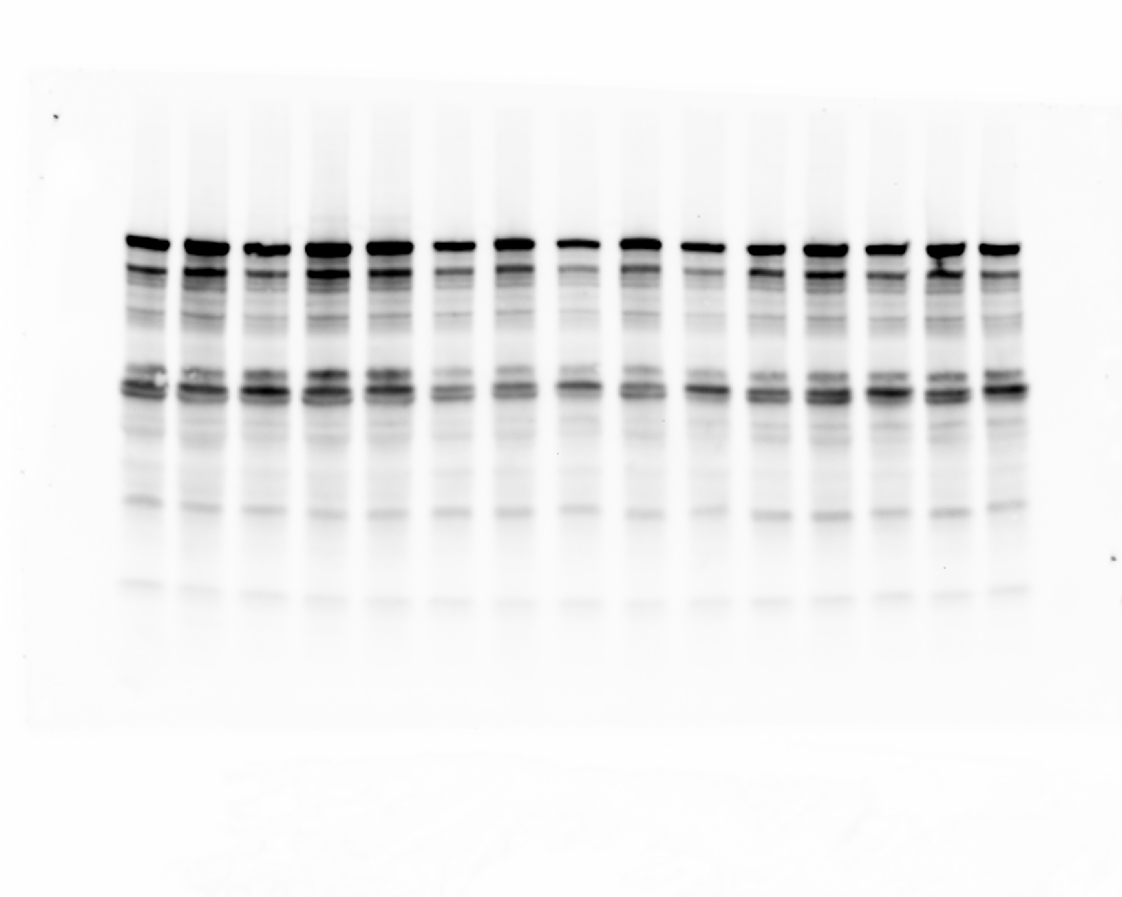

Supplement: S3 Dataset — (ZIP) [file ppat.1011597.s004.zip › S3_Dataset/Grabowski_et_al__Raw_Western_Blots/S1-Appendix--Figure-D--Panel-c--Western-blot/Raw files/IRF3.tif]

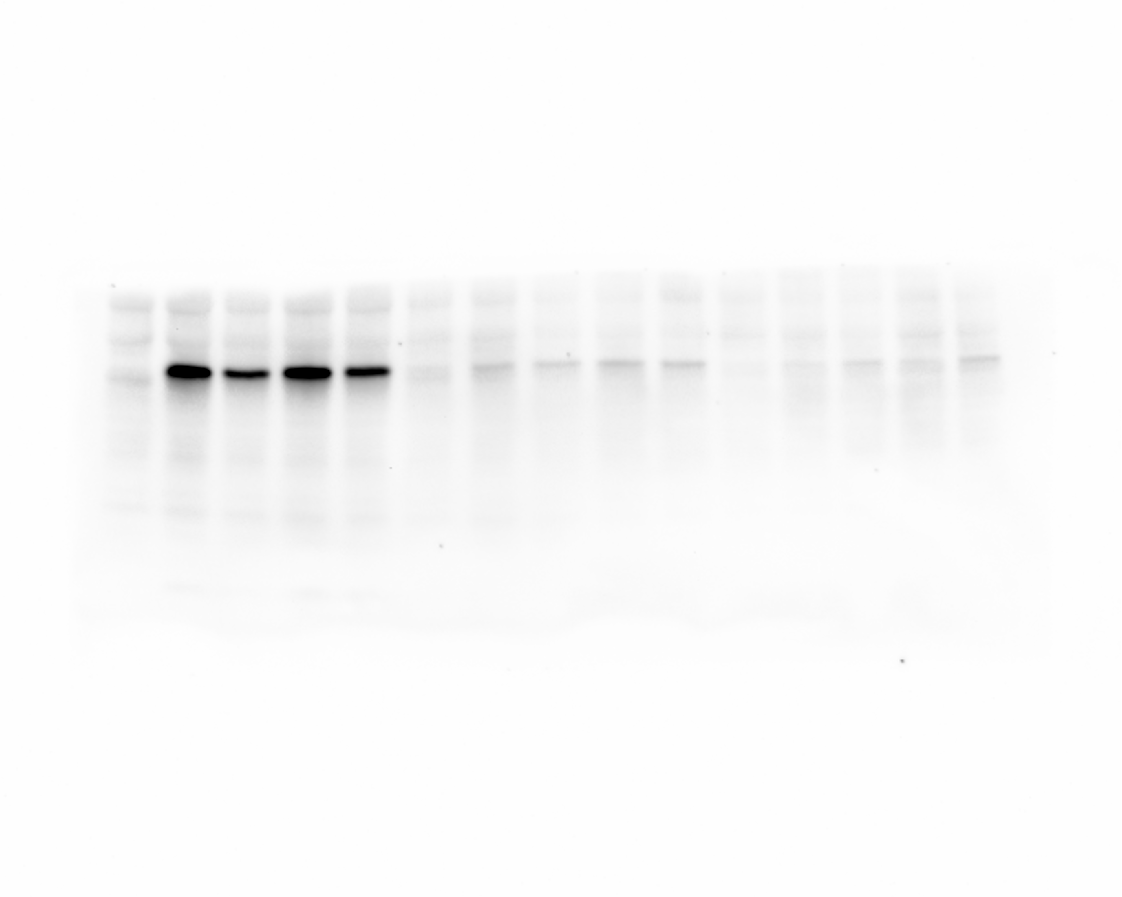

Supplement: S3 Dataset — (ZIP) [file ppat.1011597.s004.zip › S3_Dataset/Grabowski_et_al__Raw_Western_Blots/S1-Appendix--Figure-D--Panel-c--Western-blot/Raw files/OAS1.tif]

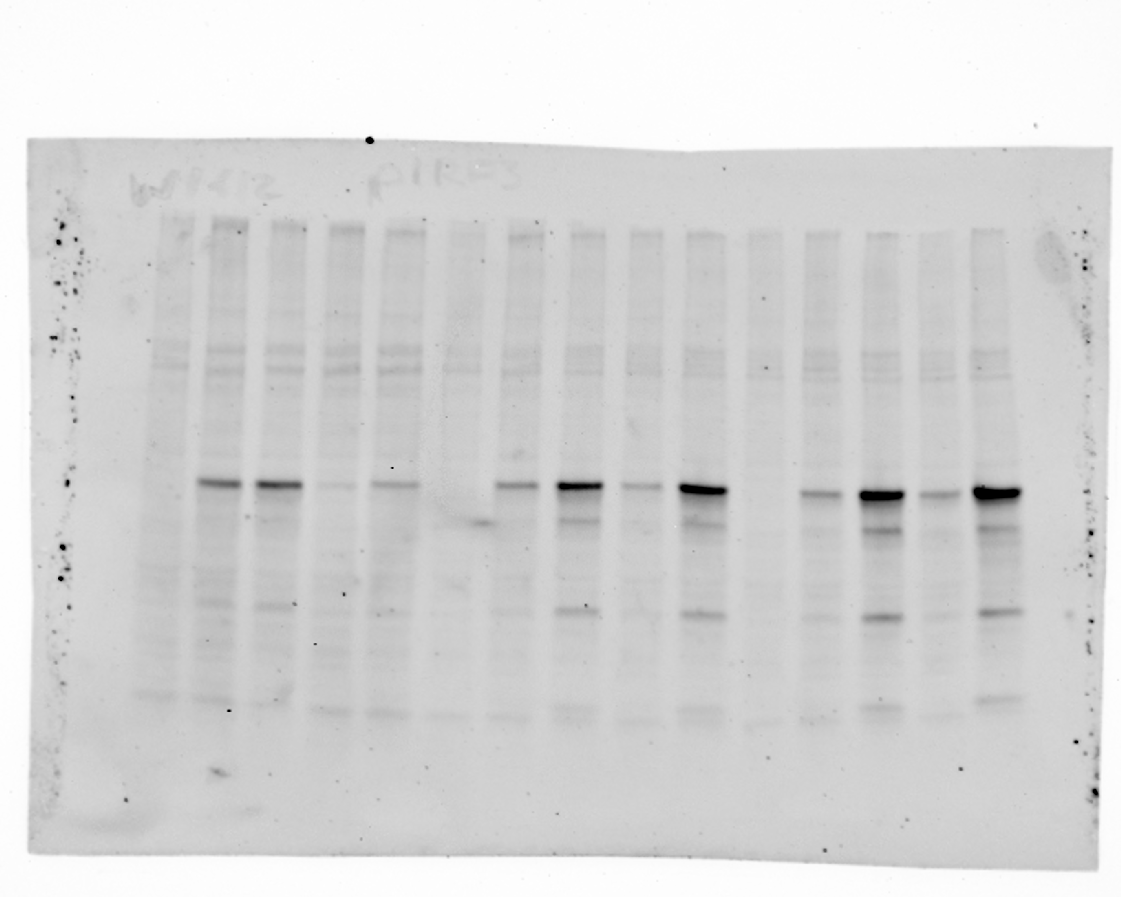

Supplement: S3 Dataset — (ZIP) [file ppat.1011597.s004.zip › S3_Dataset/Grabowski_et_al__Raw_Western_Blots/S1-Appendix--Figure-D--Panel-c--Western-blot/Raw files/p-IRF3.tif]

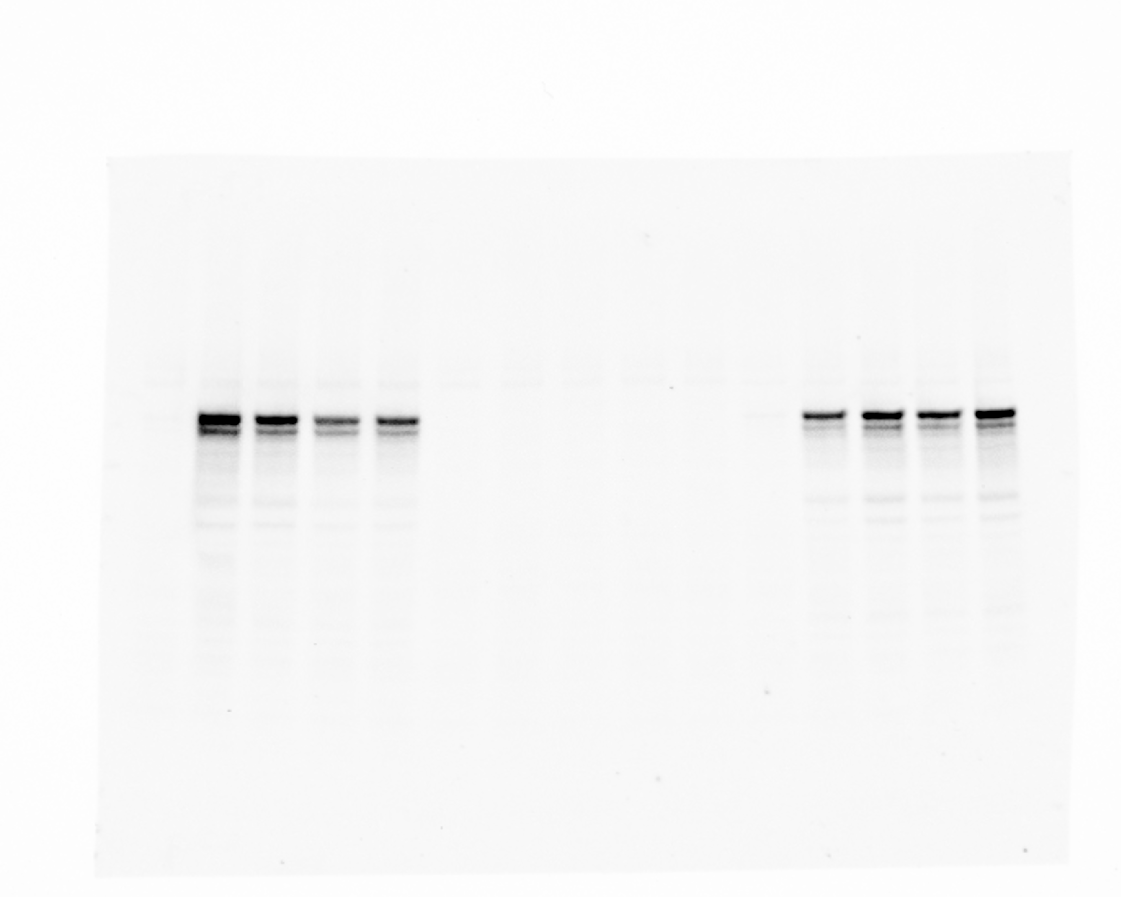

Supplement: S3 Dataset — (ZIP) [file ppat.1011597.s004.zip › S3_Dataset/Grabowski_et_al__Raw_Western_Blots/S1-Appendix--Figure-D--Panel-c--Western-blot/Raw files/p-STAT1.tif]

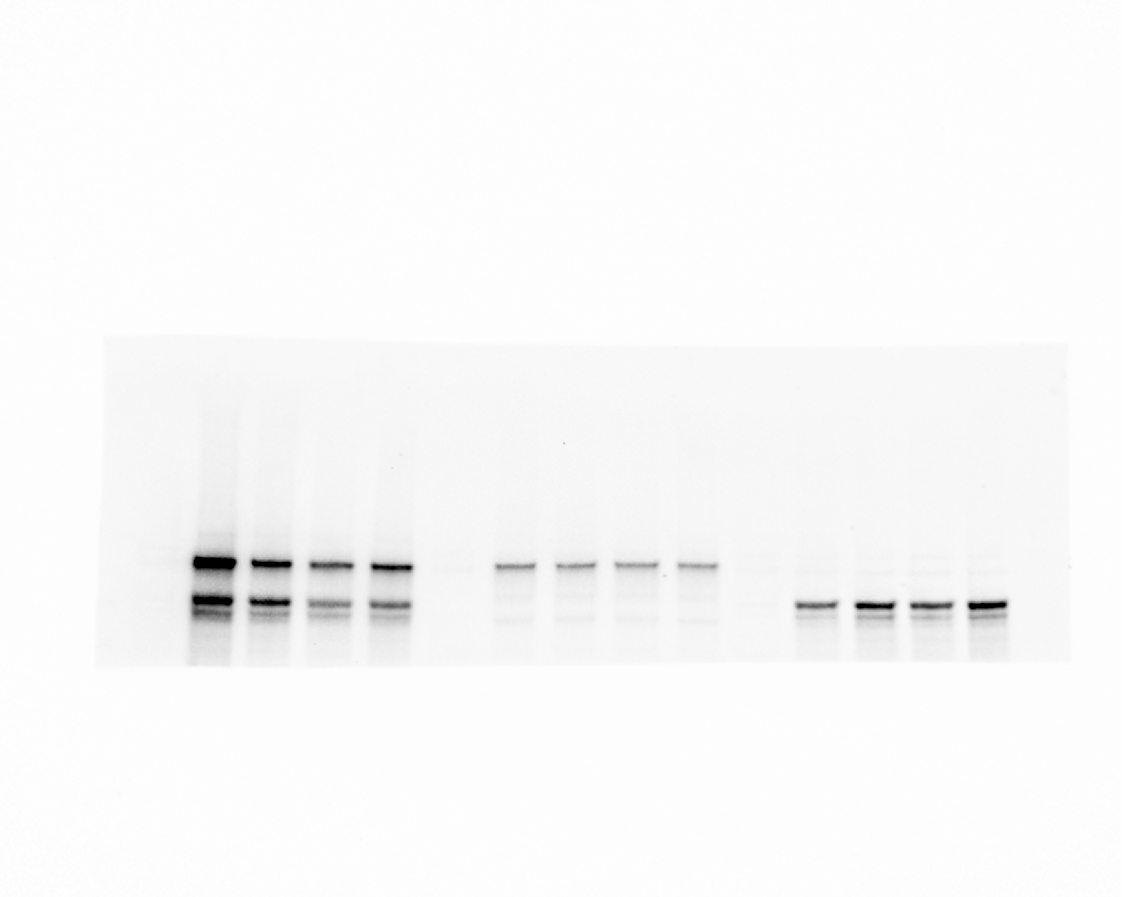

Supplement: S3 Dataset — (ZIP) [file ppat.1011597.s004.zip › S3_Dataset/Grabowski_et_al__Raw_Western_Blots/S1-Appendix--Figure-D--Panel-c--Western-blot/Raw files/p-STAT2.tif]

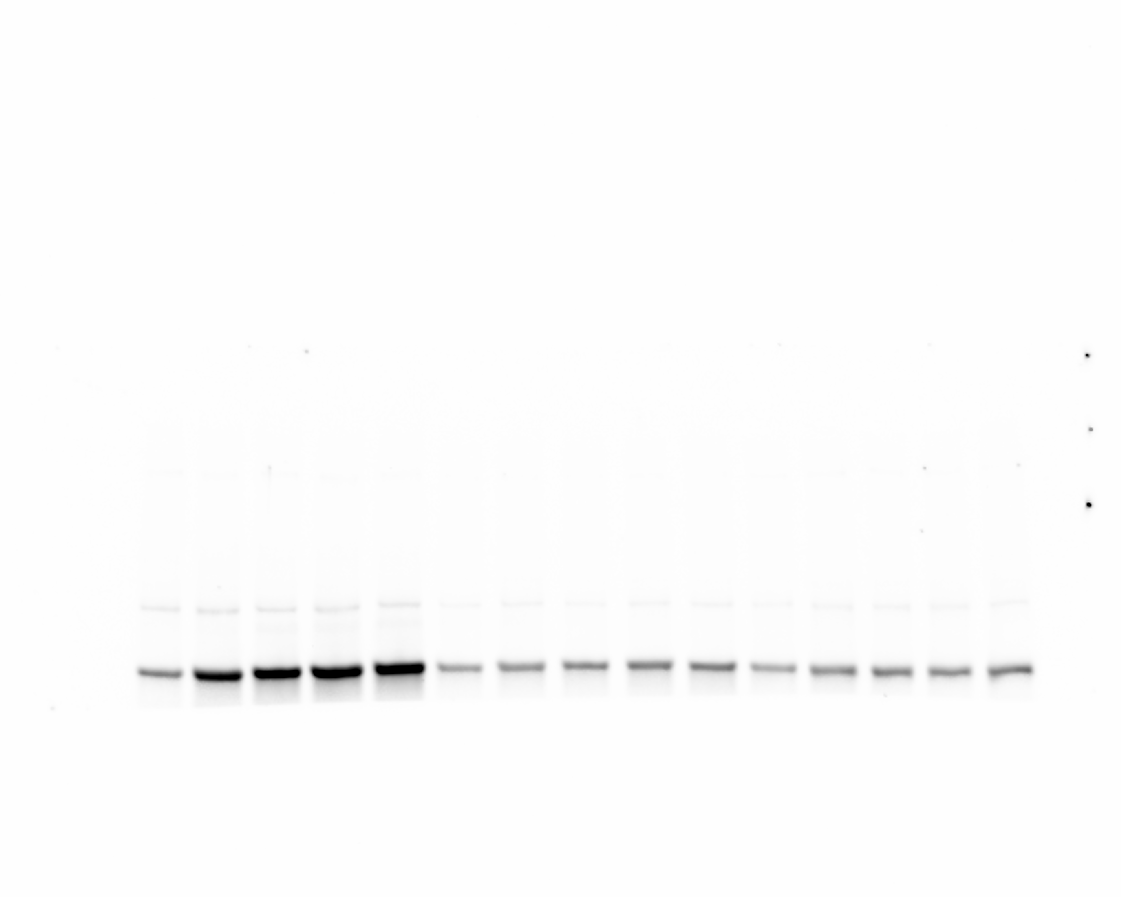

Supplement: S3 Dataset — (ZIP) [file ppat.1011597.s004.zip › S3_Dataset/Grabowski_et_al__Raw_Western_Blots/S1-Appendix--Figure-D--Panel-c--Western-blot/Raw files/PKR.tif]

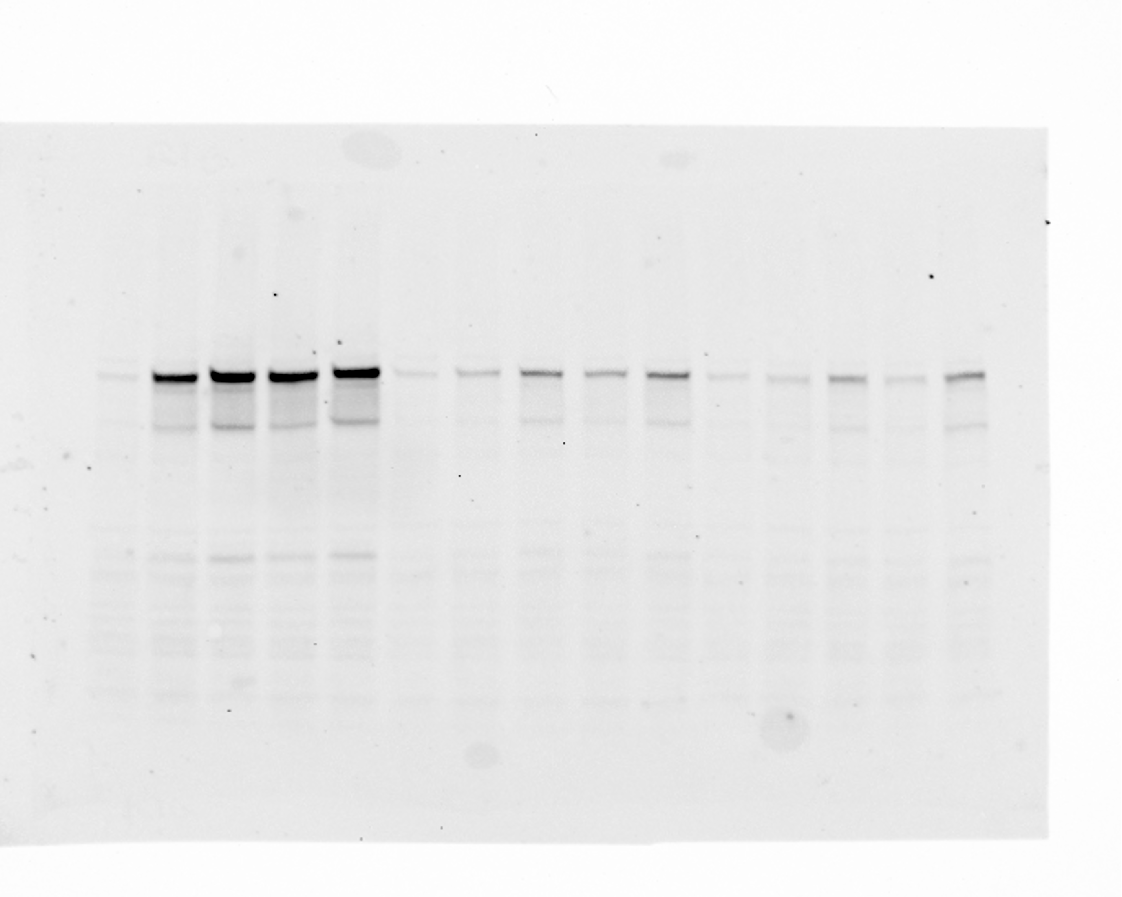

Supplement: S3 Dataset — (ZIP) [file ppat.1011597.s004.zip › S3_Dataset/Grabowski_et_al__Raw_Western_Blots/S1-Appendix--Figure-D--Panel-c--Western-blot/Raw files/RIG-I.tif]

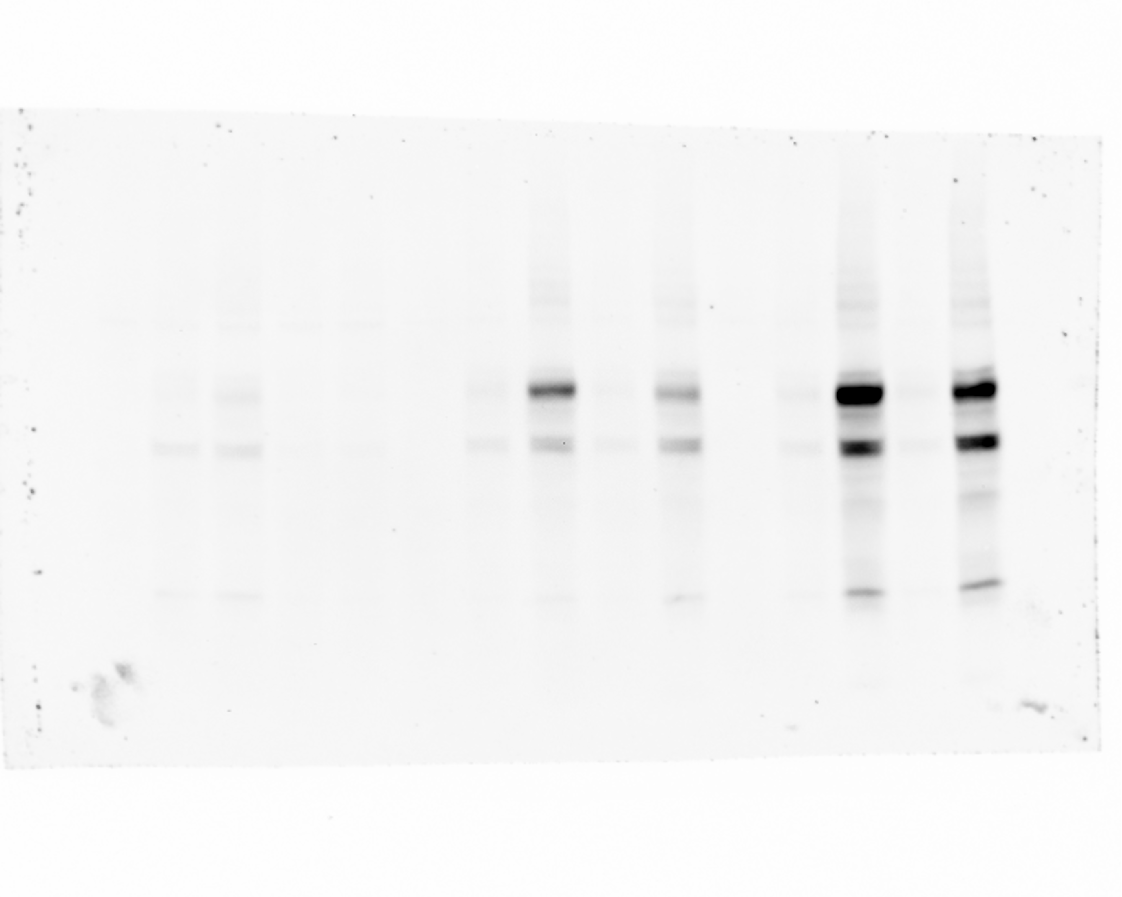

Supplement: S3 Dataset — (ZIP) [file ppat.1011597.s004.zip › S3_Dataset/Grabowski_et_al__Raw_Western_Blots/S1-Appendix--Figure-D--Panel-c--Western-blot/Raw files/RSV F.tif]

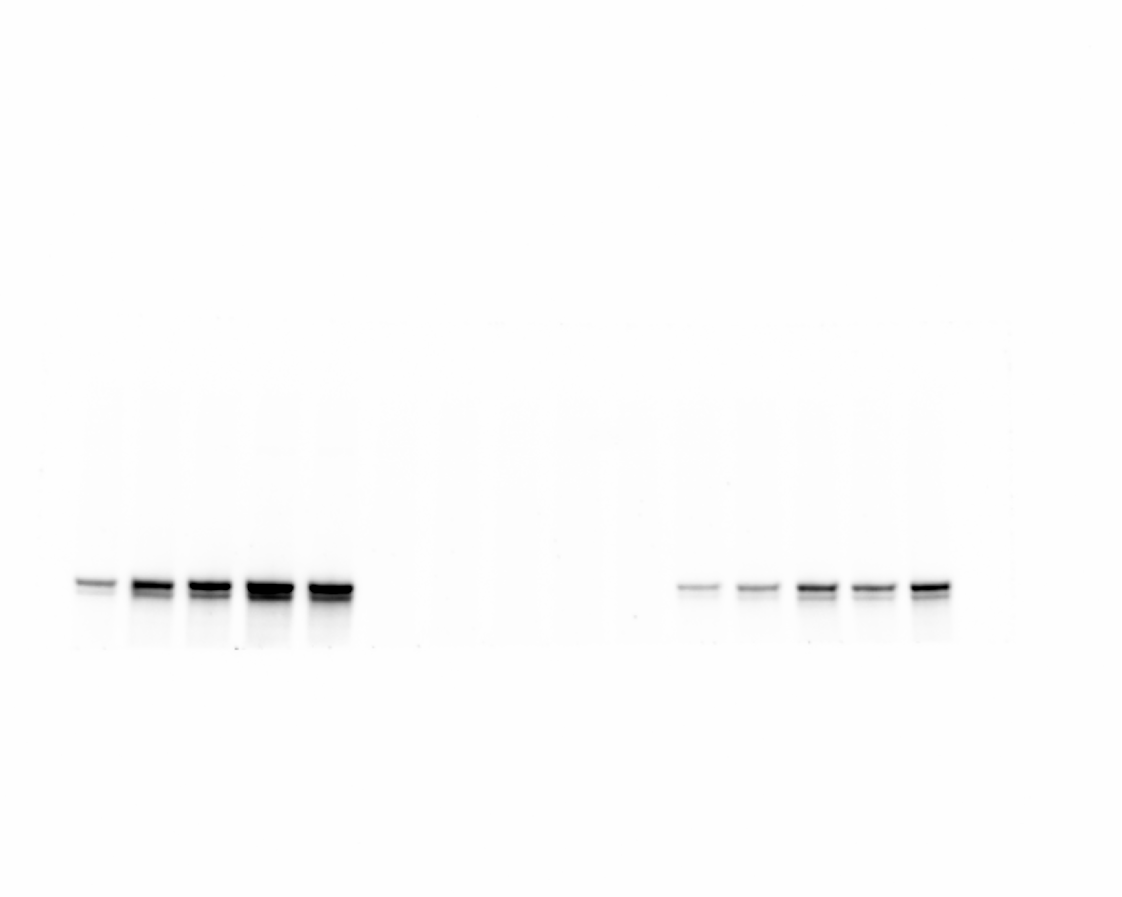

Supplement: S3 Dataset — (ZIP) [file ppat.1011597.s004.zip › S3_Dataset/Grabowski_et_al__Raw_Western_Blots/S1-Appendix--Figure-D--Panel-c--Western-blot/Raw files/STAT1.tif]

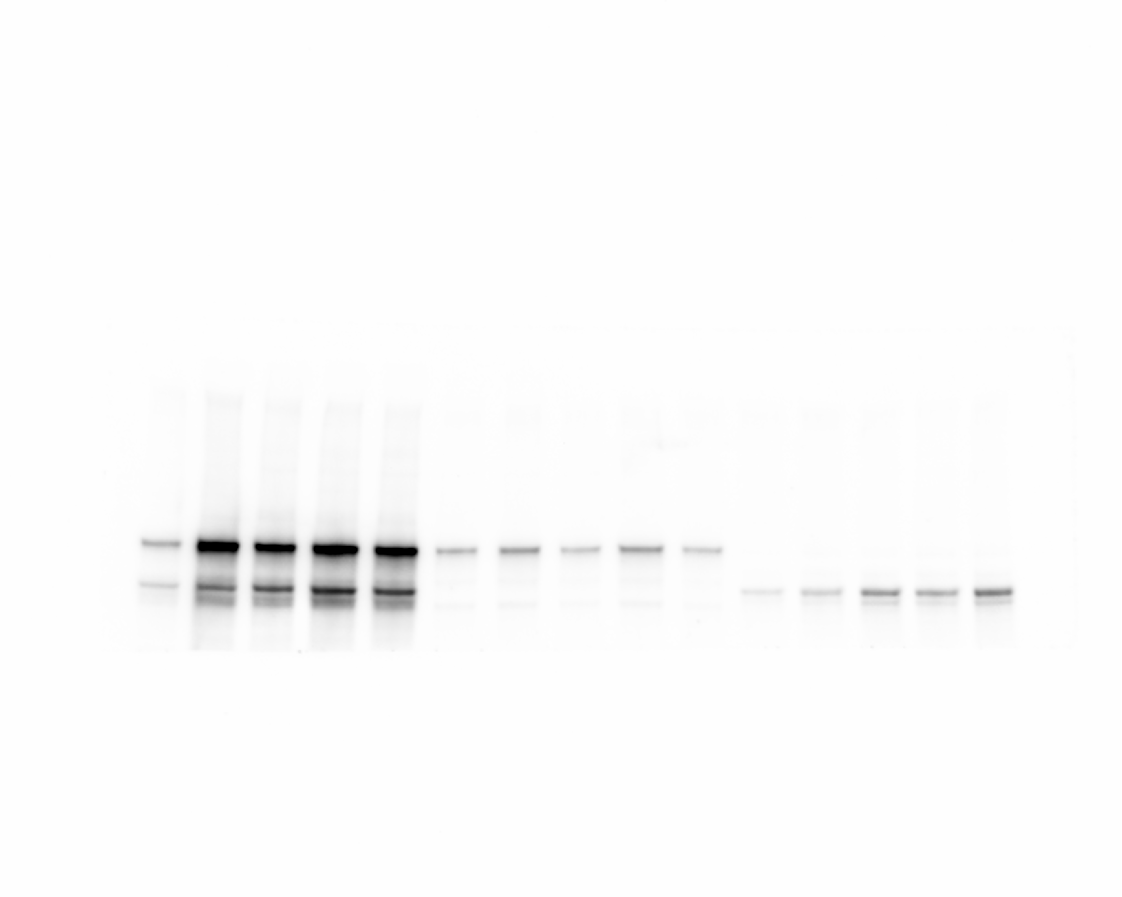

Supplement: S3 Dataset — (ZIP) [file ppat.1011597.s004.zip › S3_Dataset/Grabowski_et_al__Raw_Western_Blots/S1-Appendix--Figure-D--Panel-c--Western-blot/Raw files/STAT2.tif]

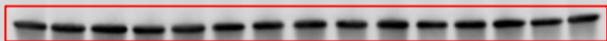

Supplement: S3 Dataset — (ZIP) [file ppat.1011597.s004.zip › S3_Dataset/Grabowski_et_al__Raw_Western_Blots/S1-Appendix--Figure-D--Panel-c--Western-blot/WB bands outlined/GAPDH.pdf]

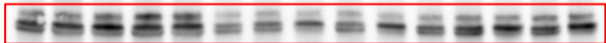

Supplement: S3 Dataset — (ZIP) [file ppat.1011597.s004.zip › S3_Dataset/Grabowski_et_al__Raw_Western_Blots/S1-Appendix--Figure-D--Panel-c--Western-blot/WB bands outlined/IRF3.pdf]

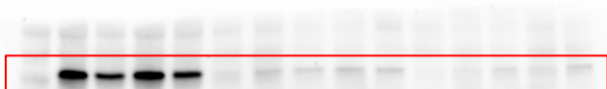

Supplement: S3 Dataset — (ZIP) [file ppat.1011597.s004.zip › S3_Dataset/Grabowski_et_al__Raw_Western_Blots/S1-Appendix--Figure-D--Panel-c--Western-blot/WB bands outlined/OAS1.pdf]

W1412

P1803

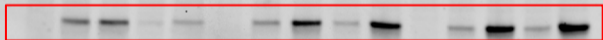

Supplement: S3 Dataset — (ZIP) [file ppat.1011597.s004.zip › S3_Dataset/Grabowski_et_al__Raw_Western_Blots/S1-Appendix--Figure-D--Panel-c--Western-blot/WB bands outlined/p-IRF3.pdf]

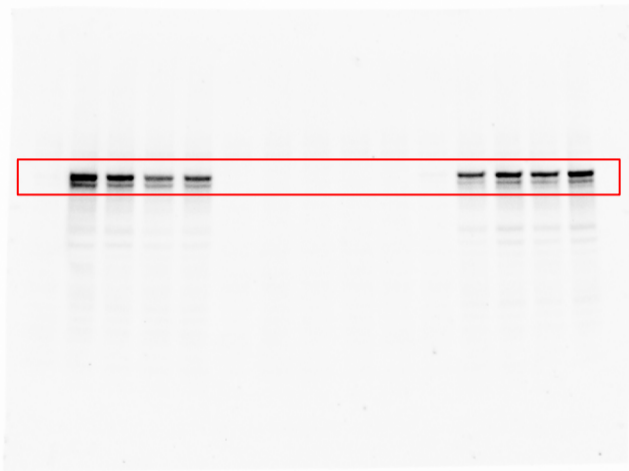

Supplement: S3 Dataset — (ZIP) [file ppat.1011597.s004.zip › S3_Dataset/Grabowski_et_al__Raw_Western_Blots/S1-Appendix--Figure-D--Panel-c--Western-blot/WB bands outlined/p-STAT1.pdf]

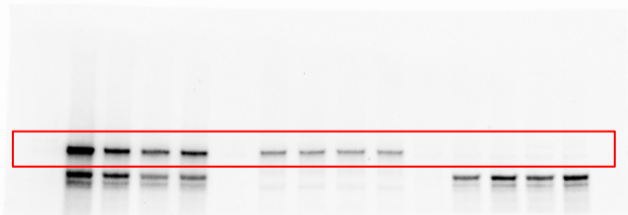

Supplement: S3 Dataset — (ZIP) [file ppat.1011597.s004.zip › S3_Dataset/Grabowski_et_al__Raw_Western_Blots/S1-Appendix--Figure-D--Panel-c--Western-blot/WB bands outlined/p-STAT2.pdf]

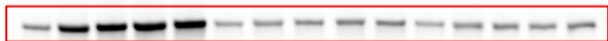

Supplement: S3 Dataset — (ZIP) [file ppat.1011597.s004.zip › S3_Dataset/Grabowski_et_al__Raw_Western_Blots/S1-Appendix--Figure-D--Panel-c--Western-blot/WB bands outlined/PKR.pdf]

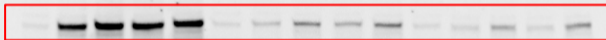

Supplement: S3 Dataset — (ZIP) [file ppat.1011597.s004.zip › S3_Dataset/Grabowski_et_al__Raw_Western_Blots/S1-Appendix--Figure-D--Panel-c--Western-blot/WB bands outlined/RIG-I.pdf]

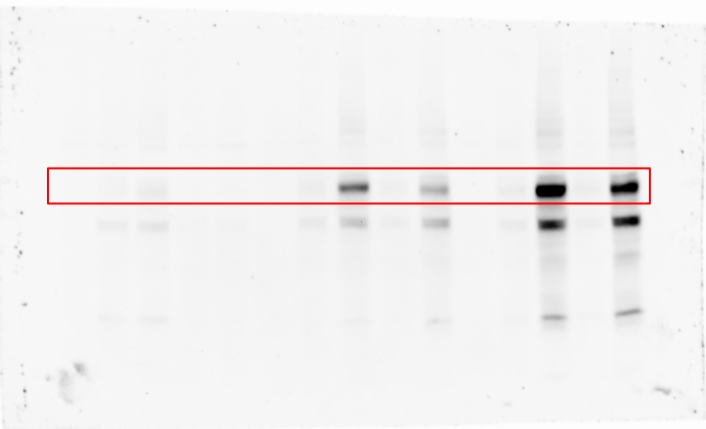

Supplement: S3 Dataset — (ZIP) [file ppat.1011597.s004.zip › S3_Dataset/Grabowski_et_al__Raw_Western_Blots/S1-Appendix--Figure-D--Panel-c--Western-blot/WB bands outlined/RSV F.pdf]

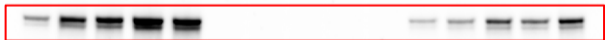

Supplement: S3 Dataset — (ZIP) [file ppat.1011597.s004.zip › S3_Dataset/Grabowski_et_al__Raw_Western_Blots/S1-Appendix--Figure-D--Panel-c--Western-blot/WB bands outlined/STAT1.pdf]

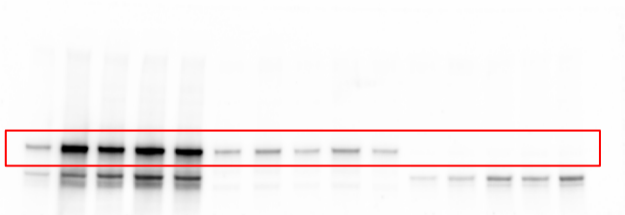

Supplement: S3 Dataset — (ZIP) [file ppat.1011597.s004.zip › S3_Dataset/Grabowski_et_al__Raw_Western_Blots/S1-Appendix--Figure-D--Panel-c--Western-blot/WB bands outlined/STAT2.pdf]

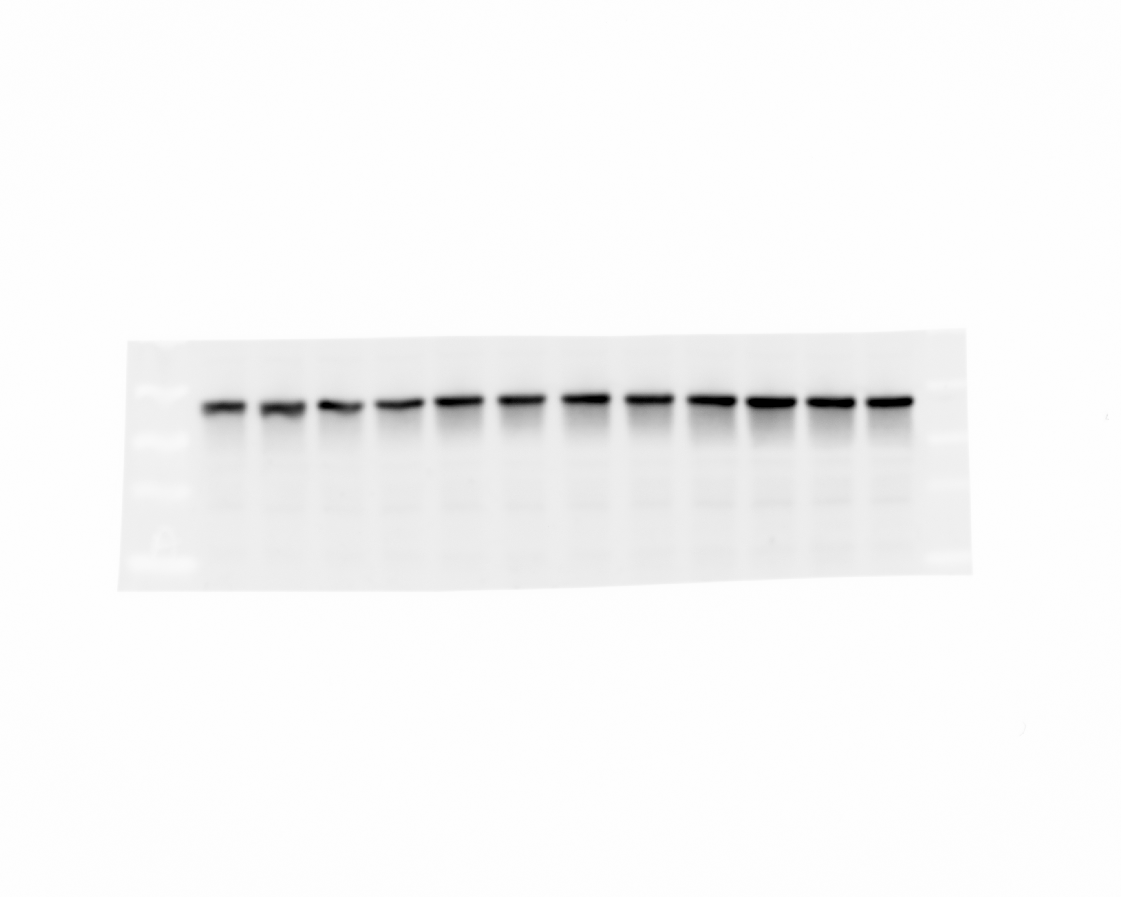

Supplement: S3 Dataset — (ZIP) [file ppat.1011597.s004.zip › S3_Dataset/Grabowski_et_al__Raw_Western_Blots/S1-Appendix--Figure-C--Panel-e--Western-blot/Raw files/GAPDH.tif]

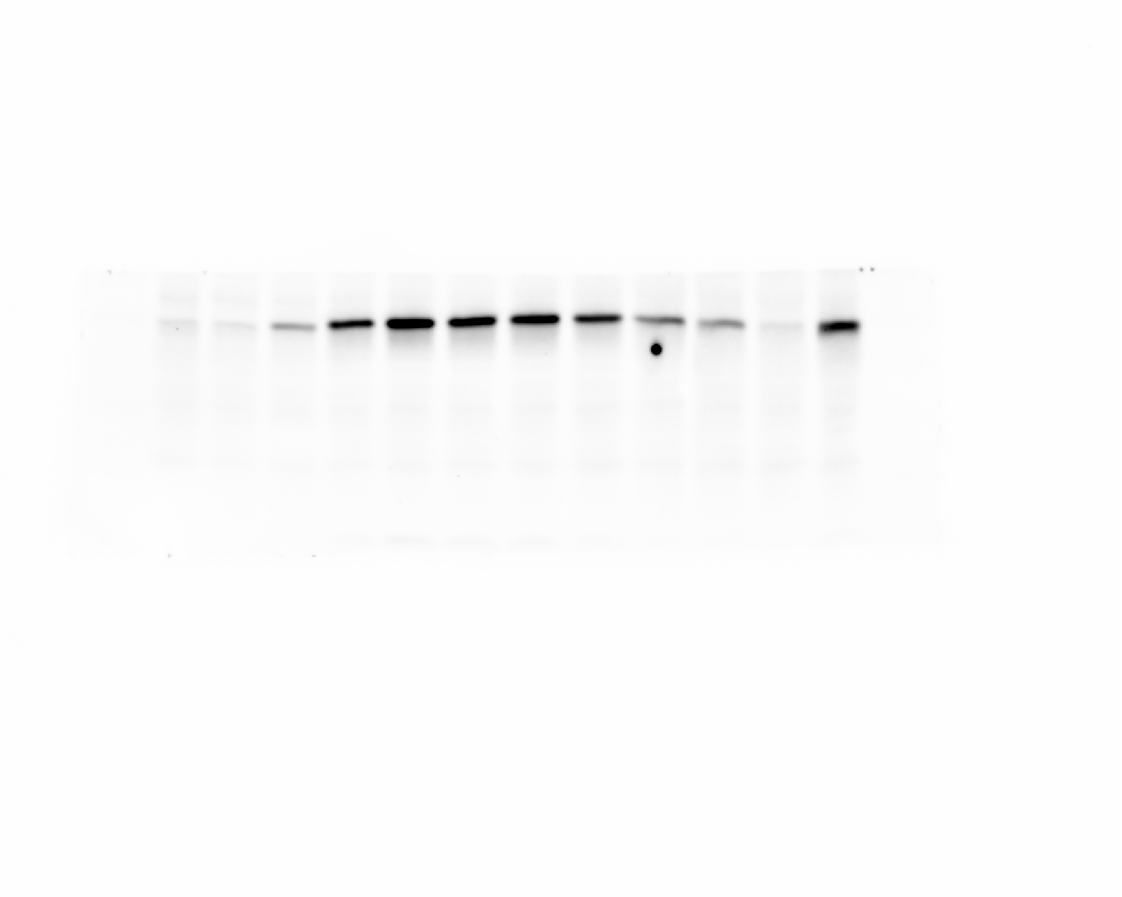

Supplement: S3 Dataset — (ZIP) [file ppat.1011597.s004.zip › S3_Dataset/Grabowski_et_al__Raw_Western_Blots/S1-Appendix--Figure-C--Panel-e--Western-blot/Raw files/OAS1.tif]

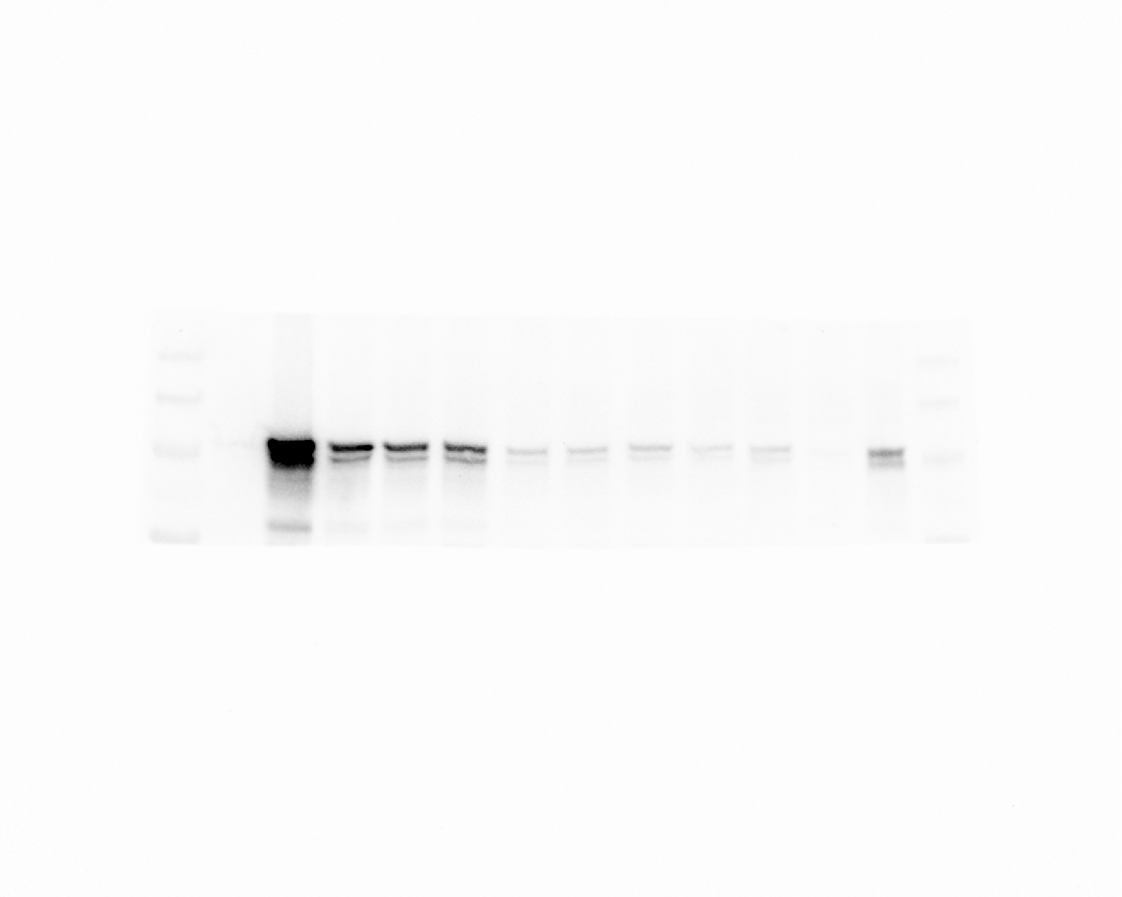

Supplement: S3 Dataset — (ZIP) [file ppat.1011597.s004.zip › S3_Dataset/Grabowski_et_al__Raw_Western_Blots/S1-Appendix--Figure-C--Panel-e--Western-blot/Raw files/p-STAT1.tif]

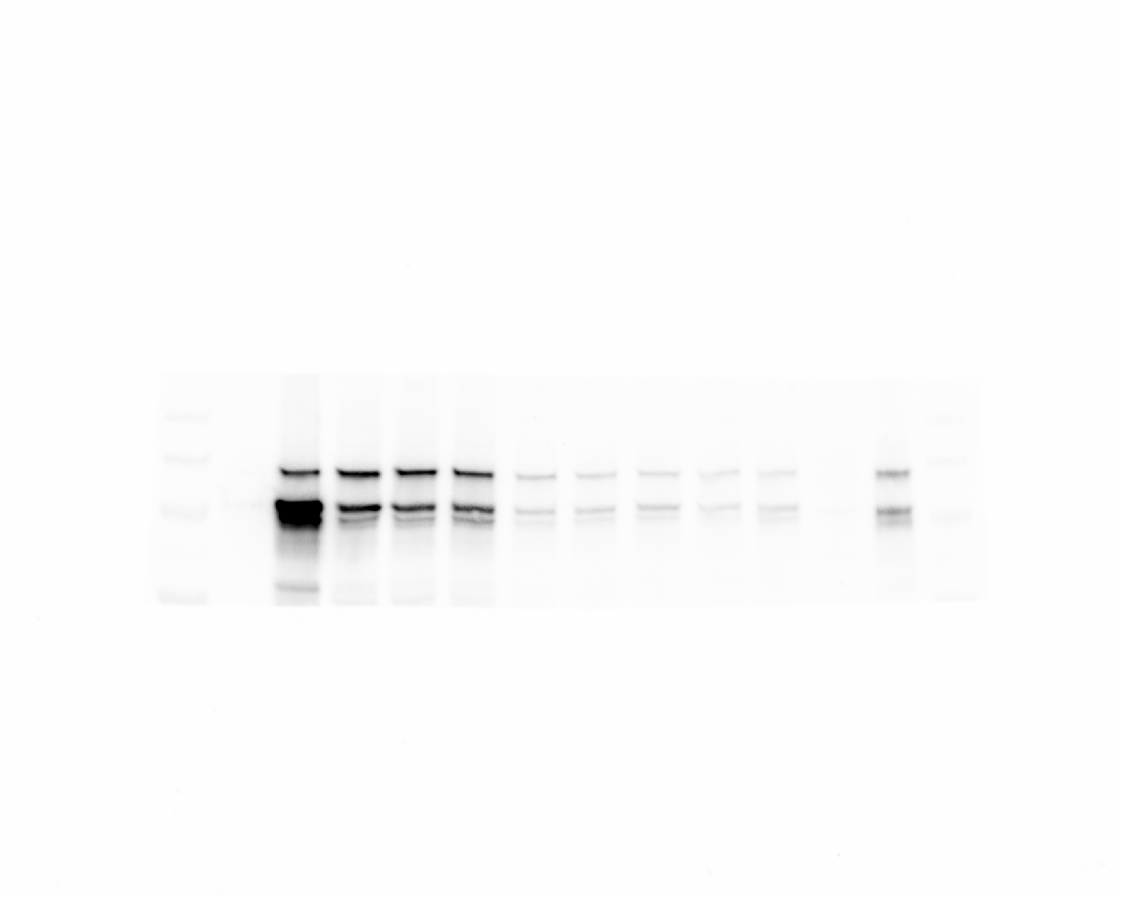

Supplement: S3 Dataset — (ZIP) [file ppat.1011597.s004.zip › S3_Dataset/Grabowski_et_al__Raw_Western_Blots/S1-Appendix--Figure-C--Panel-e--Western-blot/Raw files/p-STAT2.tif]

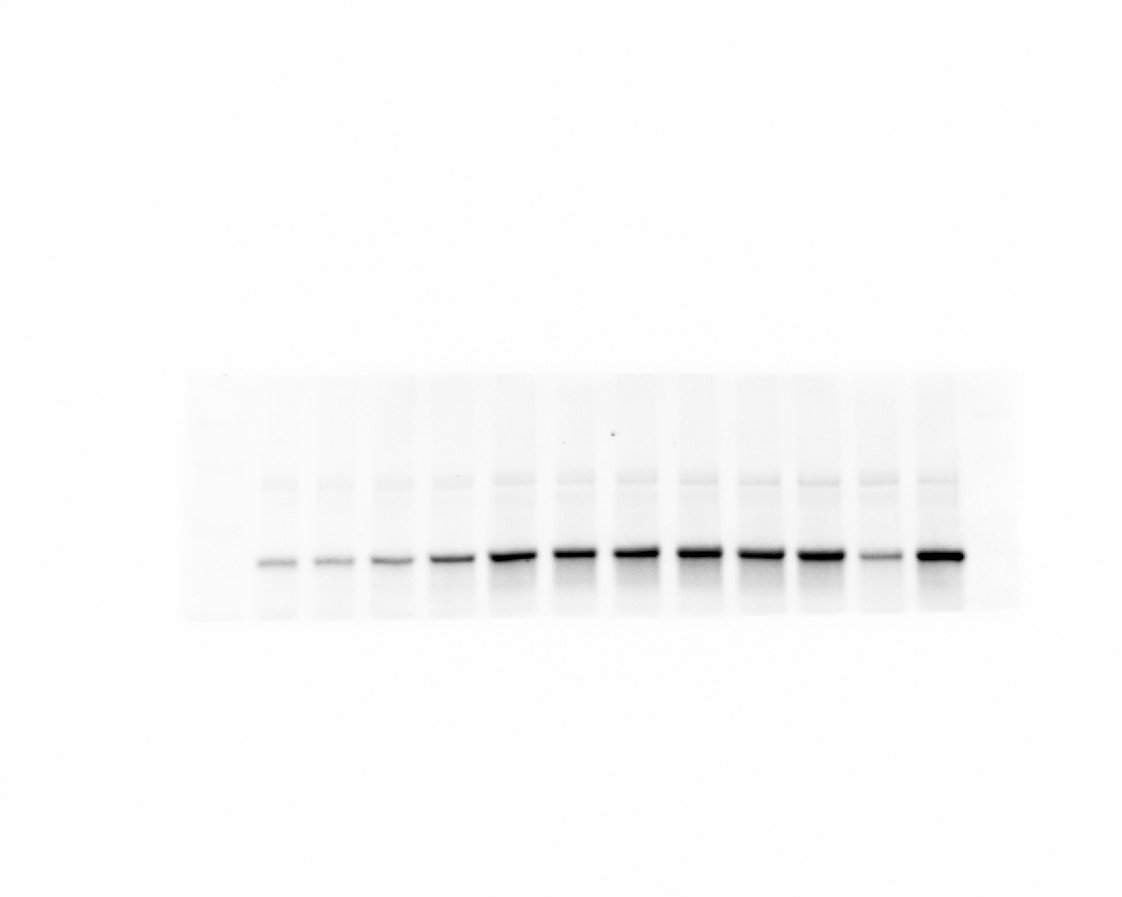

Supplement: S3 Dataset — (ZIP) [file ppat.1011597.s004.zip › S3_Dataset/Grabowski_et_al__Raw_Western_Blots/S1-Appendix--Figure-C--Panel-e--Western-blot/Raw files/PKR.tif]

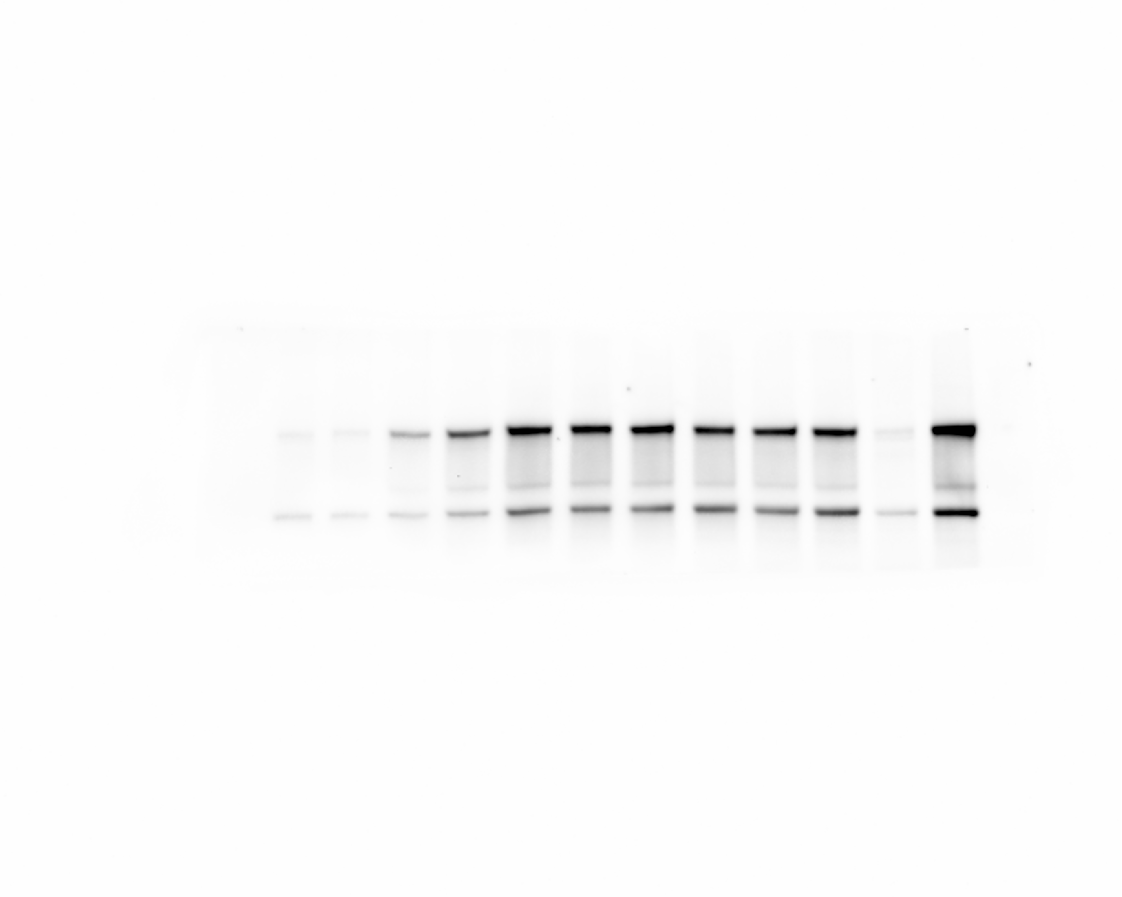

Supplement: S3 Dataset — (ZIP) [file ppat.1011597.s004.zip › S3_Dataset/Grabowski_et_al__Raw_Western_Blots/S1-Appendix--Figure-C--Panel-e--Western-blot/Raw files/RIG-I.tif]

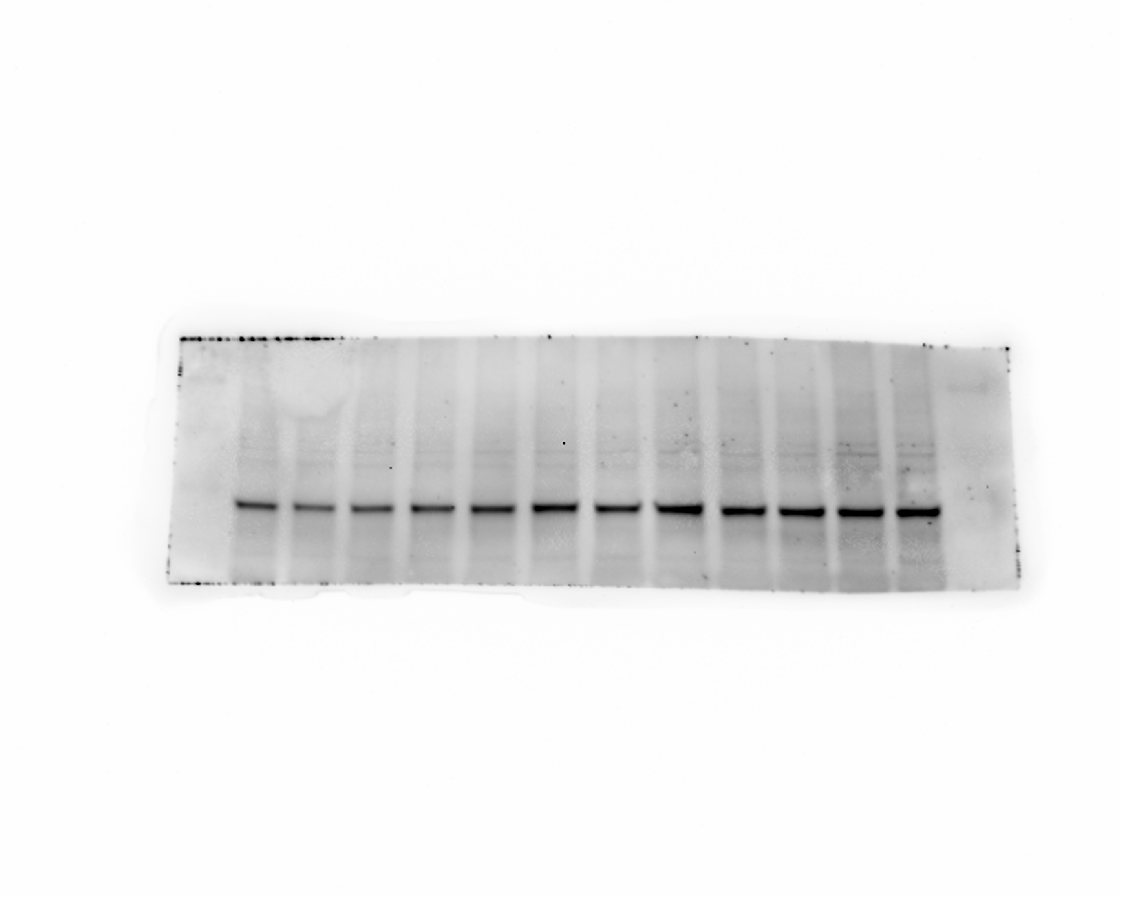

Supplement: S3 Dataset — (ZIP) [file ppat.1011597.s004.zip › S3_Dataset/Grabowski_et_al__Raw_Western_Blots/S1-Appendix--Figure-C--Panel-e--Western-blot/Raw files/RNase L.tif]

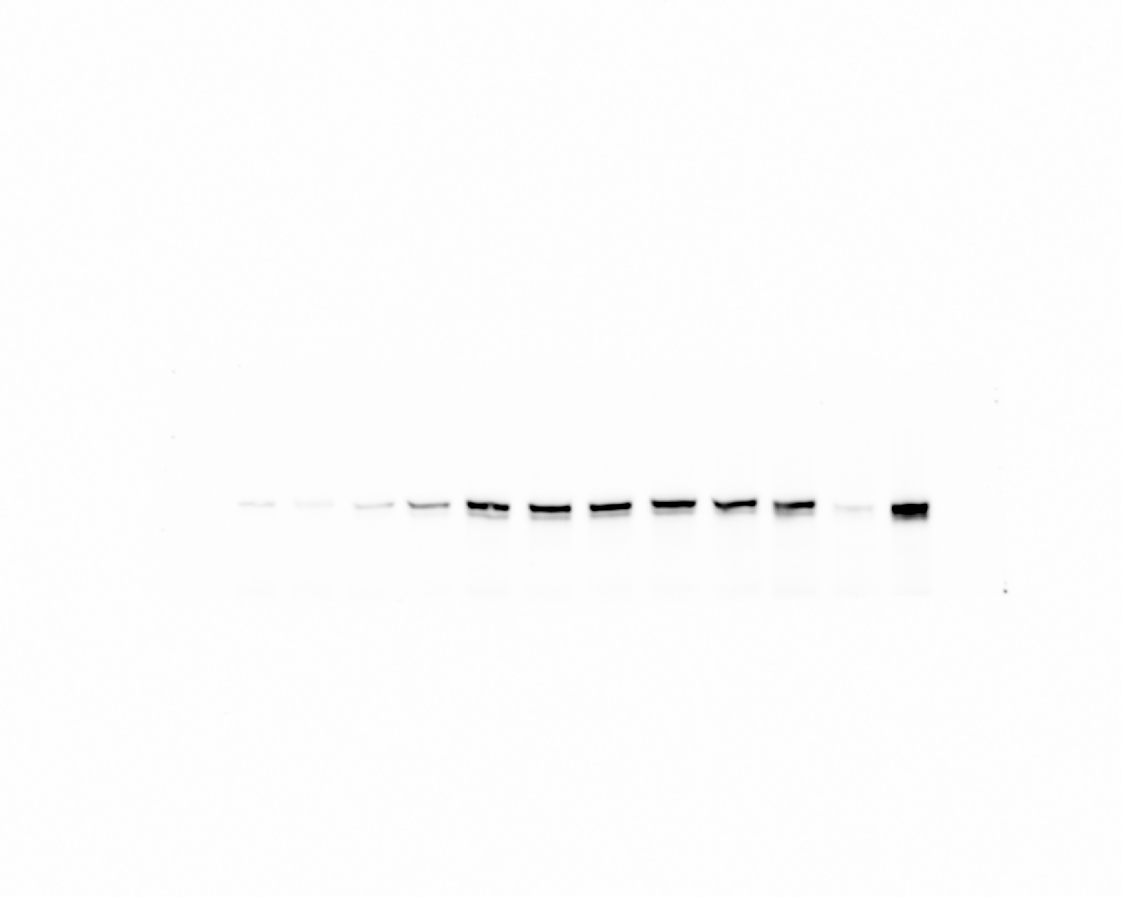

Supplement: S3 Dataset — (ZIP) [file ppat.1011597.s004.zip › S3_Dataset/Grabowski_et_al__Raw_Western_Blots/S1-Appendix--Figure-C--Panel-e--Western-blot/Raw files/STAT1.tif]

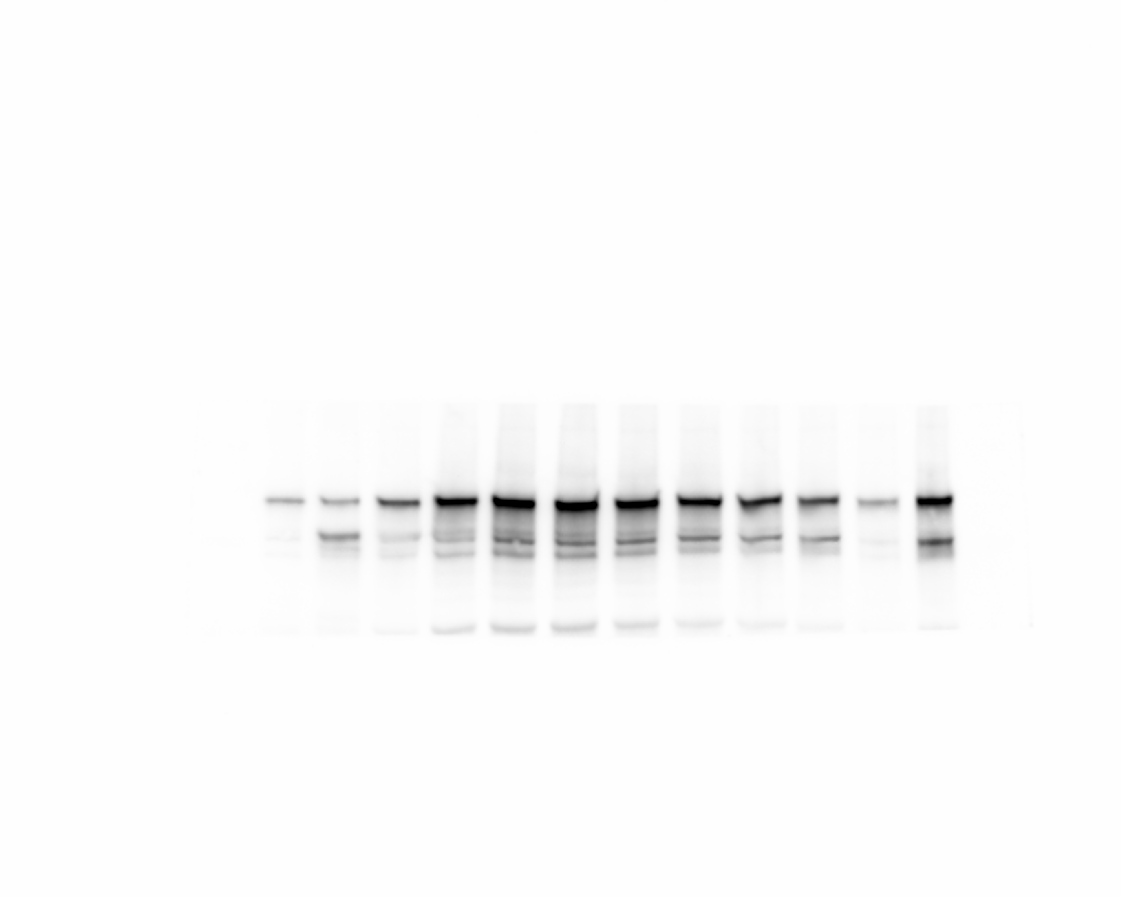

Supplement: S3 Dataset — (ZIP) [file ppat.1011597.s004.zip › S3_Dataset/Grabowski_et_al__Raw_Western_Blots/S1-Appendix--Figure-C--Panel-e--Western-blot/Raw files/STAT2.tif]

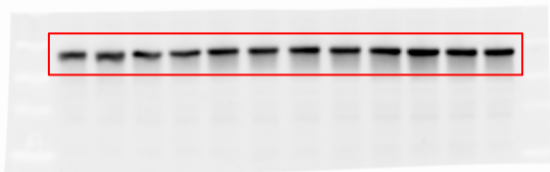

Supplement: S3 Dataset — (ZIP) [file ppat.1011597.s004.zip › S3_Dataset/Grabowski_et_al__Raw_Western_Blots/S1-Appendix--Figure-C--Panel-e--Western-blot/WB bands outlined/GAPDH.pdf]

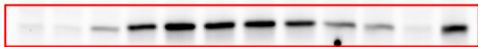

Supplement: S3 Dataset — (ZIP) [file ppat.1011597.s004.zip › S3_Dataset/Grabowski_et_al__Raw_Western_Blots/S1-Appendix--Figure-C--Panel-e--Western-blot/WB bands outlined/OAS1.pdf]

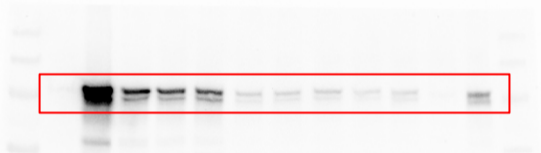

Supplement: S3 Dataset — (ZIP) [file ppat.1011597.s004.zip › S3_Dataset/Grabowski_et_al__Raw_Western_Blots/S1-Appendix--Figure-C--Panel-e--Western-blot/WB bands outlined/p-STAT1.pdf]

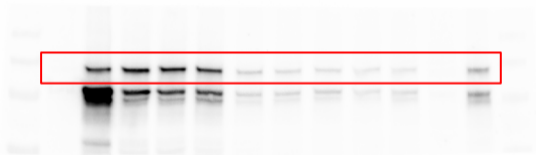

Supplement: S3 Dataset — (ZIP) [file ppat.1011597.s004.zip › S3_Dataset/Grabowski_et_al__Raw_Western_Blots/S1-Appendix--Figure-C--Panel-e--Western-blot/WB bands outlined/p-STAT2.pdf]

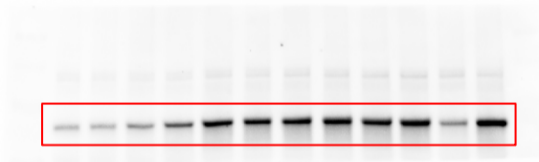

Supplement: S3 Dataset — (ZIP) [file ppat.1011597.s004.zip › S3_Dataset/Grabowski_et_al__Raw_Western_Blots/S1-Appendix--Figure-C--Panel-e--Western-blot/WB bands outlined/PKR.pdf]

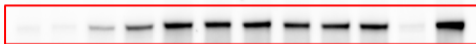

Supplement: S3 Dataset — (ZIP) [file ppat.1011597.s004.zip › S3_Dataset/Grabowski_et_al__Raw_Western_Blots/S1-Appendix--Figure-C--Panel-e--Western-blot/WB bands outlined/RIG-I.pdf]

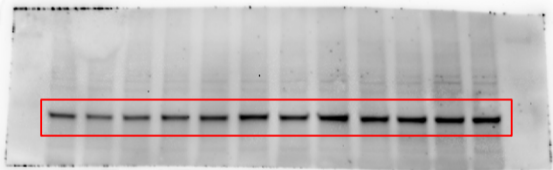

Supplement: S3 Dataset — (ZIP) [file ppat.1011597.s004.zip › S3_Dataset/Grabowski_et_al__Raw_Western_Blots/S1-Appendix--Figure-C--Panel-e--Western-blot/WB bands outlined/RNase L.pdf]

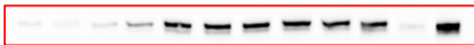

Supplement: S3 Dataset — (ZIP) [file ppat.1011597.s004.zip › S3_Dataset/Grabowski_et_al__Raw_Western_Blots/S1-Appendix--Figure-C--Panel-e--Western-blot/WB bands outlined/STAT1.pdf]

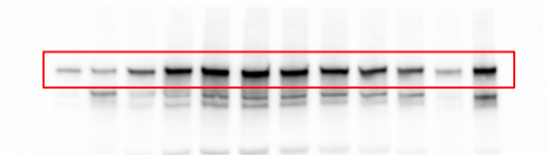

Supplement: S3 Dataset — (ZIP) [file ppat.1011597.s004.zip › S3_Dataset/Grabowski_et_al__Raw_Western_Blots/S1-Appendix--Figure-C--Panel-e--Western-blot/WB bands outlined/STAT2.pdf]

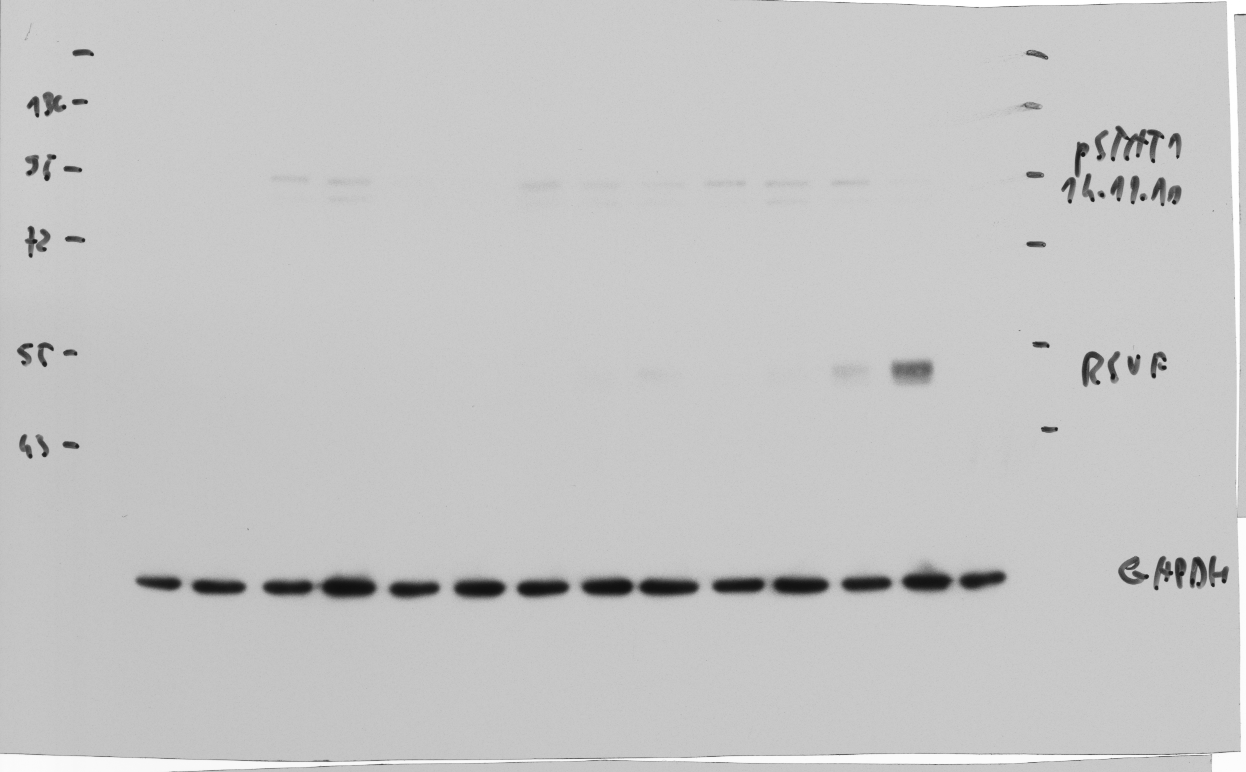

Supplement: S3 Dataset — (ZIP) [file ppat.1011597.s004.zip › S3_Dataset/Grabowski_et_al__Raw_Western_Blots/S1-Appendix--Figure-A--Panel-a--Western-blot/Raw files/GAPDH.tif]

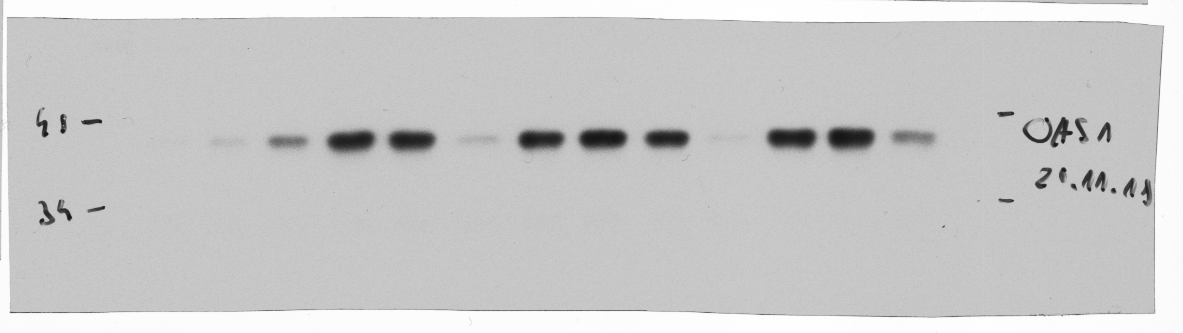

Supplement: S3 Dataset — (ZIP) [file ppat.1011597.s004.zip › S3_Dataset/Grabowski_et_al__Raw_Western_Blots/S1-Appendix--Figure-A--Panel-a--Western-blot/Raw files/OAS1.tif]

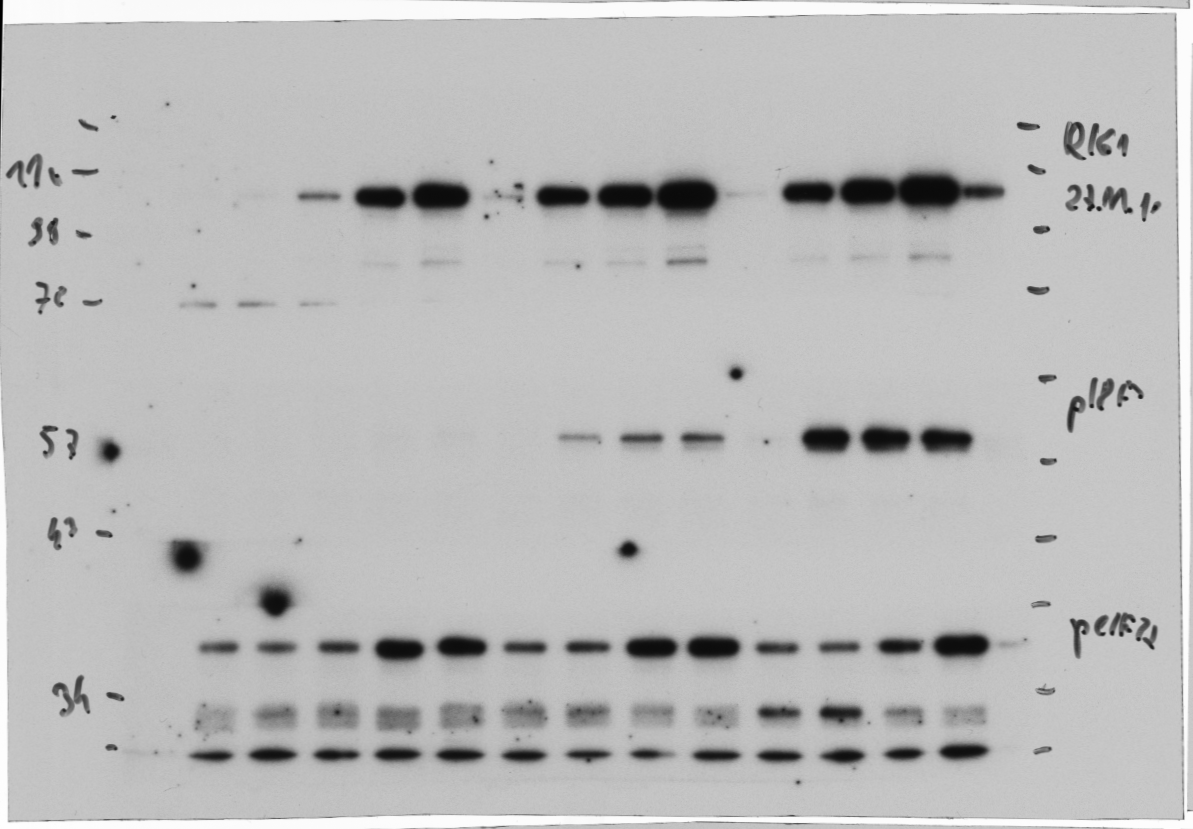

Supplement: S3 Dataset — (ZIP) [file ppat.1011597.s004.zip › S3_Dataset/Grabowski_et_al__Raw_Western_Blots/S1-Appendix--Figure-A--Panel-a--Western-blot/Raw files/p-IRF3.tif]

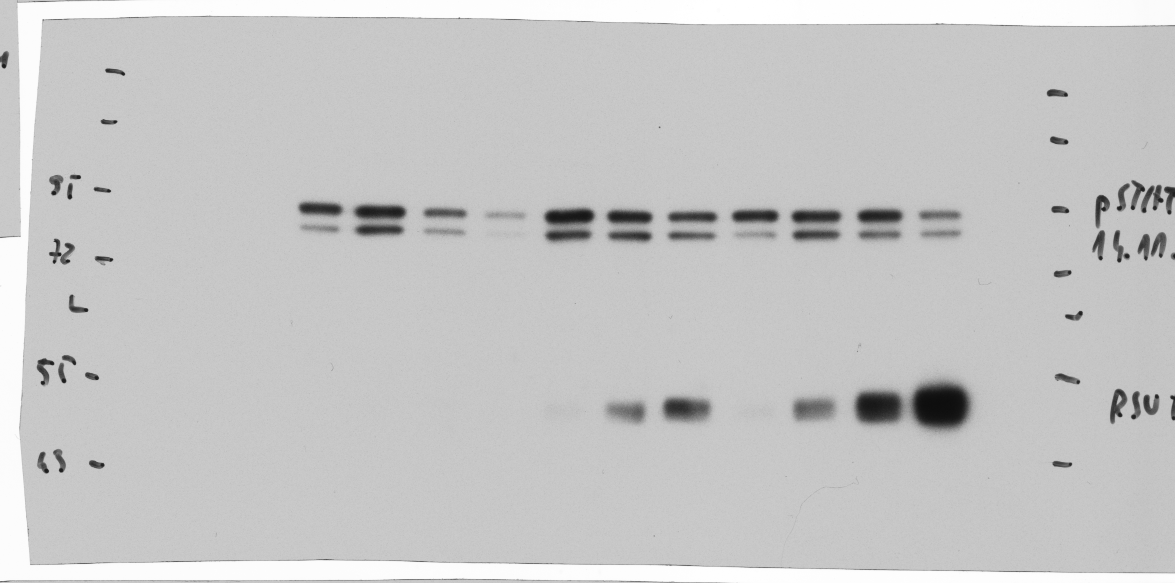

Supplement: S3 Dataset — (ZIP) [file ppat.1011597.s004.zip › S3_Dataset/Grabowski_et_al__Raw_Western_Blots/S1-Appendix--Figure-A--Panel-a--Western-blot/Raw files/p-STAT1.tif]

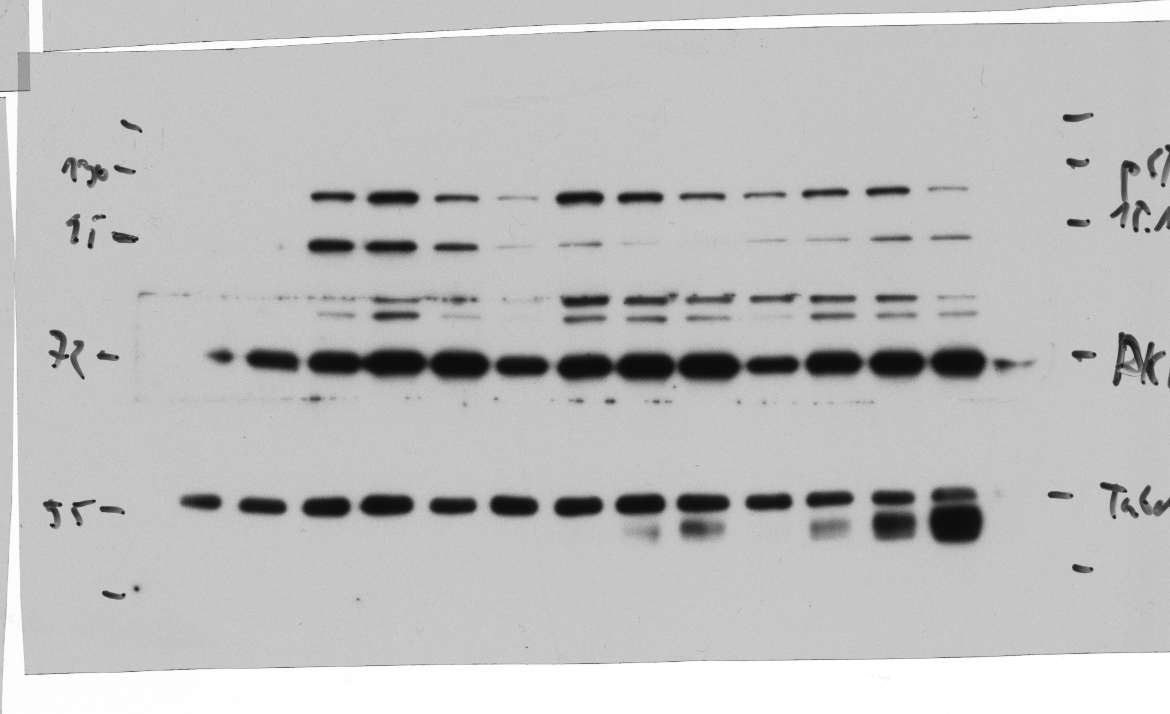

Supplement: S3 Dataset — (ZIP) [file ppat.1011597.s004.zip › S3_Dataset/Grabowski_et_al__Raw_Western_Blots/S1-Appendix--Figure-A--Panel-a--Western-blot/Raw files/p-STAT2.tif]

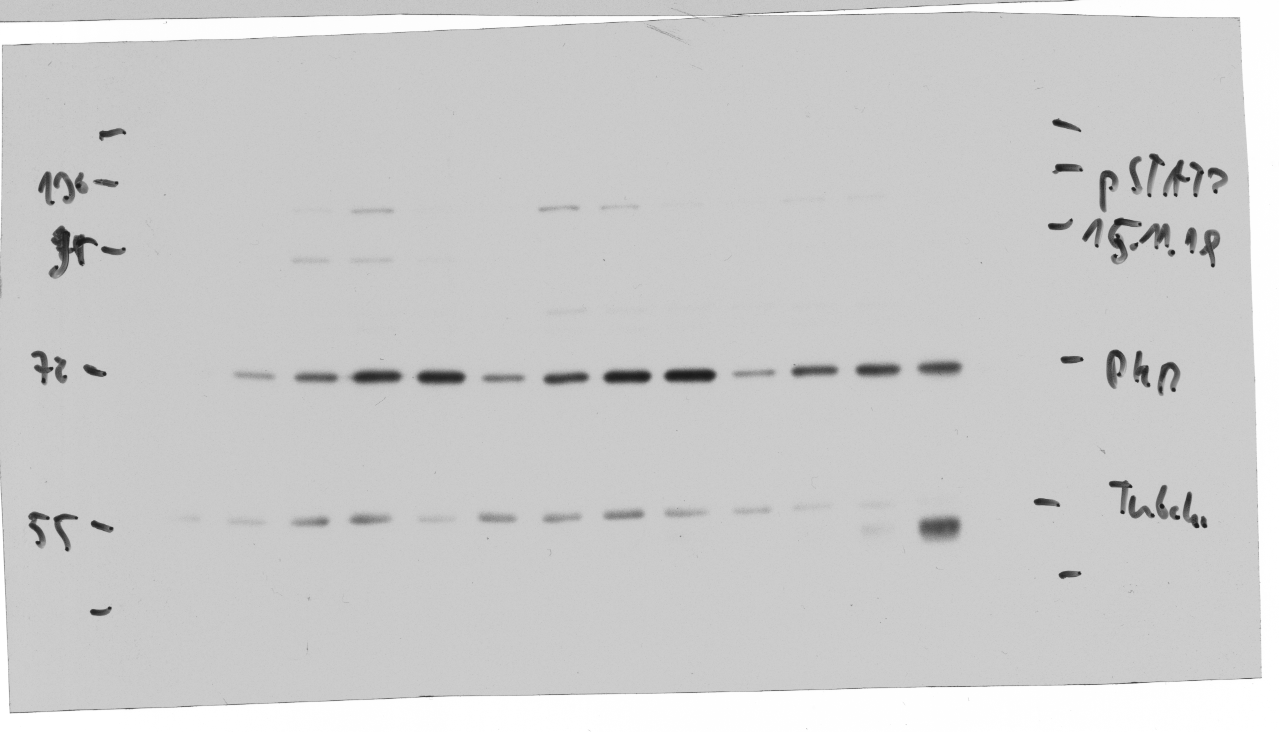

Supplement: S3 Dataset — (ZIP) [file ppat.1011597.s004.zip › S3_Dataset/Grabowski_et_al__Raw_Western_Blots/S1-Appendix--Figure-A--Panel-a--Western-blot/Raw files/PKR.tif]

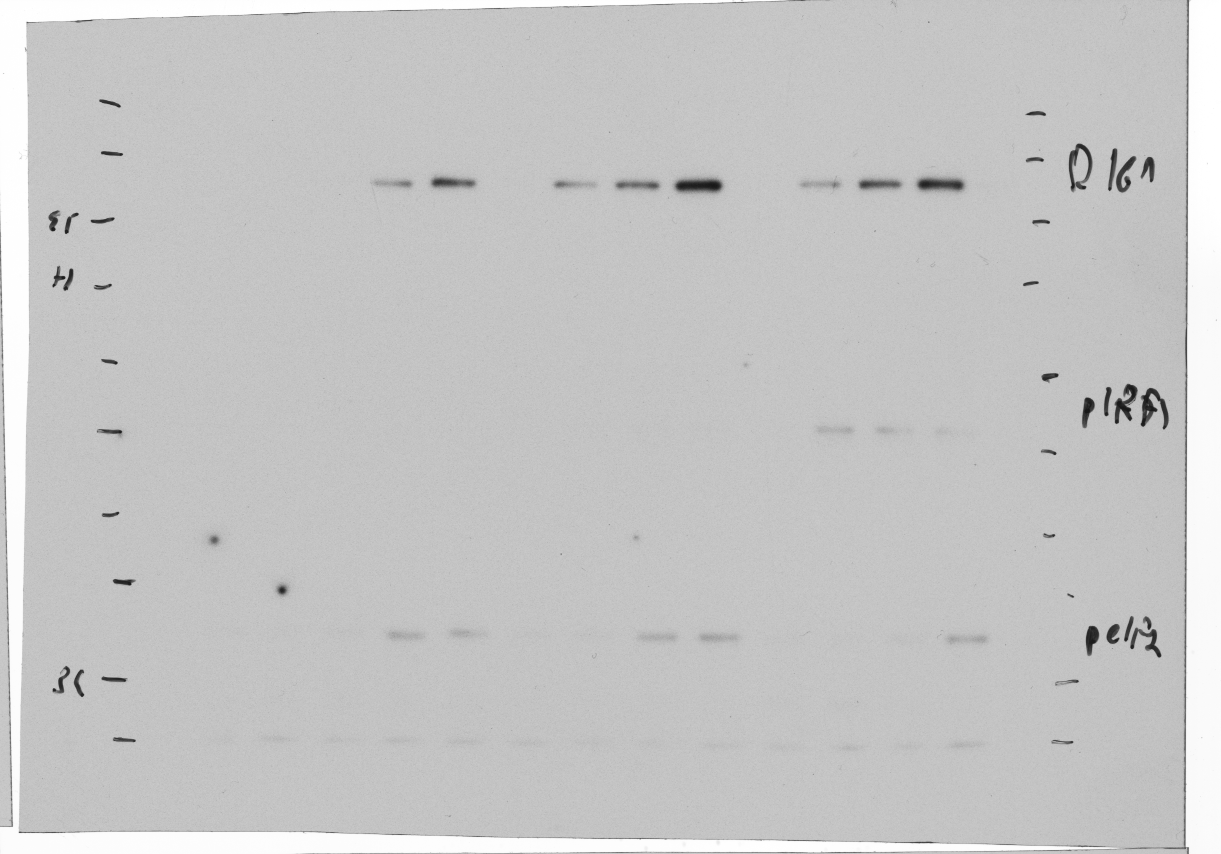

Supplement: S3 Dataset — (ZIP) [file ppat.1011597.s004.zip › S3_Dataset/Grabowski_et_al__Raw_Western_Blots/S1-Appendix--Figure-A--Panel-a--Western-blot/Raw files/RIG-I.tif]

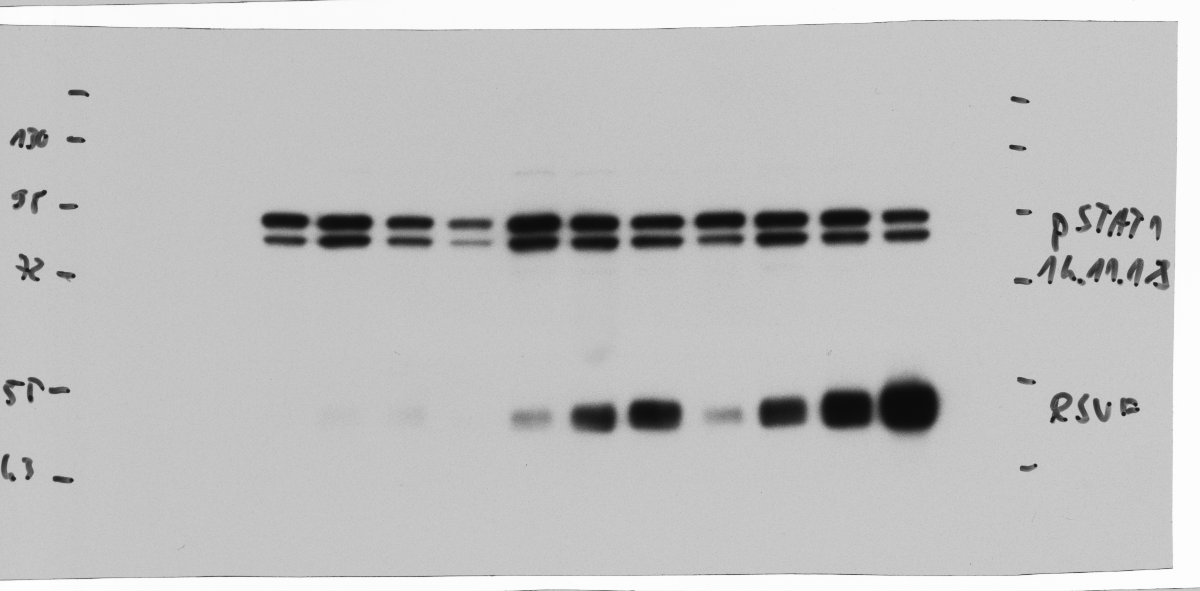

Supplement: S3 Dataset — (ZIP) [file ppat.1011597.s004.zip › S3_Dataset/Grabowski_et_al__Raw_Western_Blots/S1-Appendix--Figure-A--Panel-a--Western-blot/Raw files/RSV F.tif]

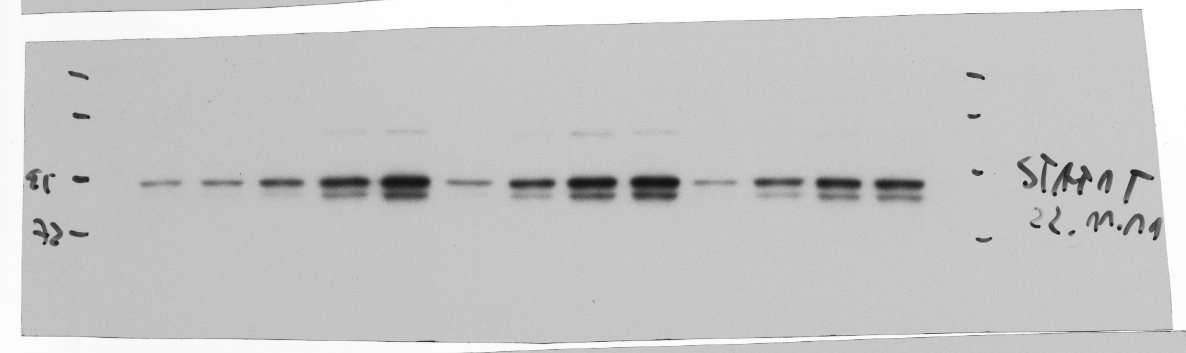

Supplement: S3 Dataset — (ZIP) [file ppat.1011597.s004.zip › S3_Dataset/Grabowski_et_al__Raw_Western_Blots/S1-Appendix--Figure-A--Panel-a--Western-blot/Raw files/STAT1.tif]

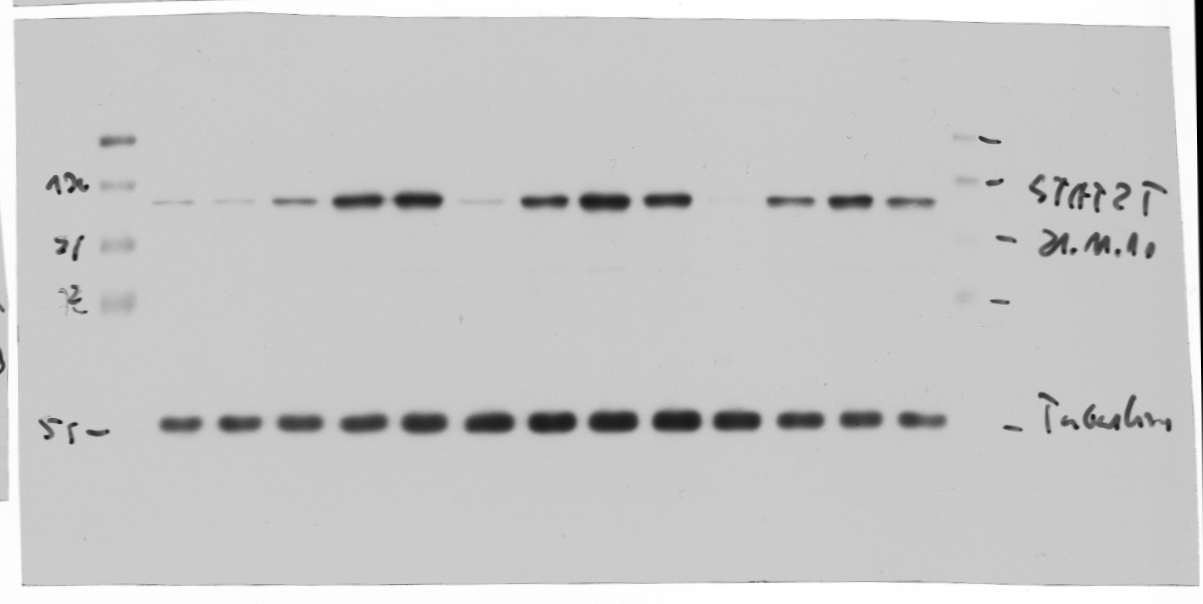

Supplement: S3 Dataset — (ZIP) [file ppat.1011597.s004.zip › S3_Dataset/Grabowski_et_al__Raw_Western_Blots/S1-Appendix--Figure-A--Panel-a--Western-blot/Raw files/STAT2.tif]

186 -

95 -

72 -

55 -

43 -

PSMT1  
14.11.10

RSVP

GAPDH

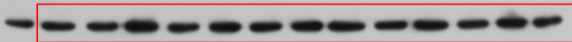

Supplement: S3 Dataset — (ZIP) [file ppat.1011597.s004.zip › S3_Dataset/Grabowski_et_al__Raw_Western_Blots/S1-Appendix--Figure-A--Panel-a--Western-blot/WB bands outlined/GAPDH.pdf]

48 -

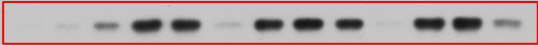

35 -

- OAS A

- 20.11.11

Supplement: S3 Dataset — (ZIP) [file ppat.1011597.s004.zip › S3_Dataset/Grabowski_et_al__Raw_Western_Blots/S1-Appendix--Figure-A--Panel-a--Western-blot/WB bands outlined/OAS1.pdf]

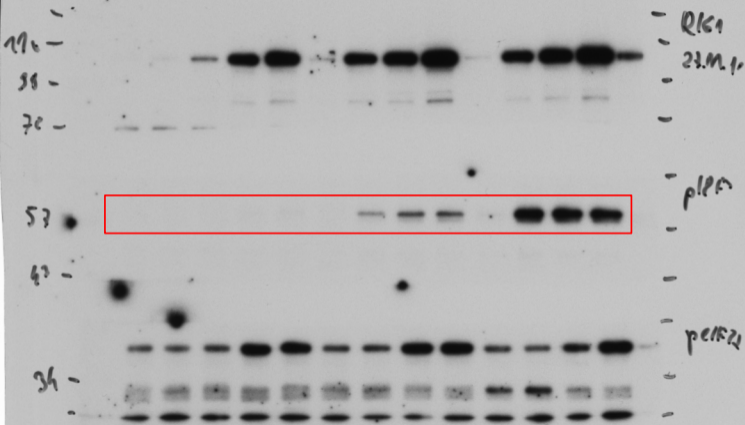

Supplement: S3 Dataset — (ZIP) [file ppat.1011597.s004.zip › S3_Dataset/Grabowski_et_al__Raw_Western_Blots/S1-Appendix--Figure-A--Panel-a--Western-blot/WB bands outlined/p-IRF3.pdf]

95 -

72 -

50 -

35 -

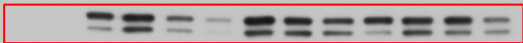

p57/47  
14.11.

RSU 7

Supplement: S3 Dataset — (ZIP) [file ppat.1011597.s004.zip › S3_Dataset/Grabowski_et_al__Raw_Western_Blots/S1-Appendix--Figure-A--Panel-a--Western-blot/WB bands outlined/p-STAT1.pdf]

130-  
115-

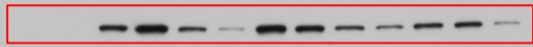

72-

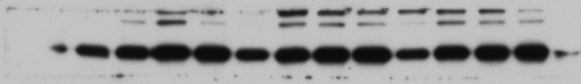

55-

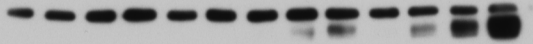

-  
-  
-

10.1

- AK

- Tub

Supplement: S3 Dataset — (ZIP) [file ppat.1011597.s004.zip › S3_Dataset/Grabowski_et_al__Raw_Western_Blots/S1-Appendix--Figure-A--Panel-a--Western-blot/WB bands outlined/p-STAT2.pdf]

136 -  
95 -

72 -

55 -

- pSTAT7?  
- 15-M. 18

- Phn

- Tubulin

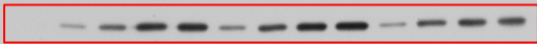

Supplement: S3 Dataset — (ZIP) [file ppat.1011597.s004.zip › S3_Dataset/Grabowski_et_al__Raw_Western_Blots/S1-Appendix--Figure-A--Panel-a--Western-blot/WB bands outlined/PKR.pdf]

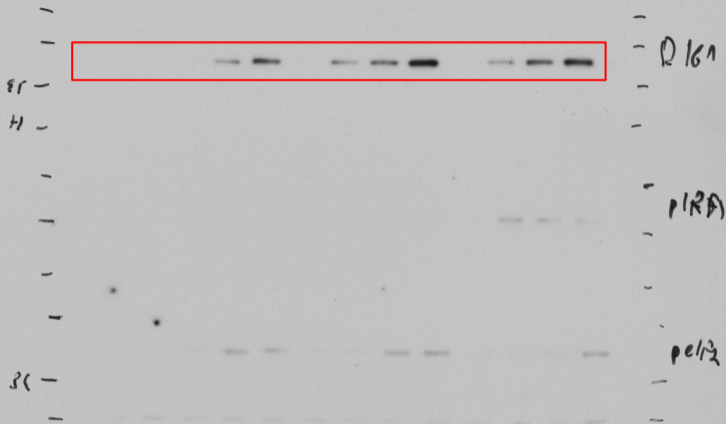

Supplement: S3 Dataset — (ZIP) [file ppat.1011597.s004.zip › S3_Dataset/Grabowski_et_al__Raw_Western_Blots/S1-Appendix--Figure-A--Panel-a--Western-blot/WB bands outlined/RIG-I.pdf]

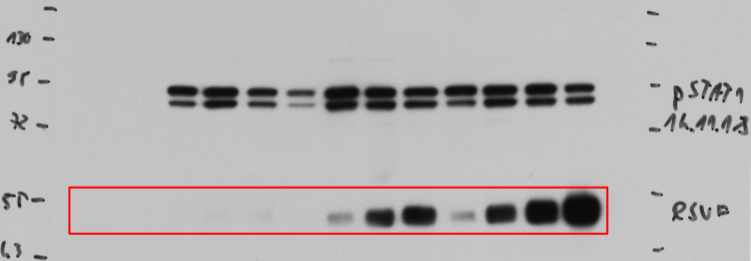

Supplement: S3 Dataset — (ZIP) [file ppat.1011597.s004.zip › S3_Dataset/Grabowski_et_al__Raw_Western_Blots/S1-Appendix--Figure-A--Panel-a--Western-blot/WB bands outlined/RSV F.pdf]

85 -

72 -

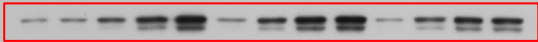

START

22.11.19

Supplement: S3 Dataset — (ZIP) [file ppat.1011597.s004.zip › S3_Dataset/Grabowski_et_al__Raw_Western_Blots/S1-Appendix--Figure-A--Panel-a--Western-blot/WB bands outlined/STAT1.pdf]

126

21

22

55-

STATT2T

M.M.10

Tubulin

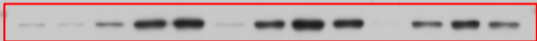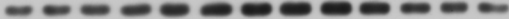

Supplement: S3 Dataset — (ZIP) [file ppat.1011597.s004.zip › S3_Dataset/Grabowski_et_al__Raw_Western_Blots/S1-Appendix--Figure-A--Panel-a--Western-blot/WB bands outlined/STAT2.pdf]

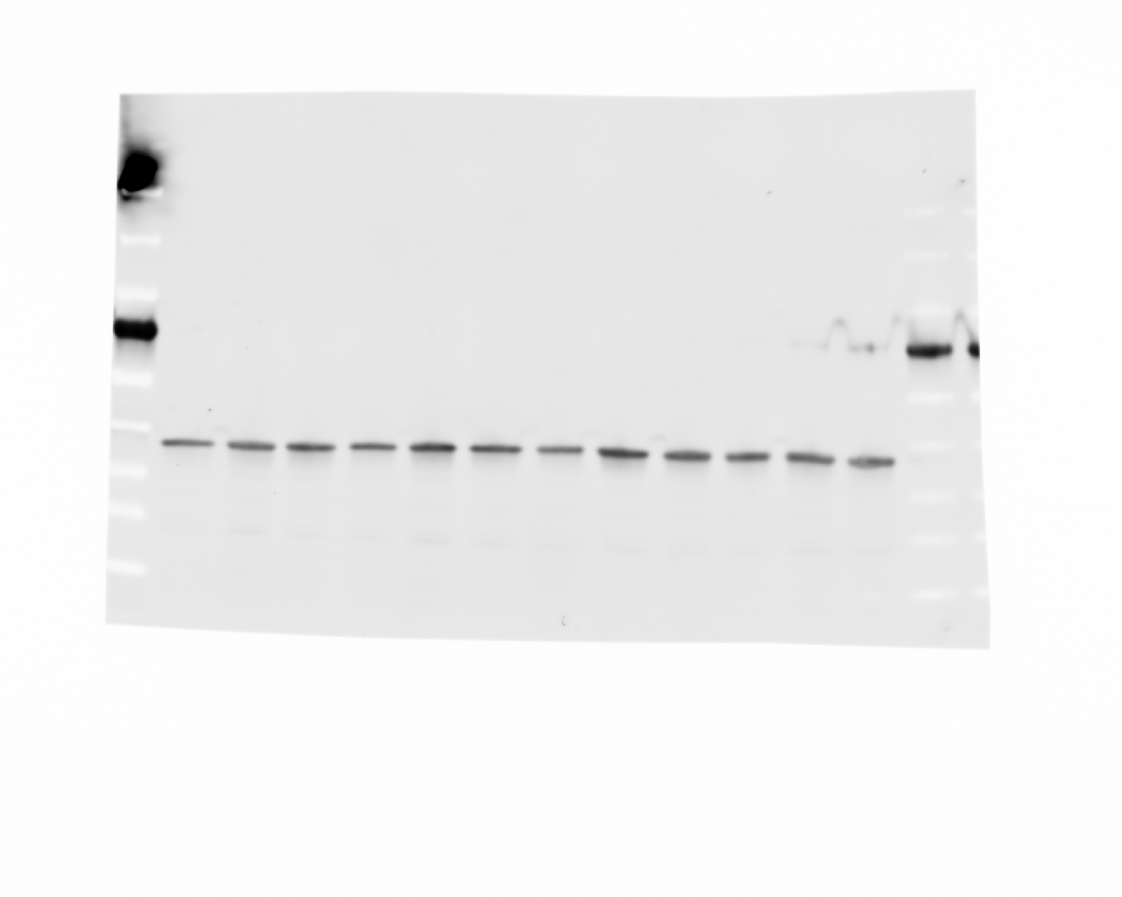

Supplement: S3 Dataset — (ZIP) [file ppat.1011597.s004.zip › S3_Dataset/Grabowski_et_al__Raw_Western_Blots/S1-Appendix--Figure-B--Panel-a--Western-blot/Raw files/GAPDH.tif]

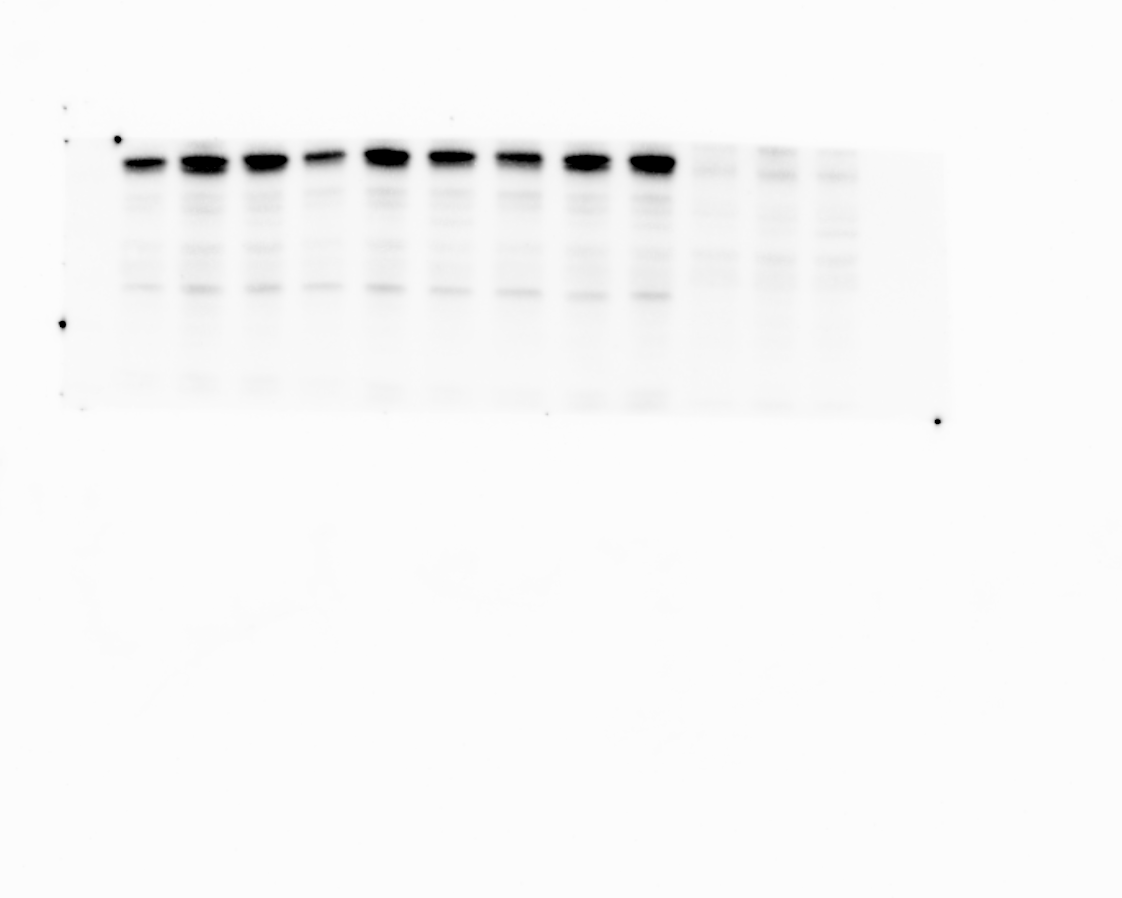

Supplement: S3 Dataset — (ZIP) [file ppat.1011597.s004.zip › S3_Dataset/Grabowski_et_al__Raw_Western_Blots/S1-Appendix--Figure-B--Panel-a--Western-blot/Raw files/IRF3.tif]

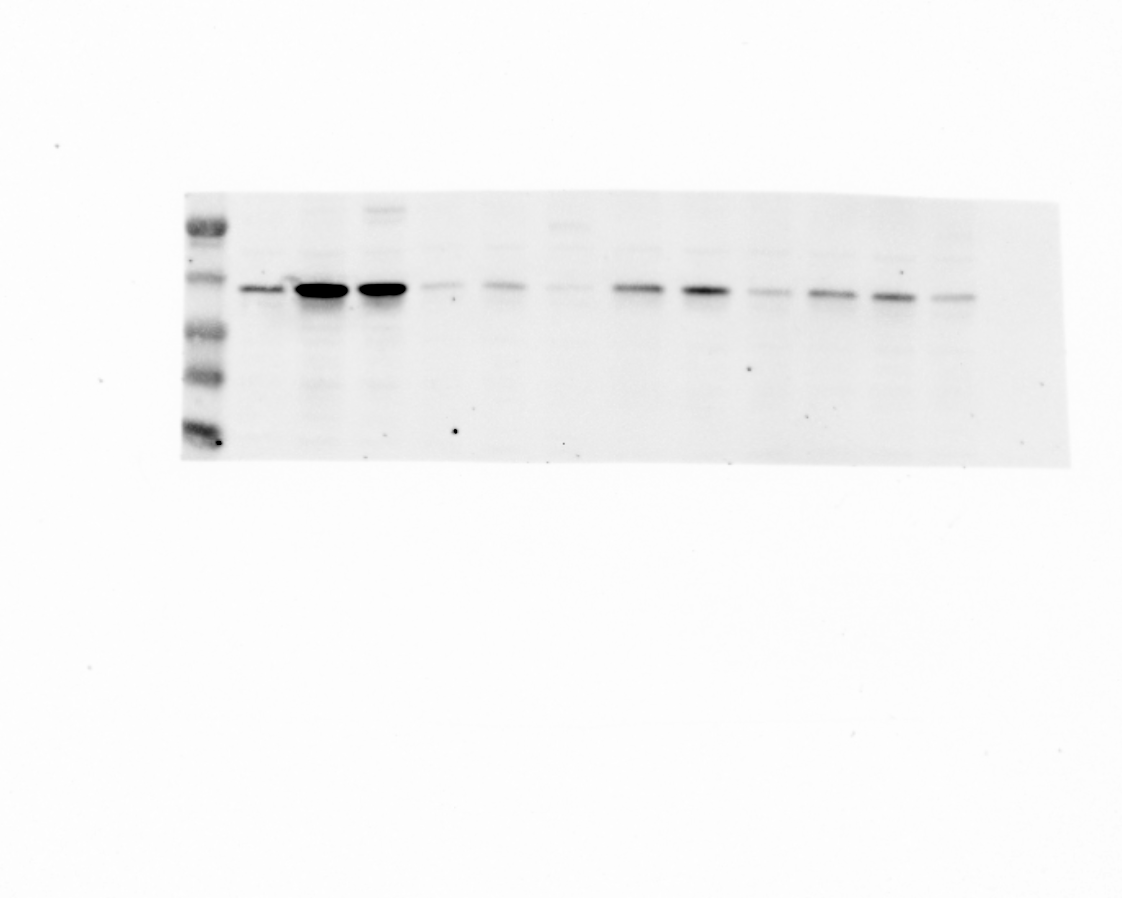

Supplement: S3 Dataset — (ZIP) [file ppat.1011597.s004.zip › S3_Dataset/Grabowski_et_al__Raw_Western_Blots/S1-Appendix--Figure-B--Panel-a--Western-blot/Raw files/OAS1.tif]

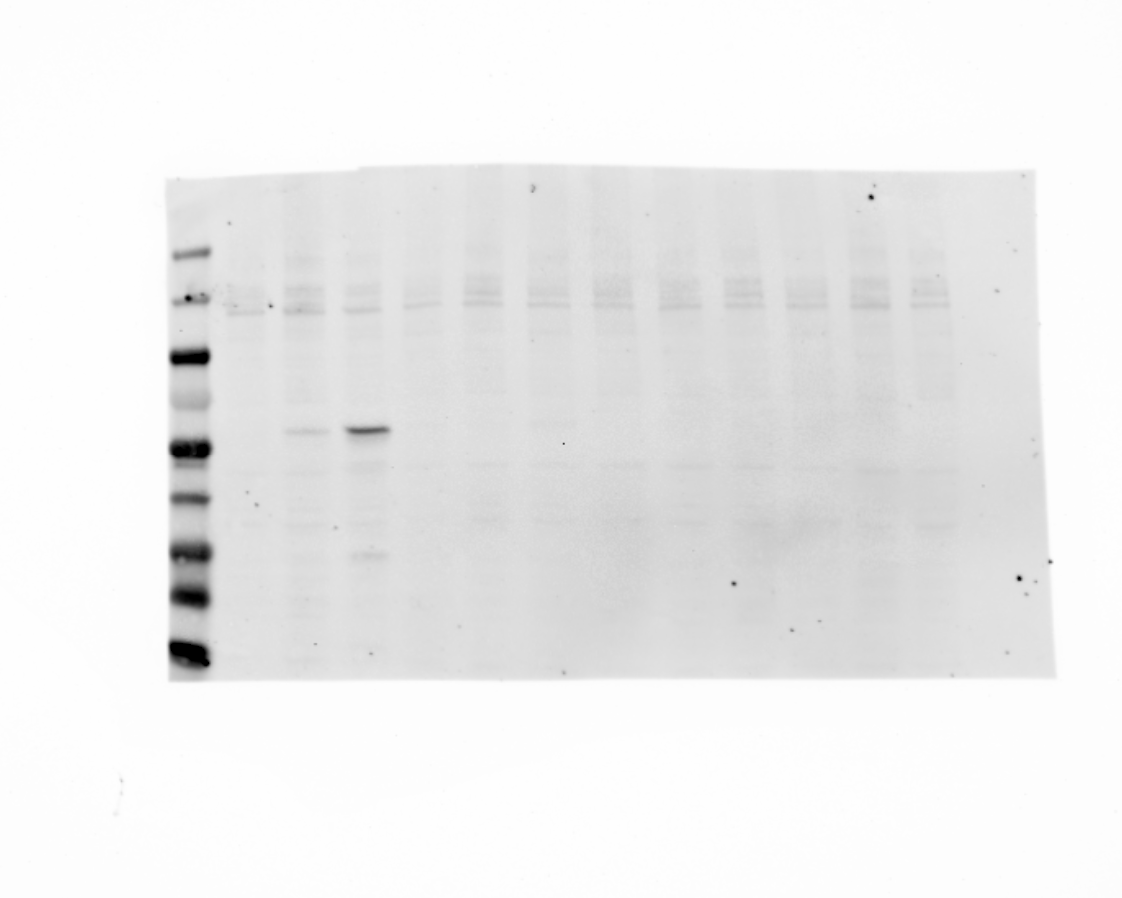

Supplement: S3 Dataset — (ZIP) [file ppat.1011597.s004.zip › S3_Dataset/Grabowski_et_al__Raw_Western_Blots/S1-Appendix--Figure-B--Panel-a--Western-blot/Raw files/p-IRF3.tif]

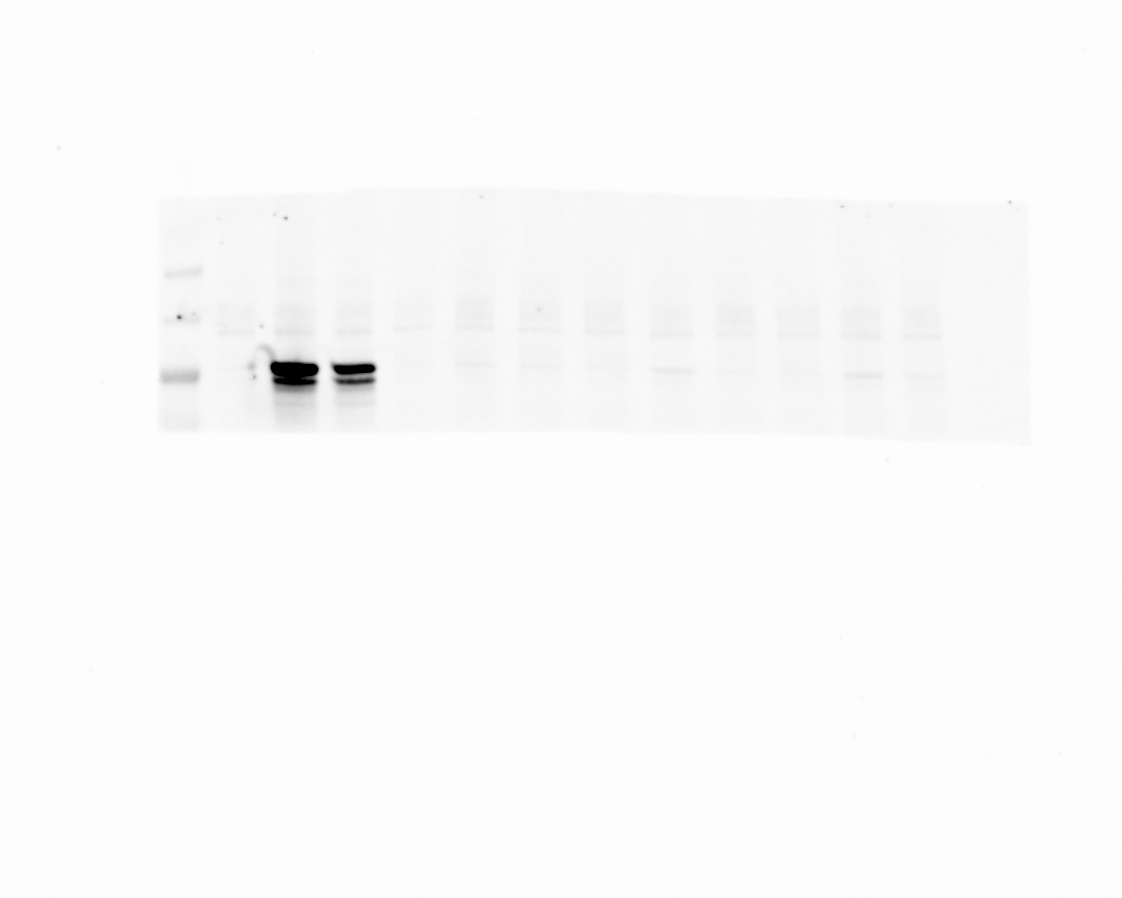

Supplement: S3 Dataset — (ZIP) [file ppat.1011597.s004.zip › S3_Dataset/Grabowski_et_al__Raw_Western_Blots/S1-Appendix--Figure-B--Panel-a--Western-blot/Raw files/p-STAT1.tif]

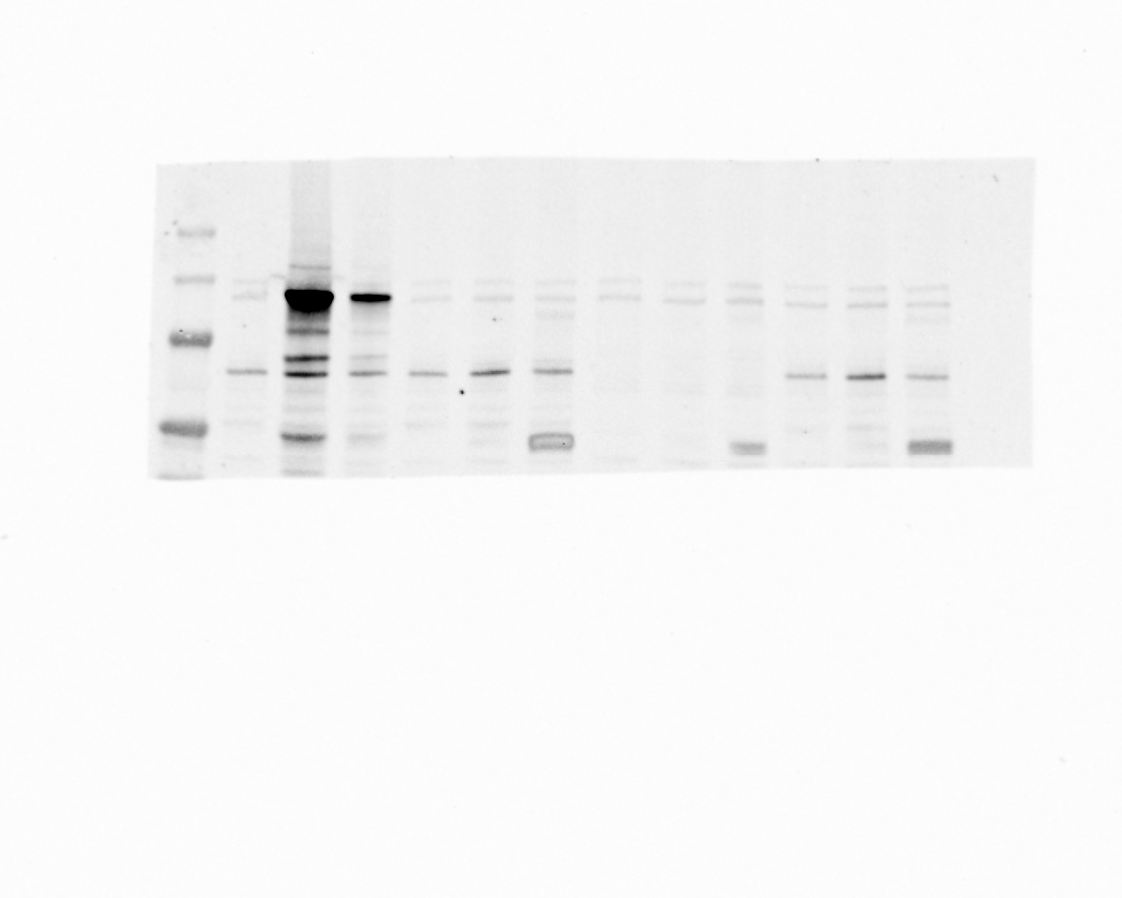

Supplement: S3 Dataset — (ZIP) [file ppat.1011597.s004.zip › S3_Dataset/Grabowski_et_al__Raw_Western_Blots/S1-Appendix--Figure-B--Panel-a--Western-blot/Raw files/p-STAT2.tif]

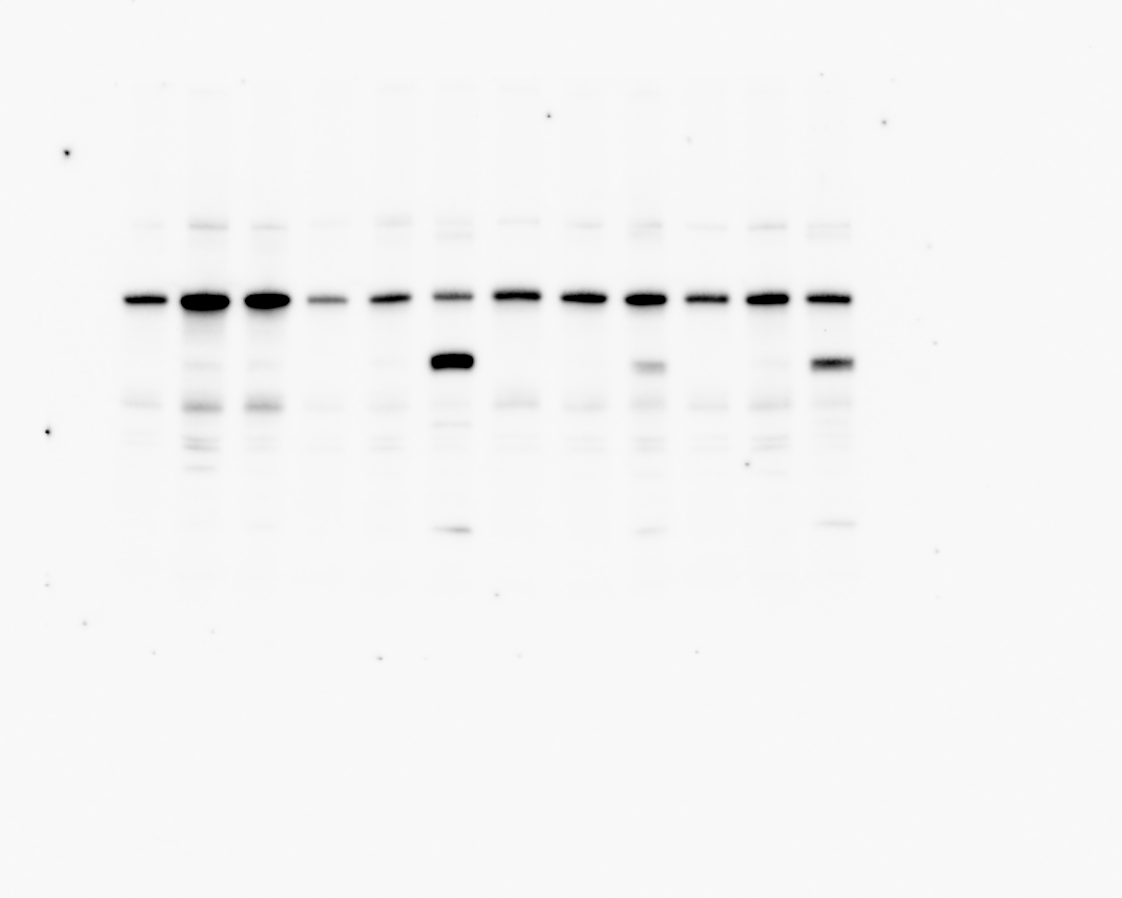

Supplement: S3 Dataset — (ZIP) [file ppat.1011597.s004.zip › S3_Dataset/Grabowski_et_al__Raw_Western_Blots/S1-Appendix--Figure-B--Panel-a--Western-blot/Raw files/PKR.tif]

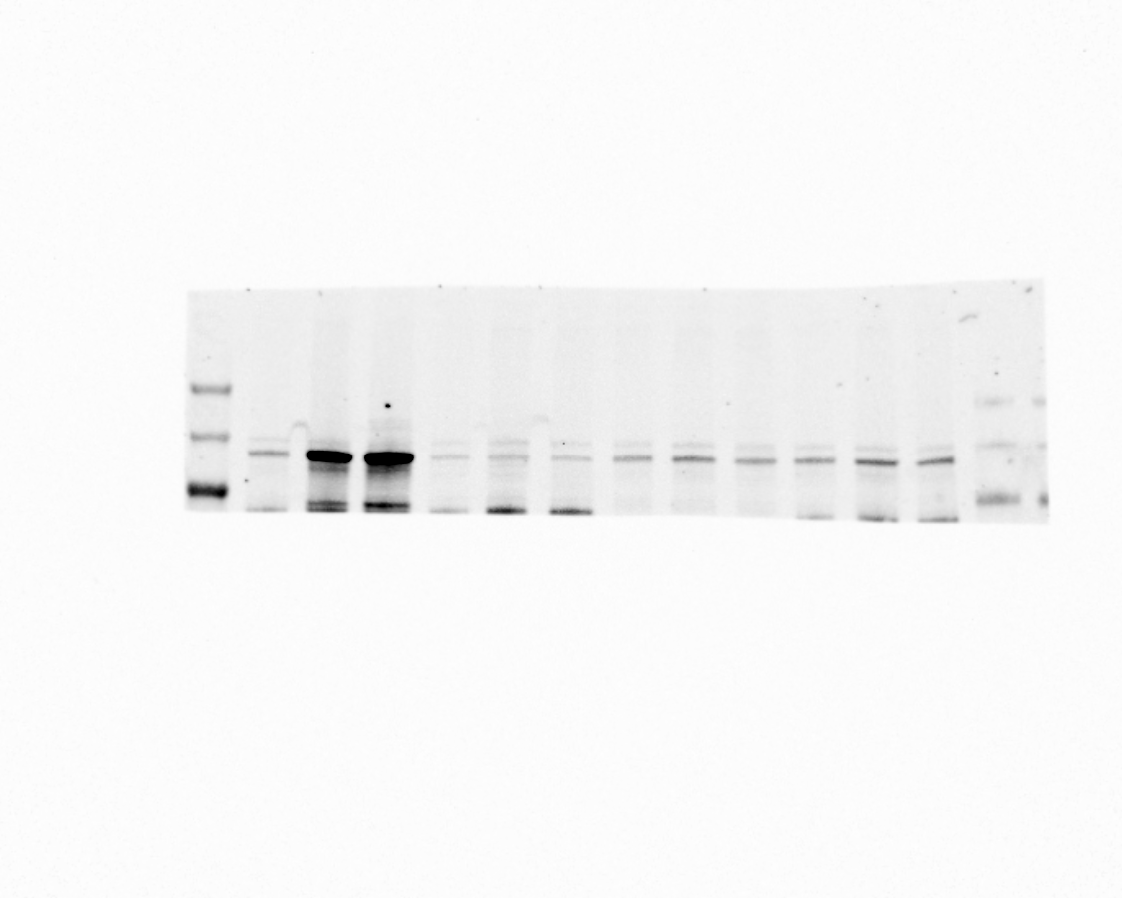

Supplement: S3 Dataset — (ZIP) [file ppat.1011597.s004.zip › S3_Dataset/Grabowski_et_al__Raw_Western_Blots/S1-Appendix--Figure-B--Panel-a--Western-blot/Raw files/RIG-I.tif]

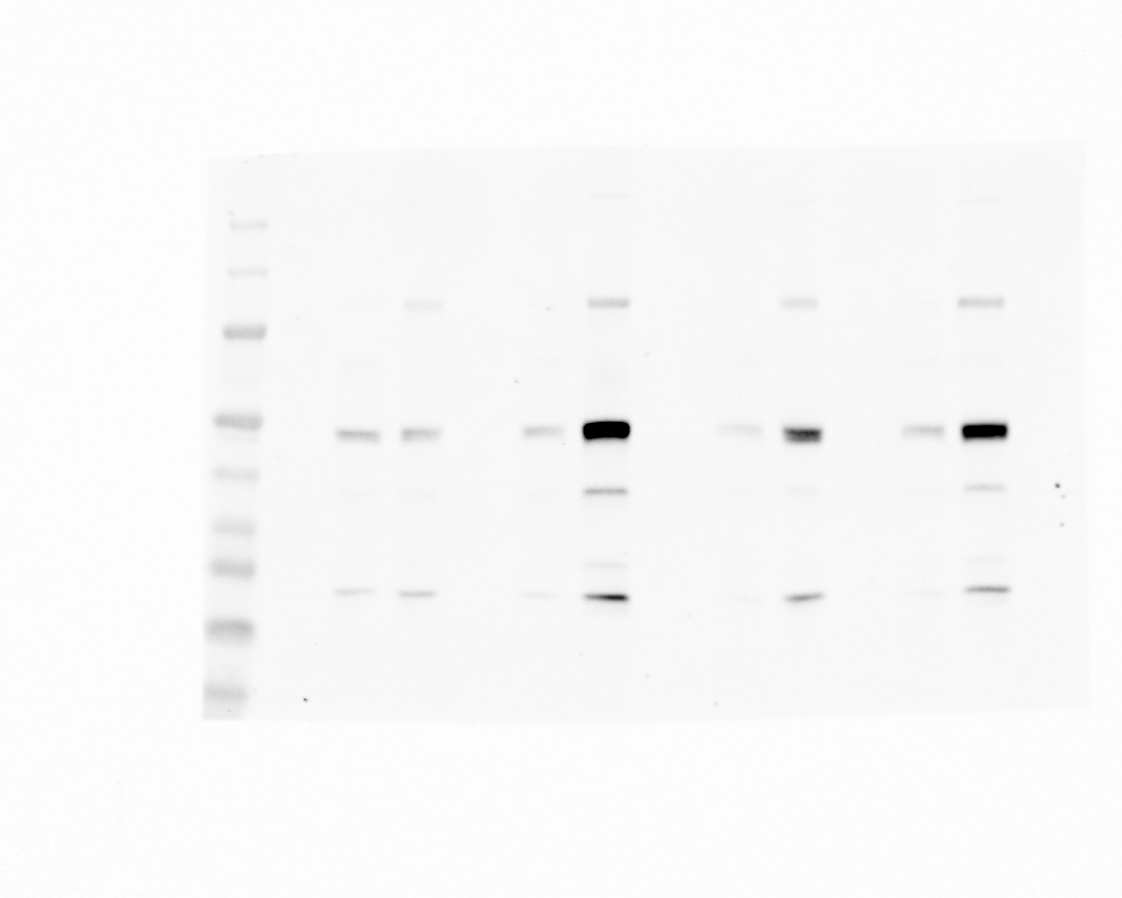

Supplement: S3 Dataset — (ZIP) [file ppat.1011597.s004.zip › S3_Dataset/Grabowski_et_al__Raw_Western_Blots/S1-Appendix--Figure-B--Panel-a--Western-blot/Raw files/RSV F.tif]

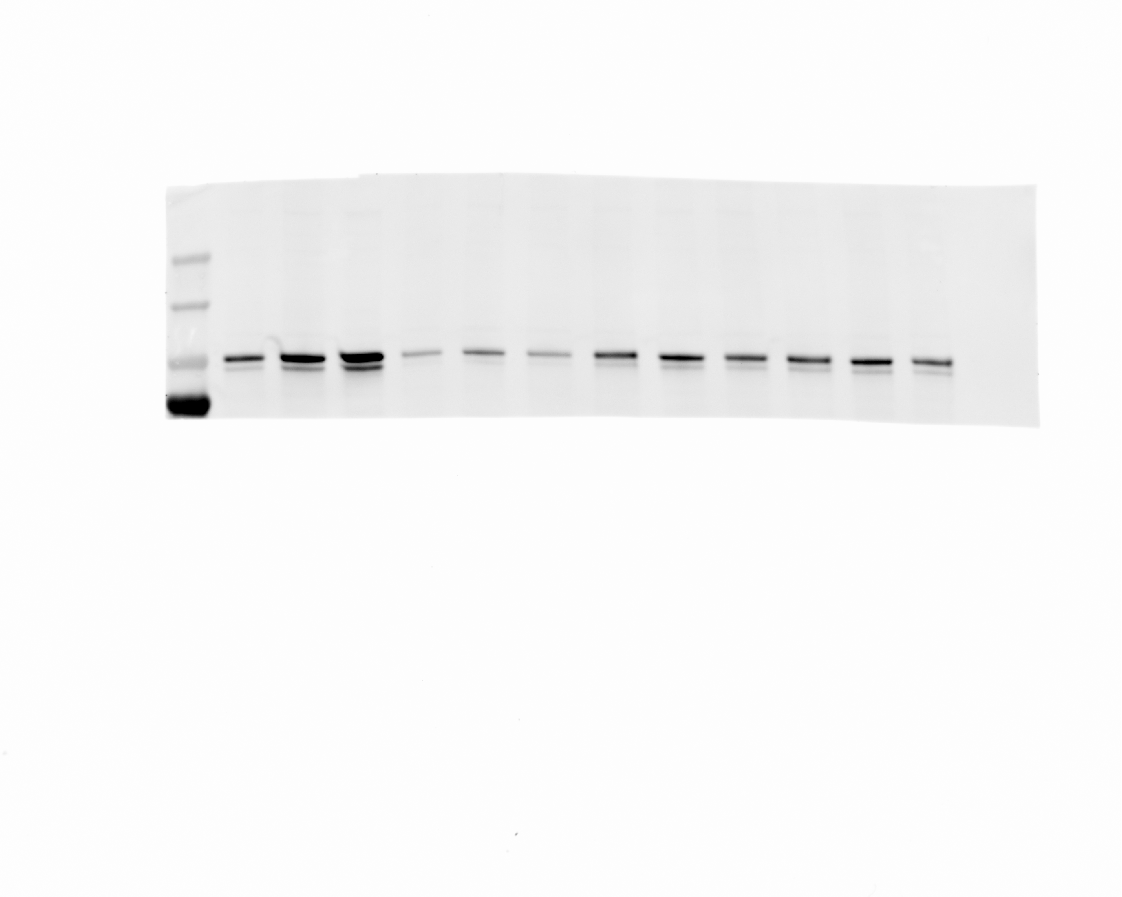

Supplement: S3 Dataset — (ZIP) [file ppat.1011597.s004.zip › S3_Dataset/Grabowski_et_al__Raw_Western_Blots/S1-Appendix--Figure-B--Panel-a--Western-blot/Raw files/STAT1.tif]

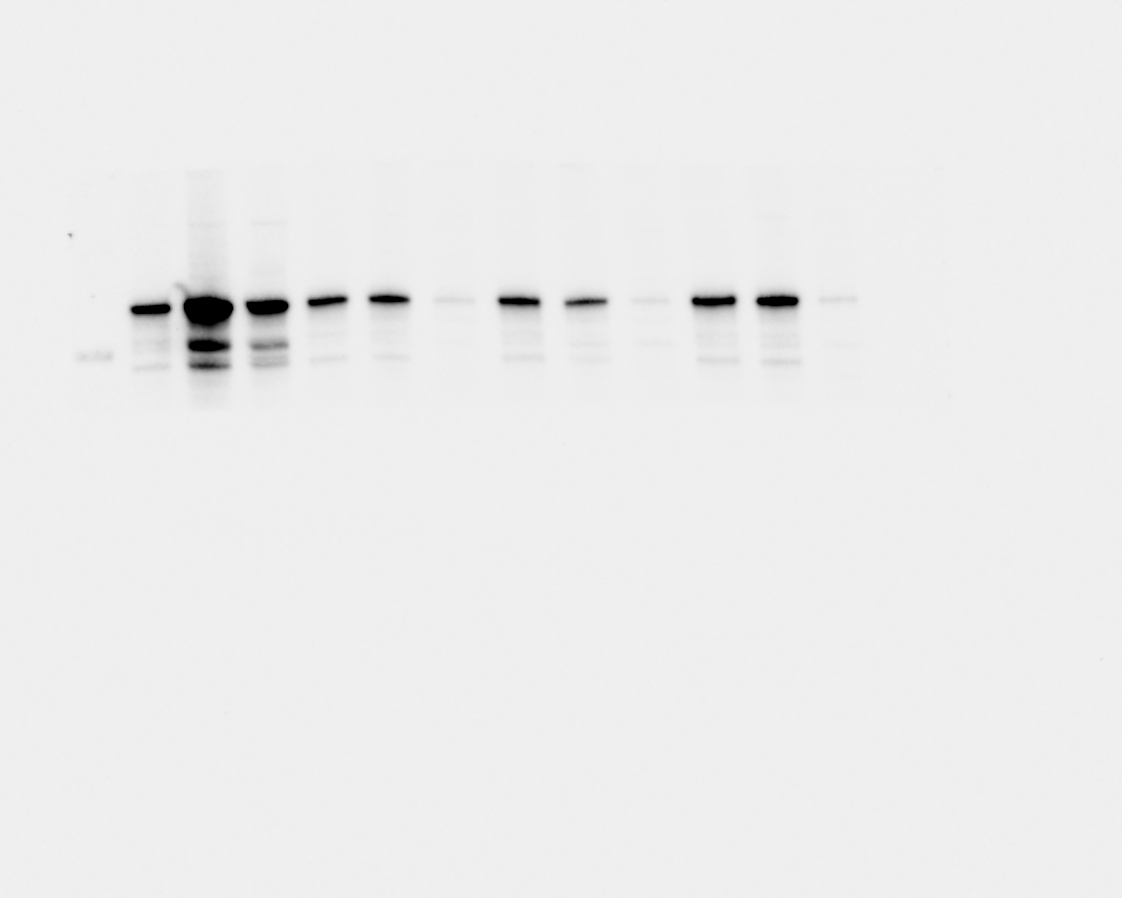

Supplement: S3 Dataset — (ZIP) [file ppat.1011597.s004.zip › S3_Dataset/Grabowski_et_al__Raw_Western_Blots/S1-Appendix--Figure-B--Panel-a--Western-blot/Raw files/STAT2.tif]

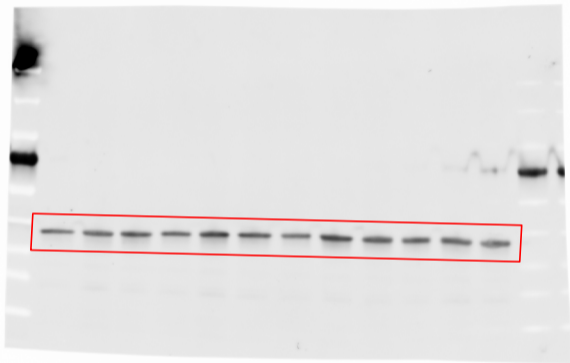

Supplement: S3 Dataset — (ZIP) [file ppat.1011597.s004.zip › S3_Dataset/Grabowski_et_al__Raw_Western_Blots/S1-Appendix--Figure-B--Panel-a--Western-blot/WB bands outlined/GAPDH.pdf]

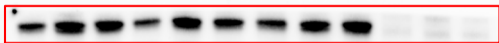

Supplement: S3 Dataset — (ZIP) [file ppat.1011597.s004.zip › S3_Dataset/Grabowski_et_al__Raw_Western_Blots/S1-Appendix--Figure-B--Panel-a--Western-blot/WB bands outlined/IRF3.pdf]

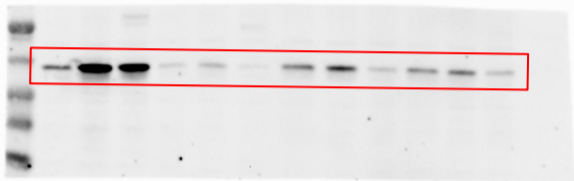

Supplement: S3 Dataset — (ZIP) [file ppat.1011597.s004.zip › S3_Dataset/Grabowski_et_al__Raw_Western_Blots/S1-Appendix--Figure-B--Panel-a--Western-blot/WB bands outlined/OAS1.pdf]

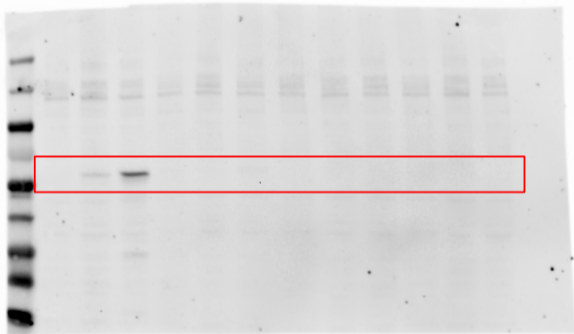

Supplement: S3 Dataset — (ZIP) [file ppat.1011597.s004.zip › S3_Dataset/Grabowski_et_al__Raw_Western_Blots/S1-Appendix--Figure-B--Panel-a--Western-blot/WB bands outlined/p-IRF3.pdf]

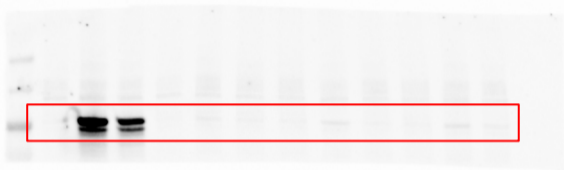

Supplement: S3 Dataset — (ZIP) [file ppat.1011597.s004.zip › S3_Dataset/Grabowski_et_al__Raw_Western_Blots/S1-Appendix--Figure-B--Panel-a--Western-blot/WB bands outlined/p-STAT1.pdf]

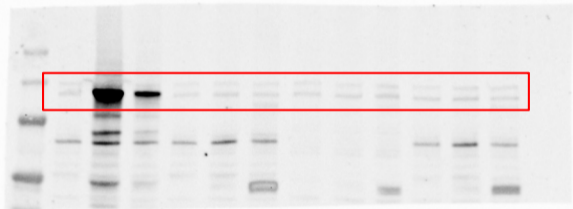

Supplement: S3 Dataset — (ZIP) [file ppat.1011597.s004.zip › S3_Dataset/Grabowski_et_al__Raw_Western_Blots/S1-Appendix--Figure-B--Panel-a--Western-blot/WB bands outlined/p-STAT2.pdf]

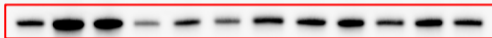

Supplement: S3 Dataset — (ZIP) [file ppat.1011597.s004.zip › S3_Dataset/Grabowski_et_al__Raw_Western_Blots/S1-Appendix--Figure-B--Panel-a--Western-blot/WB bands outlined/PKR.pdf]

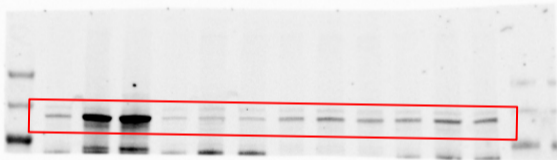

Supplement: S3 Dataset — (ZIP) [file ppat.1011597.s004.zip › S3_Dataset/Grabowski_et_al__Raw_Western_Blots/S1-Appendix--Figure-B--Panel-a--Western-blot/WB bands outlined/RIG-I.pdf]

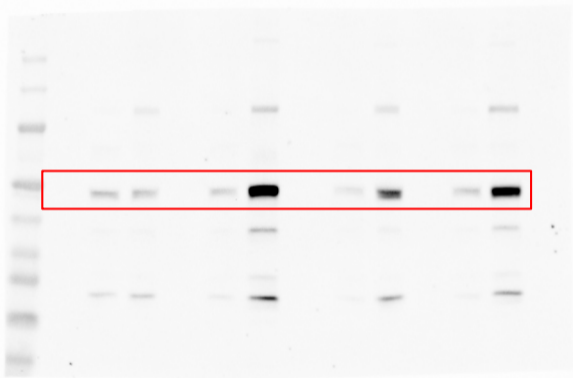

Supplement: S3 Dataset — (ZIP) [file ppat.1011597.s004.zip › S3_Dataset/Grabowski_et_al__Raw_Western_Blots/S1-Appendix--Figure-B--Panel-a--Western-blot/WB bands outlined/RSV F.pdf]

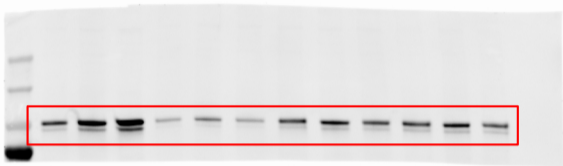

Supplement: S3 Dataset — (ZIP) [file ppat.1011597.s004.zip › S3_Dataset/Grabowski_et_al__Raw_Western_Blots/S1-Appendix--Figure-B--Panel-a--Western-blot/WB bands outlined/STAT1.pdf]

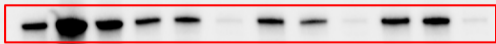

Supplement: S3 Dataset — (ZIP) [file ppat.1011597.s004.zip › S3_Dataset/Grabowski_et_al__Raw_Western_Blots/S1-Appendix--Figure-B--Panel-a--Western-blot/WB bands outlined/STAT2.pdf]
