# Supplementary material for: Antagonism between viral infection and innate immunity at the single-cell level
Source: PLoS Pathog. 2023 Sep 5;19(9):e1011597. doi: 10.1371/journal.ppat.1011597 (PMC10503725; doi:10.1371/journal.ppat.1011597)
Supplement: Attachment — Submitted filename: Grabowski_et_al--Response_to_reviewers.pdf [file ppat.1011597.s005.pdf]

IPPT PAN
Institute of Fundamental Technological Research
of the Polish Academy of Sciences
Pawińskiego 5B, 02-106 Warsaw, Poland
Department of Biosystems and Soft Matter
Laboratory of Modeling in Biology and Medicine
https://pmbm.ippt.pan.pl

June 23, 2023

Dear Editor,  Dear Reviewers,

Thank you for your careful reading and thorough review of our manuscript "Antagonism between viral infection and innate immunity at the single-cell level".  To address your concerns, we have revised our manuscript.  The main changes introduced in the revision are as follows:

- We expanded *Discussion* to place our results in a broader context of IFN signaling and included additional references in Introduction.
- We included two new supplementary figures:
  - new Appendix Fig S7 shows correlation between nuclear translocation (activation) of RelA (a subunit of NF-κB) and IRF3;
  - new Appendix Fig S8 demonstrates that brefeldin A (BFA) treatment facilitates visualization of IFNβ-producing cells.
- We modified Fig 2C, which now shows snapshots from the same model simulation.

Detailed point-by-point responses to your concerns are provided on the next pages of this document. A separate PDF document shows color-marked changes introduced in the text during the revision. (For clarity, the tracking changes file shows references to bibliography in the Author-Year format, because this format was used in the initial submission.  Per journal guidelines, the final manuscript file has numeric references.)

We hope that you will find the revised manuscript suitable for publication in *PLOS Pathogens*.

Sincerely,
Tomasz Lipniacki, Ph.D.

# Response to the Report from Reviewer #1

## **Part I** – Summary

*Please use this section to discuss strengths/weaknesses of study, novelty/significance, general execution and scholarship.*

> **1.** This is a very interesting study, collecting single cell observations and using them to model RSV-induced interferon activity. The model is very interesting and distinct from models in this space. And the work suggests a new possible mechanisms of RSV inhibition of host signaling. The biggest weakness, repeated below, is that the findings are not integrated into what is known about IFN signaling. Better integration of these findings with the current literature is key.

**Response:** Thank you for the overall favorable assessment. In the revised manuscript we expanded Discussion towards IFN signaling to highlight the accordance of the model and existing experimental results with respect to: (i) the timing of the onset of IFN production, (ii) the fraction of IFN-producing cells, (iii) spatial heterogeneity of IFN-activated cells. Also, in Introduction we provided references to the description of multi-layered negative regulation of the immune response conferred by RSV proteins.

## **Part II** – Major Issues: Key Experiments Required for Acceptance

*Please use this section to detail the key new experiments or modifications of existing experiments that should be absolutely required to validate study conclusions.*

> **2.** (**major**) please clarify for the reader how much of the model was tuned or trained using the data discussed later in the work.

**Response:** Indeed, it was not clearly stated in the manuscript. Values of all model parameters were assigned based on experimental data. Overall, parameter values were initially constrained based on cell-population experiments, and then further manually adjusted based on single-cell data. Most increase/decrease rates were directly estimated from Western blots (e.g. the rates for `STATs` and `ISGs` – from Appendix Fig S3). Feedback strengths required more careful, global tuning based on IFNβ pre-stimulation experiments (Appendix Fig S5) and single-cell statistics (Fig 3C, Fig 4BC, and Fig 5BC). Finally, rates for components `v` and `vRNA`, which are not observed directly, have been selected based on the timing of viral protein appearance in immunostaining images, including the timing and number of secondary infections. Still, we concede that some parameters are more tentative than others, especially the feedback parameters. Because of the greater uncertainty in the precise values of these parameters, we investigate how model predictions are influenced by changes

of the relevant feedback parameters (in Figs 3–6). We amended the manuscript providing information which datasets were used to constrain parameters.

> **3.** (**major**) what was the point of the BFA treatment? As this paper will be read by modelers, some detail on what the BFA experiment reveals is important.

**Response:** Brefeldin A blocks secretion of IFNβ, which accumulates inside IFNβ-producing cells and can be then readily visualized by immunofluorescence staining. Without the use of BFA, production of IFNβ is sometimes still noticeable (as shown in the figure below, provided in the revised manuscript as Fig S8A), but quantification is much less reliable.

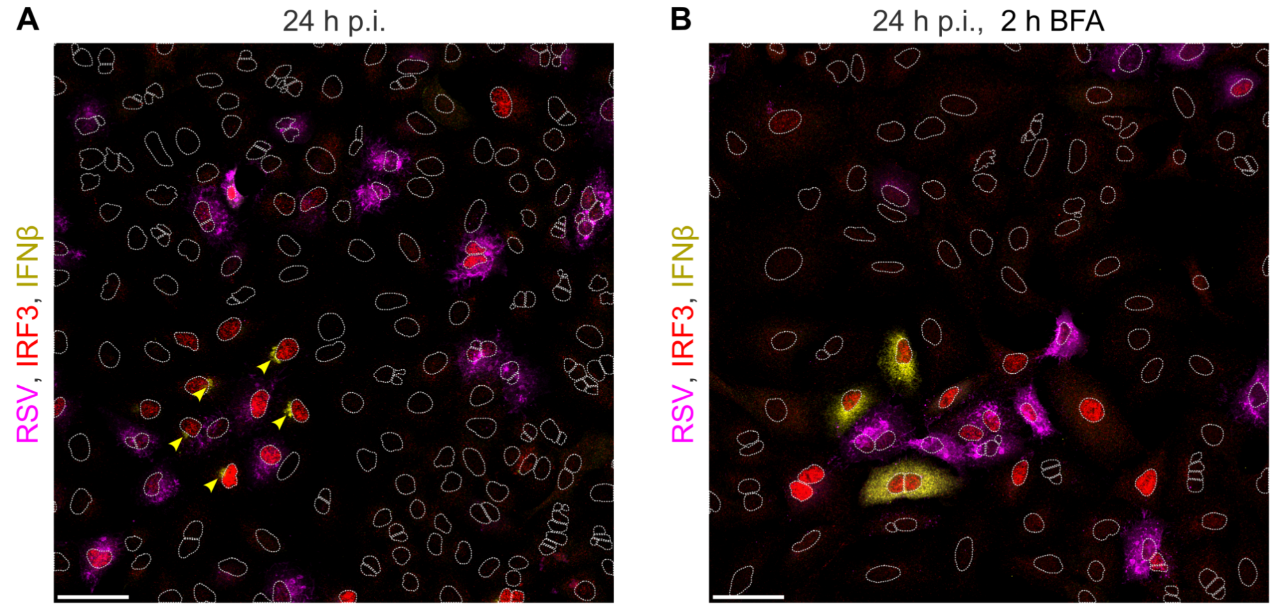


Importantly, irrespective of using BFA or not, IFNβ appears preferentially in cells devoid of RSV proteins, which is one of our key observations. We updated captions of Fig 2 and Fig 5 as well as the discussion of Fig 2 in the main text to clarify this point.

> **4.** (**major**) fig 2c is not discussed in the relevant section.

**Response:** We added a brief discussion of (updated) Fig 2C, see Results subsection *Model simulations vs. immunostaining images* in the revised Manuscript. The key advantage of computer simulations is that they allow us to study the same cell population at different time points (whereas after immunostaining we observe snapshots from different wells), and we can simultaneously record all components of the model. Analogous coherence and breadth are beyond the reach of experimental techniques.

> **5.** (minor) the sKs metric is odd and not well know. Should provide some explanation or just use a median or mean value and report the KS p value.

**Response:** We prefer to use sKS (signed Kolmogorov–Smirnov) statistic rather than a pure KS statistic, because in certain cases (for example in Fig 3E) the compared distributions exchange their places. It also allows us to differentiate between an sKS value which is consistently low and positive or low and negative for all experimental replicates, and noisy sKS value changing sign between replicates. However, we acknowledge that the sKS statistic is not very well known, and can be problematic when a narrower distribution is nested within a broader one (in fact, we use the sKS statistic only Figs 3B–E). We included an explanation in the revised manuscript.

> **6.** (**major**) fig 3c, the fit to the data is pretty poor. Given the complexity of the system, this may be reasonable but worth discussing briefly in the results section.

**Response:** Yes, we agree that the fit is far from perfect. We see two key reasons for the discrepancy between our model and experiments:

1. **High variability between experimental replicates.** The antagonism between innate response and virus (studied in the paper) is, as we understand it, the main source of this variability. All systems with mutually inhibiting pathways remain very sensitive to initial conditions and relatively small perturbations. As we can see from Appendix Figs S2A, S4A and S4C, the knockout of any key component of the innate immune system leads to a several fold increase in viral load. A 24 hour-long prestimulation with IFNβ leads to a several fold decrease in viral load (with respect to non-prestimulated cells); the effect is especially pronounced at low MOI when secondary infections are driving viral spread (Appendix Fig S5A and S5C). An additional source of variability is the condition of the viral stock, such as the presence of DVGs. DVGs can make it easier for primarily infected cells to activate IFN synthesis, thus protecting the entire cell population. Considering these factors, it is not surprising that we observe high variability between replicates.
2. **Model vs system complexity.** As pointed out by the Reviewer, the second reason is that the innate immune system is complex, whereas the model is relatively simple and is focused only on main regulatory aspects. For example, STAT1/2 activate synthesis of tens of ISGs, which in the model are represented by a single variable (despite the fact that we have measured levels of four ISGs—RIG-I, PKR, OAS1, RNase L—see Appendix Fig S3E).

However, we think that since the model predictions are compared to diverse data collected in Appendix Figs S1–S5 and Main Figs 3–5, the overall agreement is satisfactory, and sufficient for exploring virus–host interactions.

> **7.** (minor) page 6 “show that approximately 40% of RSV proteins-expressing cells…” was this a pure prediction from the model or was the model trained to try to match this data?

**Response:** The model was tuned to reflect this percentage using the `vProteins ⊣ pIRF3` feedback. We were able to achieve agreement due to the fact that both in experiments and in model simulations the average percentage is relatively stable across different MOIs and timepoints.

> **8.** (**major**) page 7 top paragraph states that vProteins inhibitions of pIRF3 is the strongest inhibitory mechanism, implying that IFN plays a minor roll. This is concerning as several studies show that without IFN, RSV viral loads and those of other viruses are significantly higher.

**Response:** Yes, disabling IFN signaling (by IFNAR KO or IFNAR+IFNLR double KO) significantly increases viral load (see Appendix Fig S4A). A similar (maybe a bit stronger) effect is observed in IRF3 KO cells (Appendix Fig S2A). In our model, inhibition of `pIRF3` leads to inhibition of `IFN` synthesis (Fig 1A). Within the model, the only difference between inhibiting `IFN` directly and through `pIRF3` is in the number of cells expressing active `pIRF3`.

> **9.** (maybe important?) fig 5 A…is there any concern that the IFNbeta concentrations are clustered only in one area of the sample while RSV and IRF3 is more evenly distributed? Is this an artifact?

**Response:** The time at which neighboring cells get infected (due to secondary infections) is about the same, and consequently so is the time at which they start producing IFN. The cell's ability to produce IFN can be enhanced when it is infected with defective viral genomes (DVGs). One can thus hypothesize that these IFN-positive cells have been infected by a single cell producing DVGs. For sure this is a very interesting area to explore.

> **10.** (**major**) the goodness of fit in the figures, such as 5b/c, is debatable. There do not seem to be clear trends in the data that the model replicates.

**Response:** Please see our response to your comment concerning Fig 3C (comment **6.**). In Fig 5B and 5C the main result is the agreement of the mean probabilities, and the demonstration that the existence and assumed strengths of inhibitions `vProteins ⊣ pIRF3` and `vProteins ⊣ IFNi` is necessary for this agreement.

> **11.** (**major**) the discussion needs to do a better job integrating the model's suggestions with known experimental observations. Studies have been performed with several cell lines wherein IFN or IRF3 have been knocked out or inhibited. How do those observations compare to the findings here? As currently written, the authors are minimizing their own work by not demonstrating its importance to the field.

**Response:** In the revised Discussion (second paragraph) we refer to other studies in which IRF3 or IFN signaling was blocked. Our data clearly indicate that IRF3 is indispensable for interferon production, and that both in IRF3 KO and IFNAR (IFNβ receptor) KO cells viral load is much higher than in wild-type cells (Appendix Fig S2A and Fig S4A, respectively). Interestingly, IFNλ receptor IFNLR KO has little or no impact on RSV spread (despite IFNλ being produced), but has impact on spread of Influenza A [see Czerkies et al, 2022, *J. Virol.*, doi:10.1128/jvi.01341-22].

## **Part III** – Minor Issues: Editorial and Data Presentation Modifications

*Please use this section for editorial suggestions as well as relatively minor modifications of existing data that would enhance clarity.*

(No Response)

# Response to the Report from Reviewer #2

## **Part I** – Summary

*Please use this section to discuss strengths/weaknesses of study, novelty/significance, general execution and scholarship.*

> **1.** In their manuscript, "Antagonism between viral infection and innate immunity at the single-cell level," Grabowski et al. use high resolution in vitro data of respiratory syncytial virus (RSV) infection of A549 cells to construct a stochastic model of virus infection dynamics and the consequent cellular innate type I/III interferon (IFN) response. The model describes the reciprocal relationships of viral antagonism of the cellular IFN response, and cellular antagonism of viral replication. Among several informative vignettes, the model predicts that in RSV infection in vitro, a relatively small number of infected cells produce IFN, although IFN-producing cells are considerably enriched for infected cells.
>
> The manuscript is extremely well-written and does an excellent job in communicating relatively complex modeling topics and interpretations such that they would be accessible

> to a general virology audience without sacrificing important details. The study addresses important problems regarding the dynamics of host-virus interactions and/or antagonism at single cell resolution, and importantly makes good use of empirical data to construct an informative mathematical model. The model is generally convincing, with plausible descriptions of the reciprocal antagonism between RSV and target cells. However, while informative and effective, it is not clear how much novel insight the model uncovers regarding RSV infection dynamics. Moreover, there is limited exploration and/or discussion of alternative mechanistic explanations for the experimental data and/or its incorporation into relationships modeled. Altogether, specific issues with the study are generally minor (listed below), and many can likely be addressed with additional explanation, discussion, and/or model testing.

**Response:** We are glad to receive an overall favorable assessment of our study. We think that the main insight gained from the model is the elucidation of mutually inhibiting interactions between the virus and host cells. The result of these interactions is that in general cells either produce interferons and signal to neighboring cells allowing them to enter antiviral state, or express viral proteins which allow for infection of neighboring cells. The model explains that this single-cell regulation is strongly influenced by population context: interferon prestimulated cells have a higher chance to slow down viral protein accumulation, produce interferon and thus send the warning signal to neighboring cells.

## **Part II** – Major Issues: Key Experiments Required for Acceptance

*Please use this section to detail the key new experiments or modifications of existing experiments that should be absolutely required to validate study conclusions.*

(No Response)

## **Part III** – Minor Issues: Editorial and Data Presentation Modifications

*Please use this section for editorial suggestions as well as relatively minor modifications of existing data that would enhance clarity.*

> **2. Model components.**
>
> The model includes sensible components that would be anticipated (and validated) to impact virus-cellular dynamics, including viral RNA accumulation, viral protein accumulation, phosphorylation of IRF3 (pIRF3), IFN production, and phosphorylation of STAT1/2 (pSTATs). However, viral sensing and downstream signaling can induce additional intra-/inter- cellular signaling cascades beyond IFN that can impact viral replication and cell state. While the model is necessarily (and appropriately) reductionist, have the authors considered additional, non-IFN signaling pathways that could be induced

> by viral infection (e.g. NfKB signaling, IL1 production, etc.)? In addition, pIRF3 is known to induce directly the expression of several antiviral effector genes (i.e. other than IFNs) that can restrict viral functions in the infected/sensing cell. However, the model does not include a virus-inhibitory interaction for the pIRF3 term. This should be tested and/or justified.

**Response:** Indeed, NF-κB is activated jointly with IRF3 (see new Appendix Fig S7, for convenience also pasted below, as well as, in the case of NDV infection, Figure S6 in Rand et al., 2012, *Mol. Syst. Biol.*, doi:10.1038/msb.2012.17) and both NF-κB and IRF3 lead to activation of antiviral effector genes (in addition to those activated by interferons via STAT signaling).

IRF3 alone induces expression of several genes — including ISG56/IFIT1, ISG54/IFIT2, and ISG60/IFIT3 — in an IFN-independent manner through the IFN-stimulated responsive elements (ISREs) [Grandvaux et al., 2002, *J. Virol.*, doi:10.1128/jvi.76.11.5532-5539.2002]. RSV is restricted in an IFIT1/2/3-dependent manner [Drori et al., 2020, *Viruses*, doi:10.3390/v12101171]. IFIT1 was characterized as a mediator of negative-feedback regulation of virus-triggered induction of type-I IFNs [Li et al., 2009, PNAS, doi:10.1073/pnas.090081810], however earlier it was shown that IFIT1 jointly with IFITM1 inhibit Hepatitis C virus replication [Raychoudhuri et al., 2011, *J.Virol.*, doi: 10.1128/JVI.05633-11], which makes the overall role of IFIT1 elusive. Interestingly, RSV NS1 impinges on expression of IFIT2/3 [Pei et al., 2021, *Cell Rep.*, doi:10.1016/j.celrep.2021.109803; van Royen, 2022, *J.Virol.*, doi:10.1128/jvi.01297-22]. The antagonism between solely-IRF3-induced IFITs and RSV is not accounted for by the model.

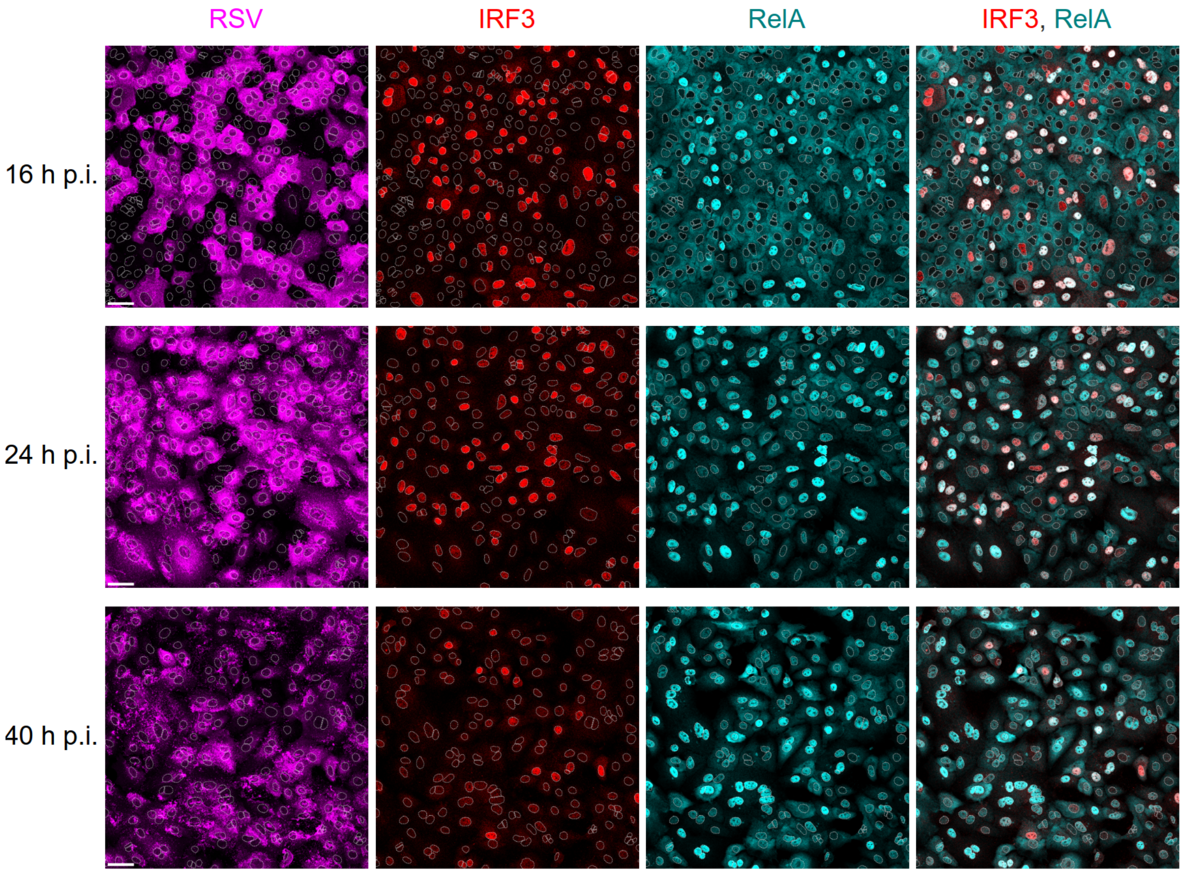

NF-κB, synergistically with NF-IL6, activates transcription of proinflammatory interleukins IL-6 and IL-8 [Matsusaka et al., 1993, PNAS, doi:10.1073/pnas.90.21.10193]. Their primary role is regulation and attraction of immune cells, which are however absent in our experiments.

Our data may suggest that IRF3 supresess viral spread not only by triggering interferon synthesis. IRF3 KO leads to a greater increase of the RSV load at 48 h p.i. (Appendix Fig S2A) than IFNAR+IFNLR double KO (Appendix Fig S4A). Comparable to IRF3 KO is the effect of RIG-I KO or MAVS KO; both these knockouts block IRF3 activation. In these three cell lines (IRF3 KO, RIG-I KO, MAVS KO) the viral load at 48 h p.i. is high enough to lead to nearly complete degradation of STAT2 protein (it is known that RSV NS1 and NS2 proteins target STAT2 for degradation, see, e.g., Elliott et al., 2007, *J. Virol.*, doi:10.1128/JVI.02303-06), which is not observed in cells with IFNAR+IFNLR double KO (or single KOs of these receptors).

We discuss these potentially important yet omitted interactions in Discussion (seventh paragraph).

> **3. STAT effects on viral replication.**
>
> Modeling suggests that STAT signaling has a minimum effect of RSV propagation in this system. The authors note the “The lack of influence of the STAT signaling inhibition by RSV (nonstructural) proteins on RSV replication observed in the model suggests that this interaction may be implicated in regulatory processes not included in the model.” Given that RSV has evolved mechanisms by which to antagonize STAT signaling and consequent ISG expression (including recently described nuclear NS1 association with ISG and other gene regulatory regions, Pei et al, Cell Reports, 2021), this seems somewhat surprising, and warrants additional discussion.

**Response:** STAT activation, leading to synthesis of ISGs, is key before a cell gets infected and viral proteins are produced. In Appendix Fig S5 we show that pre-stimulation with IFN significantly reduces viral load, demonstrating the importance of STAT signaling. We also show that knockouts of IRF3, RIG-I, MAVS, IFNAR, STAT1 and STAT2 (all individually blocking formation of phospho-STAT1/2 heterodimers) promote viral spread (Appendix Fig S2A, S4A and S4C). However, our model suggests that inhibition of STAT activation by RSV in an infected cell has a minimal effect on viral spread. The reason is that at the point at which the cell is already producing viral proteins (capable of inhibiting STAT signaling) viral replication cannot be stopped. Additionally, inhibition of STAT activation has a slow effect on ISGs if they are already accumulated, because these proteins are stable (Appendix Fig S3E).

We note that our original phrasing could have been misleading, because it could have suggested to the reader that all inhibitions of STAT signaling have no effect on viral spread. We have reworded this sentence in the revised Manuscript.

It is plausible that disabling production of ISG-encoded proteins redirects ribosomes to production of viral proteins, enabling efficient generation of viral progeny. Consistently with this hypothesis, RSV has been shown to take over the global control of host cell translation [see, e.g., Groskreutz et al.,

2010, *J. Biol. Chem.*, doi:10.1073/pnas.90.21.10193]. We include this hypothesis in revised Discussion (fourth paragraph).

In the case of RSV, we do not observe cell death during the first 48 h p.i.; however, in the case of other viruses, such as influenza A virus (IAV), infection can lead to a relatively fast cell death. Fast cell death (before new virions are assembled) may inhibit virus spread [Orzalli & Kagan, 2017, *Trends Cell Biol.*, doi:10.1016/j.tcb.2017.05.007]. STAT1 was reported to promote cell death [Kim & Lee, 2007, *Cell Signal.*, doi:10.1016/j.cellsig.2006.09.003], so inhibition of STAT activity in infected cells is expected to promote virus spread.

> **4. Syncytia formation.**
>
> Consistent with experimental data and well-established mechanisms of the RSV life-cycle, the model only allows for infection of neighboring cells. However, in addition to "standard" cell-to-cell transmission, RSV can form eponymous syncytia, which can include the "merging" of infected cells with uninfected cells. For purposes of modeling viral spread, syncytia formation likely represents a distinct mode of transmission (e.g. potentially with considerable levels of "preformed" viral RNA and proteins "transferred" to the newly infected cell). Did the authors observe syncytia formation in the A549 infections used to support the model? In addition, how might the model be affected by/take into account syncytia formation?

**Response:** Yes, we observe formation of syncytia. We think that by assuming neighbor-to-neighbor infections we implicitly account for syncytia—although not fully. Direct transfer of both viral RNA and proteins may lower the chance of interferon activation and accelerate virion production. Instead of modeling syncytia formation, we have tuned our model to reflect the observed rate of infecting neighboring cells. This is a valid point and we refer to it in subsection *Model justification and choice of parameters* in *Methods*.
